# Computational Prediction of Potential Inhibitors of the Main Protease of SARS-CoV-2

**DOI:** 10.3389/fchem.2020.590263

**Published:** 2020-12-23

**Authors:** Renata Abel, María Paredes Ramos, Qiaofeng Chen, Horacio Pérez-Sánchez, Flaminia Coluzzi, Monica Rocco, Paolo Marchetti, Cameron Mura, Maurizio Simmaco, Philip E. Bourne, Robert Preissner, Priyanka Banerjee

**Affiliations:** ^1^Institute of Physiology, Charité–University Medicine Berlin, Berlin, Germany; ^2^METMED Research Group, Physical Chemistry Department, Universidade da Coruña (UDC), A Coruña, Spain; ^3^Structural Bioinformatics and High-Performance Computing (BIO-HPC) Research Group, Universidad Católica de Murcia (UCAM), Murcia, Spain; ^4^Department of Medical and Surgical Sciences and Biotechnologies, Sapienza University of Rome, Latina, Italy; ^5^Unit of Anesthesia and Intensive Care Medicine, Sant' Andrea University Hospital, Rome, Italy; ^6^Department of Clinical and Surgical Translational Medicine, Sapienza University of Rome, Rome, Italy; ^7^Department of Biomedical Engineering and School of Data Science, University of Virginia, Charlottesville, VA, United States; ^8^Department of Neurosciences, Mental Health and Sensory Organs, Sapienza University of Rome, Rome, Italy; ^9^Advanced Molecular Diagnostic Unit, Sant' Andrea University Hospital, Rome, Italy; ^10^Institute of Physiology and Science-IT, Charité–Universitätsmedizin Berlin, Corporate Member of Freie Universität Berlin, Humboldt-Universität zu Berlin, and Berlin Institute of Health, Berlin, Germany

**Keywords:** virtual screening (VS), drug repurposing and molecular docking, SARS-CoV-2, COVID-19, computational drug discovery, molecular dynamics

## Abstract

The rapidly developing pandemic, known as coronavirus disease 2019 (COVID-19) and caused by the severe acute respiratory syndrome coronavirus 2 (SARS-CoV-2), has recently spread across 213 countries and territories. This pandemic is a dire public health threat—particularly for those suffering from hypertension, cardiovascular diseases, pulmonary diseases, or diabetes; without approved treatments, it is likely to persist or recur. To facilitate the rapid discovery of inhibitors with clinical potential, we have applied ligand- and structure-based computational approaches to develop a virtual screening methodology that allows us to predict potential inhibitors. In this work, virtual screening was performed against two natural products databases, Super Natural II and Traditional Chinese Medicine. Additionally, we have used an integrated drug repurposing approach to computationally identify potential inhibitors of the main protease of SARS-CoV-2 in databases of drugs (both approved and withdrawn). Roughly 360,000 compounds were screened using various molecular fingerprints and molecular docking methods; of these, 80 docked compounds were evaluated in detail, and the 12 best hits from four datasets were further inspected *via* molecular dynamics simulations. Finally, toxicity and cytochrome inhibition profiles were computationally analyzed for the selected candidate compounds.

## Introduction

A novel coronavirus (CoV), known as the severe acute respiratory syndrome coronavirus 2 (SARS-CoV-2), began spreading among humans in December 2019 in the city of Wuhan, China, causing a major outbreak of often-fatal pneumonia (Wu et al., 2020). The rapid expansion of SARS-CoV-2 has been labeled a pandemic by the World Health Organization (WHO), and the global crisis has continued to devastate both human health and national economies (WHO^1^). The symptoms associated with most instances of this infection include fever, dry cough, fatigue, shortness of breath and respiratory issues (Wu et al., 2020), and deterioration of some sensory modalities (e.g., taste, smell); a smaller fraction of cases also present with other symptoms, e.g., conjunctivitis (presumably another mode of transmission, too) (Scasso et al., 2018). With SARS-CoV-2, aggressive human–human transmission has occurred, yielding exponential growth in the number of detected cases. The disease has now been termed as “coronavirus disease 2019” (COVID-19) (Zhang L. et al., 2020). At present, the number of confirmed cases reported internationally has reached 15,581,009, with 635,173 deaths reported^2^. As of yet, no potent drug or vaccine has been reported (or approved) to treat individuals infected with SARS-CoV-2; only symptomatic treatment has been given to the most critically ill patients. A surge in activity among the scientific community has advanced research efforts toward the development of therapeutic intervention and finding viral drug targets; currently, 36 repurposed drugs are already used in experimental (unapproved) treatments for COVID-19, and 432 drugs are being tested in ongoing clinical trials^3^. In addition, there are 23 candidate vaccines in clinical evaluation and 140 candidate vaccines in preclinical evaluation^4^. Initial results from a phase 1 clinical trial are already available for a vaccine known as mRNA-1273 (Jackson et al., 2020). Recent reports suggest that some U.S. Food & Drug Administration (FDA)-approved drugs, specifically remdesivir (which inhibits viral RNA polymerase) (Al-Tawfiq et al., 2020) and lopinavir and ritonavir (HIV protease inhibitors) (Cao et al., 2020), may be effective against SARS-CoV-2. Remdesivir exhibits an antiviral activity with an EC_50_ of 0.77 μM against SARS-CoV-2, and shorter recovery times (vs. a placebo group) were found for adults hospitalized with COVID-19 and treated with remdesivir; additionally, those patients showed fewer infections of the respiratory tract (Beigel et al., 2020). In March 2020, the WHO launched a “solidarity clinical trials” of repurposed drugs and experimental candidates, wherein testing of the three aforementioned drugs was supplemented with testing of the antimalarial compounds chloroquine and hydroxychloroquine^5^. In July 2020, WHO decided to discontinue the hydroxychloroquine and lopinavir/ritonavir trials, as these compounds yielded little to no reduction in the mortality of hospitalized COVID-19 patients when compared to standard of care^5^. Currently, dexamethasone—an anti-inflammatory drug approved to treat COVID-19 patients in the UK and Japan—also has been reported, in an unpublished study, to reduce mortality among COVID-19 patients hospitalized with severe infection (Horby et al., 2020).

The earliest-discovered CoVs do not correspond to those strains that are the causative infectious agents in recent outbreaks, including COVID-19 (Khedkar and Patzak, 2020). The first “coronavirus” (to be termed as such) was isolated from chicken in 1937; human CoVs were identified years later, in the mid-1960s^6^. These viruses belong to the taxonomic family *Coronaviridae*, which are single-stranded, positive-sense RNA viruses of ~29.9 Kb genomic length (Khedkar and Patzak, 2020). The CoVs encode more than a dozen proteins, some of which have been identified as critical for viral entry and replication (Muramatsu et al., 2016). Among the structural proteins encoded by the CoV genome, four proteins are of special interest from the perspective of therapeutics and drug design—namely, the *spike* (S), *envelope* (E), *membrane* (M), *and nucleocapsid* (N) proteins. The S, E, and M proteins are housed in the membranes of these enveloped virions. The M and E proteins are actively involved in viral coat assembly, while the N protein is involved in compacting the RNA genome. The most-studied proteins thus far have been a papain-like protease (PLpro), a 3C-like protease (3CL^pro^), an endosomal protease, and the spike protein (Yang and Wang, 2020).

At the molecular level, CoVs are known to gain cellular entry *via* the S protein (Anand et al., 2003). Viral entry depends on the binding of the surface unit S1 of the S protein to a surface-exposed cellular receptor in the host, thereby supporting the process of viral attachment to target cell surfaces (Muramatsu et al., 2016). The 3CL protease (3CL^pro^), also known as M^pro^, is the main protease produced by the CoV; it plays a key role in viral replication (Wu et al., 2020). Most of the functional proteins of CoVs are encoded by specific genes, which are first translated into polyproteins that are then cleaved by the viral 3CL^Pro^ or by PLpro. This stage of the viral replication cycle yields the RNA-dependent RNA polymerase (RdRp), along with multiple other proteins that play roles in virus replication, transcription, and translation. Inhibiting the activity of the main CoV protease would presumably block viral replication (Yang and Wang, 2020). Thus, 3CL^pro^ is considered a potential drug target for COVID-19. In addition, targeting the main protease for inhibition is an appealing strategy because it may well be thought that this would inactivate the virus in different cell types and in different organs—regardless of the various matches between receptors/host proteases (on the cell membrane) that underlie viral entry in a cell- or tissue-specific manner (Zhang L. et al., 2020).

**Figure 1:**
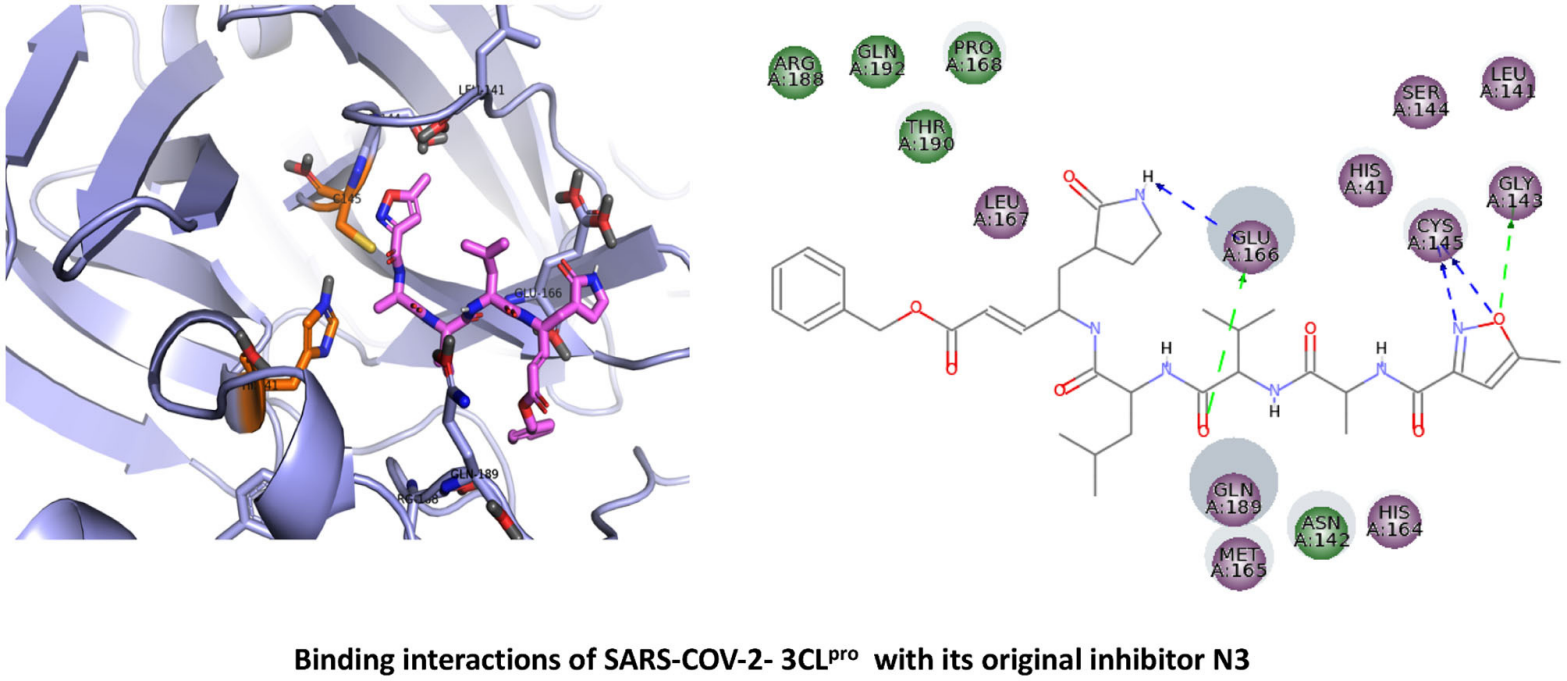
Binding interactions of severe acute respiratory syndrome coronavirus 2 (SARS-CoV-2) - 3CL^pro^ with its original inhibitor N3. The hydrogen bond interactions with protein backbone are indicated in blue dotted lines, and hydrogen bonds with the side-chain atoms are shown in green dotted lines.

Because of its mechanistic significance, 3CL^pro^ is now a central target for the development of effective inhibitors (antiviral drugs) against both SARS-CoV-2 and other known CoVs (Anand et al., 2003). The X-ray crystal structure of 3CL^pro^(M^pro^) from SARS-CoV-2 (PDB code: 6LU7) reveals a protein comprised of three primary domains (Jin et al., 2020). The first domain (Domain I) consists of amino acid residues 8–101; the second domain (Domain II) maps to residues 102–184; and the third domain (Domain III) mainly consists of residues 201–306, largely as a cluster of α-helical conformations (Jin et al., 2020). The substrate-binding region of 3CL^pro^, located between Domains I and II, includes residues His41 and Cys145. Visual inspection of the binding site structure confirms that peptide-type inhibitors attach to the active site cysteine: a previously identified peptidomimetic inhibitor, “N3,” interacts irreversibly with this site and engages in supporting interactions with subsites (S1, S2, and S4) (Jin et al., 2020). The S1 subsite contains residues His163, Glu166, Cys145, Gly143, His172, and Phe140, while the S2 subsite consists mainly of Cys145, His41, and Thr25; these amino acid types are compatible with favorable non-bonded contacts such as electrostatic interactions and van der Waals (apolar/dispersive) forces. There are two additional subsites (S3-S5), consisting of Thr190, Gln192, Glu166, Met49, Leu167, Gln189, and Met165 (Anand et al., 2003). A ligand interaction diagram drawn from the 3D crystal structure (Figure 1) illustrates that this particular N3 inhibitor engages in multidentate hydrogen bonds with Glu166 (as both donor and acceptor). In addition, there are close—and presumably energetically favorable—contacts between moieties of N3 and Gly143 and the catalytic Cys145 (both of the S1 subsite).

The traditional drug discovery and development pipeline is generally a quite time-consuming endeavor, taking upward of ≈10–15 years (Turanli et al., 2019; Kupferschmidt and Cohen, 2020). Computational drug “repurposing” is an effective approach to accelerate this timescale by finding new uses for existing (and already approved) drugs (McNamee et al., 2017). Computational approaches to drug discovery, particularly as part of a repurposing strategy/framework, can fasten the drug development process and alleviate the burdens of traditional approaches—features that are especially critical in the context of a pandemic. Such computational approaches have been used to identify candidate drugs for several infectious diseases, including Ebola, Zika, dengue, and influenza (Cha et al., 2018). Many computational methods are available to examine the key interrelationships between chemical structure, biological/physiological systems, interactions between chemicals and (bio) molecular targets, and, finally, the ultimate therapeutic endpoints and diseases (Metushi et al., 2015).

For COVID-19, several recent studies have reported the computational screening of inhibitors for specific single targets, such as either the main protease (the aforementioned 3CL^pro^/M^pro^) or the spike protein (Shiryaev et al., 2017; Botta et al., 2018; Pizzorno et al., 2019; Ton et al., 2020; Wang, 2020; Zhang D.-h. et al., 2020). To the best of our knowledge, the work reported here is the first study to report screening results including databases like Super Natural II^7^, as well as the TCM (Chen, 2011)^8^, and repurposing-focused databases, such as SuperDRUG2^9^ and WITHDRAWN database^10^. Additionally, our final selection of lead compounds is based on visual inspection and analysis of ligand interactions with crucial residues of the target (3CL^pro^), thereby implicitly incorporating human expertise and clinical insights into our workflow (once the number of candidates becomes manageable for manual analysis). The final 12 best candidates were further evaluated using molecular dynamics (MD) simulation studies, and clinical feasibility for repurposed drugs was further investigated.

The general approach used in this study is based on an integrated pipeline: we include a virtual screening of cheminformatics-driven databases, and we employ molecular similarity and molecular docking to identify promising drug target pairs. Additionally, MD simulation studies of selected compounds were performed to select the best candidates for main protease inhibitors and evaluate their stability and strength of interactions. Our screening is aimed at dual objectives: (i) first, to find potential new candidates in the Super Natural II^7^ and TCM (Chen, 2011)^8^, and (ii) second, to identify promising repurposing candidates in the SuperDRUG2^8^ and WITHDRAWN databases^10^.

## Materials and Methods

To facilitate the rapid discovery of target inhibitors with real clinical potential, we have employed a prediction strategy that is based on the fundamental principle of “neighborhood behavior” (Patterson et al., 1996) implemented as a computational pipeline that utilizes both *ligand-based* [two-dimensional (2D) chemical space of small molecules] and *structure-based* [protein three-dimensional (3D) spaces and features]. Our computational predictions use the first resolved crystal structure of SARS-CoV-2 main protease (at a resolution 2.16 Å). Currently, several crystal structures of the SARS-CoV-2 main protease have been experimentally determined (approximately 175 structures)^11^. The methodological details of our approach and computational methods are described below.

### Databases

In order to broadly screen, we utilized four different database resources: (i) SuperNatural II, a database of natural products^7^; (ii) The Traditional Chinese Medicine (SuperTCM) database, an in-house (unpublished) database that is manually created from other databases and Chinese literature (Chen, 2011)^8^; this comprehensive database built at the Charite–University of Medicine^12^^,^^13^ covers all aspects of traditional Chinese medicine mainly derived from medicinal plants, and it encompasses pharmaceutical recipes up to molecular ingredients. The database was manually curated by domain experts, and Chinese plant-based drugs were mapped to their plant of origin, common non-scientific names and scientific names, targets, Kyoto Encyclopedia of Genes and Genomes (KEGG) pathways^14^, as well as the traditional Chinese traditional recipes. (iii) We also used SuperDRUG2, a one-stop resource for approved/marketed drugs^8^; (iv) And, finally, we also used WITHDRAWN, a resource for withdrawn and discontinued drugs^10^. Overall, we utilized more than 360,000 compounds for virtual screening and initial filtering purposes (Table 1).

**Table 1:** Databases and total number of compounds used in this study.

**Database**	**Compounds**	**Total number of compounds used in this study**
SuperDRUG2	Approved and marketed drugs	3,992
WITHDRAWN	Withdrawn or discontinued drugs	626
TCM	In-house database of compounds related to Traditional Chinese Medicine	28,974
Super Natural II	Natural compounds	325,508

The potential inhibitors are screened mainly from phytochemical databases because the literature suggests that seven out of 10 synthetic agents approved by the U.S. FDA are modeled on a natural product parent (Newman and Cragg, 2016). There is an urgent need to identify novel active chemotypes as leads for effective antiviral therapy for COVID-19 infections. Similarly, several thousands of plant extracts have been shown to possess *in vitro* antiviral activity with little overlap in species between studies (Chen, 2011). Promising docking outcomes have been executed in this study, which evidenced the worth of these selected chemical compounds from Super Natural II and TCM databases for future drug development to combat CoV diseases.

Additionally, drug databases from both approved and withdrawn chemical spaces were screened to support reuse of already available drugs for new indications such as COVID-19 therapy when they have been originally developed for specific diseases.

### Ligand-Based Screening

We screened the molecular libraries from these databases based on ligand similarity, which rests upon the assumption that “structurally similar compounds might have similar biological properties” (Stumpfe and Bajorath, 2011). As many chemical-based fingerprint methods are in widespread use, based on the performances of various structural similarity measures, a 2D similarity screening protocol was designed, and three different types of molecular fingerprints were initially chosen for comparison and performance evaluation: MACCS (Durant et al., 2002), Extended Connectivity Fingerprints (ECFP-4) (Rogers and Hahn, 2010), and E-state (Hall and Kier, 1995). We found that the performance of the MACCS fingerprint surpasses that of ECFP-4 and E-state in an approved drug dataset, with a higher Tanimoto score threshold (from 0.73 to 0.83) than ECFP-4 (Supplementary Table 1). MACCS ranked the similarity scores higher for co-crystalized ligands and drugs like lopinavir, angiotensin II, and indinavir (compounds that were previously reported as potential inhibitors of the main protease 3CL^pro^) (Contini, 2020; Nutho et al., 2020). Using this small set of active compounds, we tried to find the optimal similarity cutoff as well as optimal chemical fingerprint to yield the best balance of precision vs. recall.

Chemical-based fingerprints were calculated using RDKit^15^ nodes in KNIME (Berthold et al., 2008), and pairwise similarities were calculated for all the datasets.

The lead compounds used in the similarity search for targeting the 3CL^pro^ of SARS-CoV-2 were obtained from the Protein Data Bank (PDB)^5^. The original ligands from PDB structures 6LU7 and 6Y2F were considered as lead compounds. The first ligand (“N3”) is a peptidomimetic irreversible inhibitor and was found covalently bonded to Cys145. This interaction is reported to be essential for preserving the protease's S1 pocket in the right shape and also for the active conformation of the enzyme (Zhang L. et al., 2020). The N3 ligand interacts with the catalytic center of the target proteases through two hydrogen bond interactions. It was observed that the pyridine ring might have some steric clash with the side chain of Gln 189 (Zhang L. et al., 2020). The reported α-ketoamide ligand-bound X-ray crystal structure of SARS-CoV-2 Mpro (PDB ID: 6Y2F) forms hydrogen bonds with the Ser of chain B and Glu166 of chain A (Zhang L. et al., 2020). This interaction is reported to be essential for keeping the S1 pocket in the right shape for ligand–receptor interactions and also for the active conformation of the enzyme (Zhang L. et al., 2020). It interacts with the catalytic center of the target proteases through two hydrogen bond interactions. It was observed that the pyridine ring might have some steric clash with the side chain of Gln189 (Zhang L. et al., 2020). The compounds that showed the highest similarity considering structural properties were finally chosen for molecular docking studies. For each of the dataset and each of the lead compounds, the 10 best hits were chosen (Supplementary Tables 7, 8). The Tanimoto score values for selected compounds range between 0.63 and 0.83. It is also possible that a “false similar” or “false active” pair of molecules could occur, featuring structural similarity but dissimilarity in terms of their biological activities; to assess this possibility regarding activity profiles, further molecular docking and MD simulation studies were conducted.

### Structure-Based Screening

To further refine the list of candidate compounds and select the top hits, molecular docking calculations were carried out using the GOLD software (version 5.7.2) (Jones et al., 1997). This code uses a genetic algorithm to sample the ligand's conformational space, making it particularly suitable for docking flexible ligands with numerous rotational degrees of freedom (Jones et al., 1997). In addition, the GOLD scoring function was used to rank the compounds, with the number of docked poses to be generated set to 10. In the present study, the co-crystal structures of 3CL^pro^ of SARS-CoV-2 (PDB 6LU7) was selected as the starting structure for docking calculations. Residues that are proximal (within a 10 Å radius) to the original ligand co-crystallized in 6LU7, along with binding site residues (as defined in the literature) were taken to be the active site for docking calculations. Thus, the final docking protocol incorporates information from the successful re-docking of the original ligand to the target.

A total of 80 compounds (top 10 screened compounds for each lead compound, selected using ligand-based screening from four different databases) were docked into the main protease protein 3CL^pro^. Based on interactions with key residues (His41 or Cys145), present in the binding cavity of the 6LU7 crystal structure (and based on visual inspection of the ligand–receptor binding interactions), the top 3 best candidates were selected per database. Therefore, our final list includes the 12 best candidates based on this computational screening protocol. Two-dimensional ligand–receptor binding interaction maps were computed using Accelrys Discovery Studio (version 4.5)^16^. The 3D interactions and structural illustrations were created using PyMOL (version 2.3.5)^17^. After the molecular docking analyses, MD simulations were performed for the best 12 screened candidates, enabling us to evaluate the dynamical stability of the bound/docked complexes, at least on the timescale of the MD trajectories.

### Molecular Dynamics Simulation

Analyzing the dynamic evolution of the ligand-bound system helps us predict the stability of those interactions that we first detected via ligand-based virtual screening (LBVS) and structure-based virtual screening (SBVS); ideally, the MD faithfully recapitulates the real (physiological) environment of such interactions. Ligand–protein interactions at the binding site can be monitored for a period of time, so that ligands with more temporally stable poses can be detected and proposed as better candidates for 3CL^pro^ inhibition. Thus, after our database screening stages, MD simulations of the different complexes were computed using the GPU version of Desmond included with Maestro suite 2019.4 (Schrödinger LLC)^18^ on a workstation with a NVIDIA QUADRO 5000. The various drug/receptor complexes were solvated in an aqueous environment in a cubic box with a minimal distance of 10 Å between the biomolecule and the box boundary (for periodic boundary conditions). Next, systems were neutralized and maintained in 0.15 M NaCl. The OPLS3 force field and the TIP3P-TIP4P water model were employed (Mark and Nilsson, 2001). Initially, the systems were simply energy-minimized for 2,000 time steps. Next, systems were allowed to execute free dynamics in the NPT ensemble; pressure was controlled using the Martyna–Tobias–Klein methodology, and the Nose–Hoover thermostat was employed to maintain the system near 310 K. Production-grade MD trajectories were extended to a total duration of 100 ns per system. MD trajectories were characterized in terms of the root-mean-square deviation (RMSD) of fluctuations of ligand and enzyme, particularly in terms of the main interactions with the top interacting residues. The trajectories were also used to assess the stabilities of the protein secondary structures (in complex with potential inhibitor) by plotting RMSDs. Additionally, to estimate the relative binding free energies of the 12 final compounds and also N3 ligand to the macromolecule, molecular mechanics–generalized Born surface area (MM-GBSA) method was applied. The MM-GBSA method is based on the difference between the free energies of the protein, ligand, and the complex in solution. The free energy for each species involved in the reaction (ligand, protein, and ligand–protein complex) is described as a sum of a gas-phase energy, polar and non-polar solvation terms, and an entropy term. In our computational protocol, the MM-GBSA method is used to calculate the free energy (dG) related to all poses obtained in the MD simulation by using the OPLS3 as implemented in the Small-Drug Design Suite of Schrodinger (Kollman et al., 2000; Greenidge et al., 2013).

### Absorption, Distribution, Metabolism, Elimination, and Toxicity Properties

The docked compounds were further filtered using the standard ADMET (Absorption, Distribution, Metabolism, Elimination, and Toxicity) pharmacokinetic properties. Computational toxicity analysis was performed using the ProTox-II methods^19^. The ProTox-II web server currently holds 40 different predictive models, incorporating chemical similarity, fragment propensities, most frequent features, pharmacophores, and machine learning for toxicity prediction. The acute toxicity value of the ProTox-II method is divided into six classes based on a globally harmonized system of classification of labeling of chemicals (GHS). The classes are described as: Class I: fatal if swallowed (LD_50_ ≤ 5); Class 2: fatal if swallowed (5 < LD_50_ ≤ 50); Class 3: toxic if swallowed (50 < LD_50_ ≤ 300); Class 4: harmful if swallowed (300 < LD_50_ ≤ 2,000); Class 5: may be harmful if swallowed (2,000 < LD_50_ ≤ 5,000); Class 6: non-toxic (LD_50_ > 5,000)^38^. Additionally, cytochrome (CYP) inhibition profiles of each compound were computed using the SuperCYPsPred web server^20^. Currently, the SuperCYPsPred web server includes 10 models for five major CYPs isoforms (including 3A4, 2C9, 2C19, 2D6, and 1A2). These cytochrome predictive models are based on machine learning methods (see Tables 2–4 in the Results section).

**Table 2:** Potential inhibitors for the main protease of severe acute respiratory syndrome coronavirus 2 (SARS-CoV-2) from Super Natural II and SuperTCM databases.

**Compound**	**Interacting residues (3CL)**	**Acute toxicity**	**Toxicity endpoints**	**CYP activity**	**Tanimoto score**	Structure^*^
N3-inhibitor	Glu166, Cys145, Gly143	Class 5	NA	CYP3A4	1	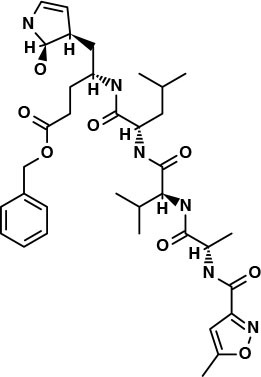
**Super Natural II database**						
SN00017653	Cys145, Gly143, Glu166, Ser144, Leu141, Thr26	Class 4	NA	NA	0.75	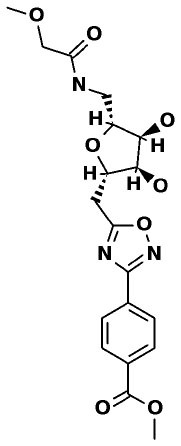
SN00019468	His41, Cys145, Gly143, Ser144, Leu141	Class 4	NA	NA	0.74	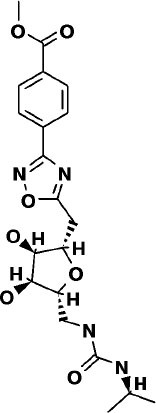
SN00303378	His41, Thr190, Gln192, Ser144	Class 3	Immunotoxic	CYP3A4	0.73	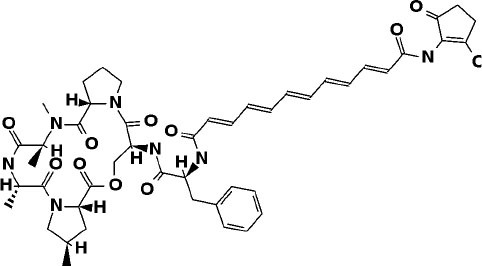
**TCM database**						
Notoamide R	Cys145, His41, His164, Gln189	Class 4	Immunotoxic	3A4	0.75	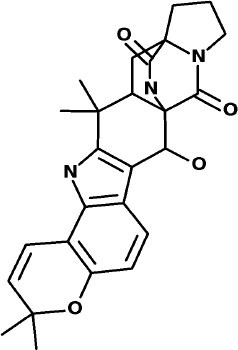
Dianthin E	Cys145, Gln189, Glu166, Ser144, Gly143	Class 4	None	None	0.74	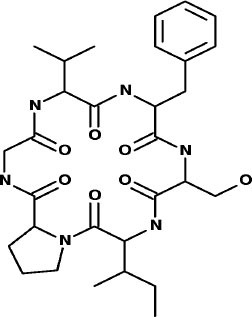
Pseudostellarin C	Cys145, His164, His41, Glu166	Class 4	Immunotoxic	None	0.77	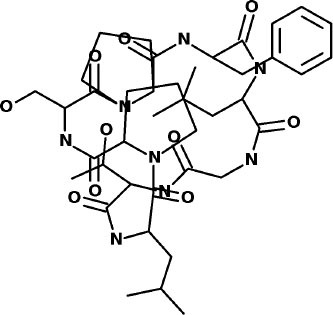

* *Structures of compounds generated with PubChem Sketcher V2.4 (Ihlenfeldt et al., 2009)*.

**Table 3:** Potential inhibitors for the main protease of severe acute respiratory syndrome coronavirus 2 (SARS-CoV-2) from SuperDRUG2 and WITHDRAWN drug databases.

**Compound**	**Interacting residues (3CL)**	**Therapeutic endpoints**	**Acute toxicity**	**Toxicity endpoints**	**CYP activity profile**	**Tanimoto score**	Structure^*^
N3-inhibitor	Glu166, Cys145, Gly143		Class 5	NA	CYP3A4	1	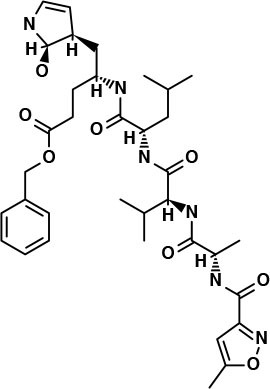
**Approved drugs database**							
Eledoisin	Ser144, Arg188, Asn142, Cys145, His41	Vasodilator	Class 5	None	None	0.72	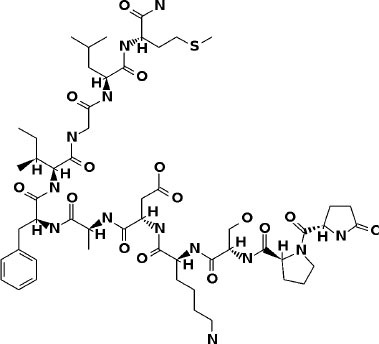
Naldemedine	His41, Cys145, Gln192, His164	Alimentary tract and metabolism	Class 4	Immunotoxic	3A4	0.72	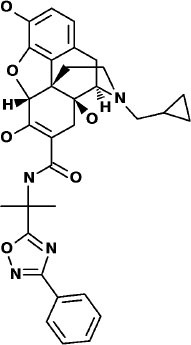
Angiotensin II	Cys145, Gln189, Asn142	Cardiac therapy	Class 5	None	None	0.73	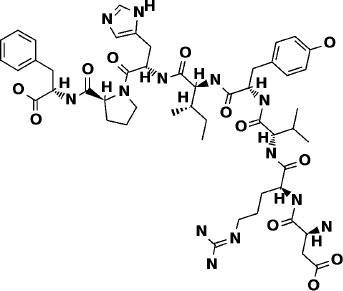
**Withdrawn drugs database**							
Saralasin	Cys145, Met165, Gln189, Arg188	Cardiac therapy	Class 5	None	None	0.71	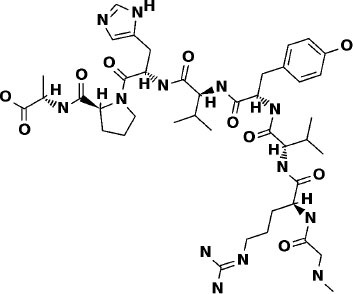
Saquinavir	Cys145, Gly143, His41, Glu166	Antiviral	Class 4	None	2C8. 2C9, 2C19, 2D6, 3A4, 3A5	0.67	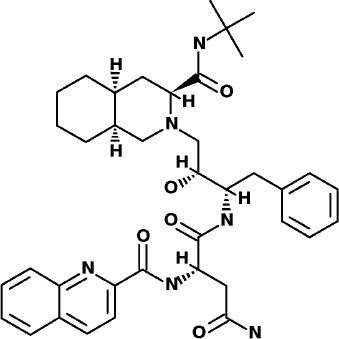
Aliskiren	Tyr54, Cys145, Ser144	Cardiac therapy	Class 5	Immunotoxic	3A4	0.63	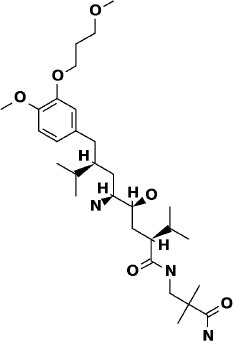

* *Structures of compounds generated with PubChem Sketcher V2.4 (Ihlenfeldt et al., 2019)*.

**Table 4:** Molecular mechanics–generalized Born surface area (MM-GBSA) calculation.

**Drug**	**dG (kcal/mol)**	**SD (kcal/mol)**
SN00019468	−54.0	13.5
SN00017653	−54.5	5.8
SN00303378	−54.3	6.4
Pseudostellarin C	−53.6	12.1
Notoamide R	−21.4	17.5
Dianthin E	−61.70	10.6
Angiotensin II	−74.9	6.8
Eledoisin	−93.3	8.9
Naldemedine	−64.2	6.7
Saquinavir	−54.3	6.4
Aliskiren	−61.2	9.9
Saralasin	−61.8	10.6
N3 inhibitor	−64.3	12.3

*dG, free energy; SD, standard deviation. The obtained results indicate the mean of the energy calculated for each pose of the simulation and its corresponding standard deviation*.

## Results

### Overview

A similarity-based approach, using structural chemical fingerprints, was used for initial screening of compounds from different databases. The crystal structure of the main protease was used as the molecular target for computational docking and protein–ligand interaction analyses; in total, visual inspections were performed for 80 compounds. The top 40 compounds for the N3 inhibitor-based docking are shown in Supplementary Table 7, and the top 40 compounds for the 6OK inhibitor-based efforts are given in Supplementary Table 8. Using our integrated approach—i.e., chemical similarity with molecular docking—a list of 12 top lead compounds were identified from four different databases. Visual inspection and selection of best candidates were based on analyses of the interaction with at least one of the two catalytic residues Cys154 or His41. The results for these compounds are shown in Tables 2, 3. Besides the selected compounds, the tables also include information on therapeutic endpoints, toxicity endpoints, and cytochrome activity profiles of the compounds; interactive residues in the protease receptor are also given. For compounds from the Traditional Chinese Medicine database, information on their respective plants (as curated from our database) is also provided, with additional information on their indications and effects (Supplementary Table 6). Furthermore, to elucidate potential molecular mechanisms in terms of ligand interactions, the top 3 compounds from each database were selected and examined via all-atom MD simulations. The 12 best candidates are described below, including the RMSD plots obtained from the MD trajectories. For the validation of the MD protocol, simulation of N3 ligand was also performed. Results are presented in Supplementary Figures 5, 6. Additional information on binding interactions from molecular docking studies (for a selected subset of 56 compounds) is provided in Supplementary Table 9, and docking scores for the N3 and O6K ligands are provided in Supplementary Table 10.

### Super Natural II Database

**Figure 2:**
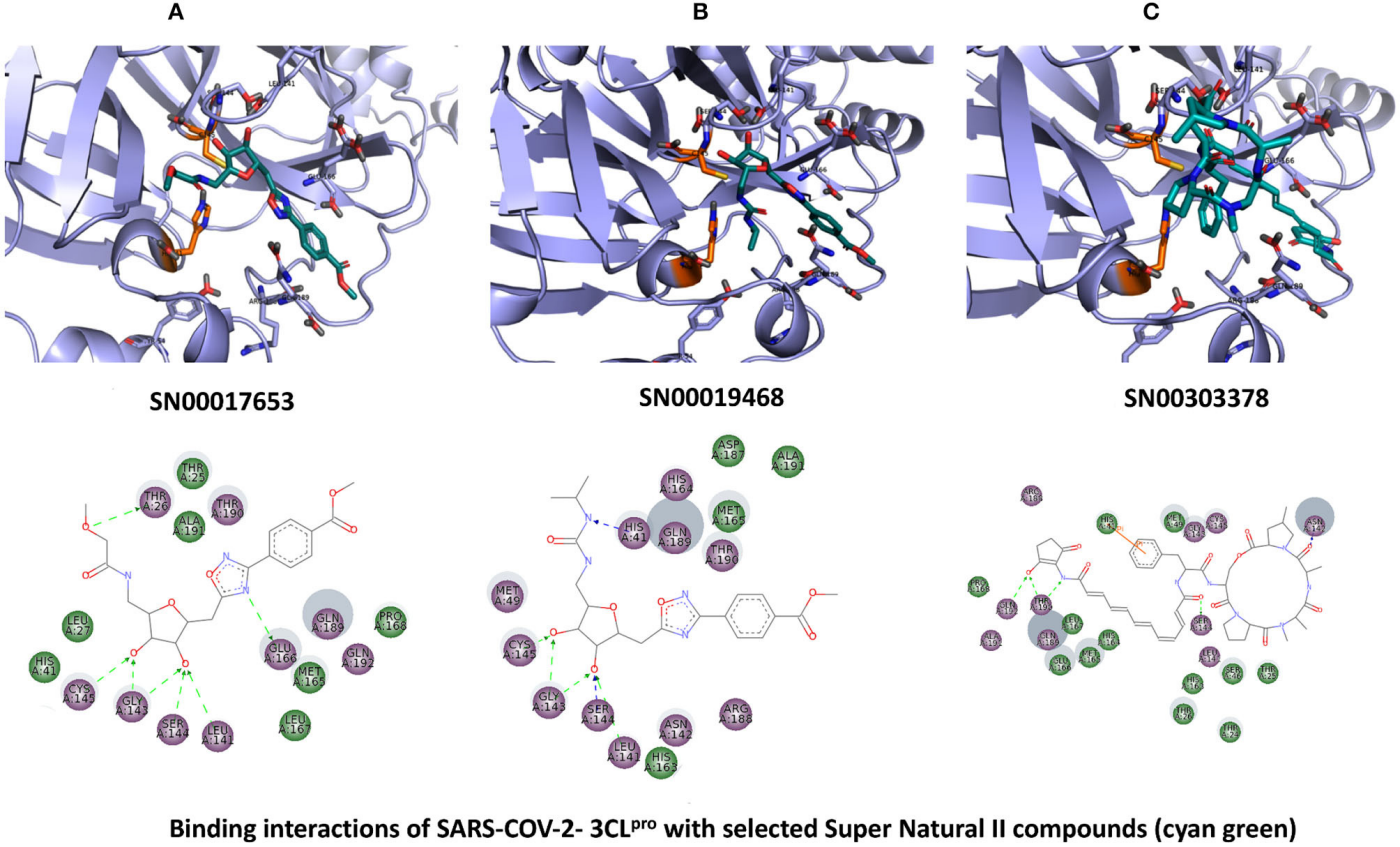
Binding interactions of severe acute respiratory syndrome coronavirus 2 (SARS-CoV-2) - 3CL^pro^ with selected Super Natural II compounds (cyan green) The hydrogen bond interactions with protein backbone are indicated in blue dotted lines, and hydrogen bonds with the side-chain atoms are shown in green dotted lines. **(A)** SN00017653. **(B)** SN00019468. **(C)** SN00303378.

The top 3 compounds we identified in the Super Natural II database are SN00017653, SN00019468, and SN00303378. The compound SN00017653 interacts *via* hydrogen bonds with the side-chain atoms of Glu166, Ser144, Leu141, Gly143, and Cys145 (Figure 2A). The second compound, SN00019468, interacts with Cys145, Gly143, and Ser144 and hydrogen-bonds with the backbone of His41 (Figure 2B). The third compound, SN00303378, interacts with His41, Asn142, Thr190, Gln192, and Ser144 (Figure 2C). In addition, the top 10 compounds from the Super Natural database are reported in Supplementary Table 2.

MD simulations reveal that SN00017653 exhibits a stable pose when interacting with 3CL^pro^, at least in terms of RMSD values of ≈2.0 and 2.8 Å for ligand and protein atoms, respectively. This compound exhibits quite stable contacts with the S2 subsite, where it interacts with Thr26 and His41. The time evolution of protein–ligand contacts shows also a weak interaction with Cys145, but this contact is highly transient. Furthermore, SN00017653 interacts with the S3 and S5 sub-pockets by binding Glu166, Gln189, and Thr190. Interactions with Glu166 persist after 100 ns, and it shows a high interaction score in comparison with the other key residue, so it is considered the most important interaction (**Figure 6**, Supplementary Figure 1).

Compound SN00019468 shows high ligand RMSD fluctuations, especially during 50–100-ns trajectory. Examination of protein-ligand interaction contacts reveals that SN00019468 samples two distinct positions. During the first 50 ns, this ligand interacts with Thr26, His164, and Gln189, being only contact important for 3CL^pro^ inhibition, so it is part of the S3–S5 subsite. During the last 50 ns, these contacts evolve to an interaction with the S2 subsite by means of His41, but this interaction is not persistent, maintained during 50–90 ns, but lost after the 100-ns simulation. The poor stability of SN0019468 in the binding site suggests it is less likely to be an effective 3CL^pro^ inhibitor (**Figure 6**).

The ligand and the protein RMSDs are stable for compound SN00303378, indicating a stable position of this compound when bound to the protein. The pattern of protein contacts (**Figure 6**) shows an interaction with His41, but it fluctuates along the 100-ns simulation (being more important in the ≈50–70-ns range). Also, the RMSD is stabilized during this period, with closely matched values for protein and ligand atoms. Glu166 engages in the clearest interactions, persisting along the full trajectory. Nevertheless, SN00303378 is not considered a strong candidate to inhibit 3CL^pro^ because of its paucity of contacts with other key residues of the main protease (**Figure 6**).

### Traditional Chinese Medicine Database (SuperTCM)

**Figure 3:**
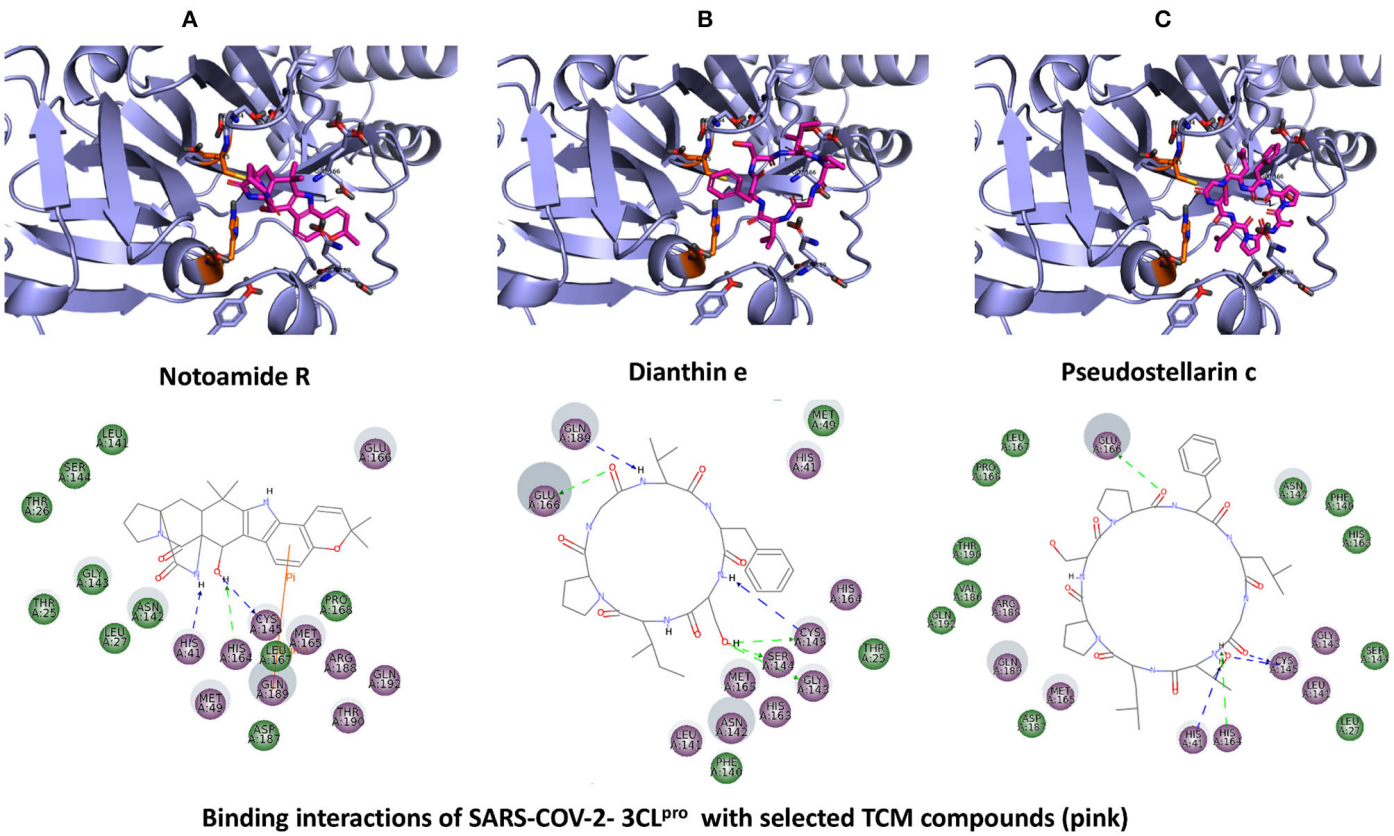
Binding interactions of severe acute respiratory syndrome coronavirus 2 (SARS-CoV-2) - 3CL^pro^ with selected TCM compounds (pink). The hydrogen bond interactions with protein backbone are indicated in blue dotted lines, and hydrogen bonds with the side-chain atoms are shown in green dotted lines. **(A)** Notoamide R. **(B)** Dianthin e. **(C)** Pseudostellarin c.

The top 3 compounds from the SuperTCM database are notoamide R, dianthin E, and pseudostellarin C. Notoamide R interacts with Cys145, His41, His164, and Gln189 (Figure 3A). Dianthin E interacts with the backbone atoms of Cys145 and Gln189, and the side-chain atoms of Glu166, Ser144, and Gly143 chiefly *via* hydrogen bonds (Figure 3B). Pseudostellarin C interacts with Cys145, His164, His41, and Glu166 (Figure 3C). Further information on the top 10 compounds from the SuperTCM database is reported in Supplementary Table 3.

**Figure 4:**
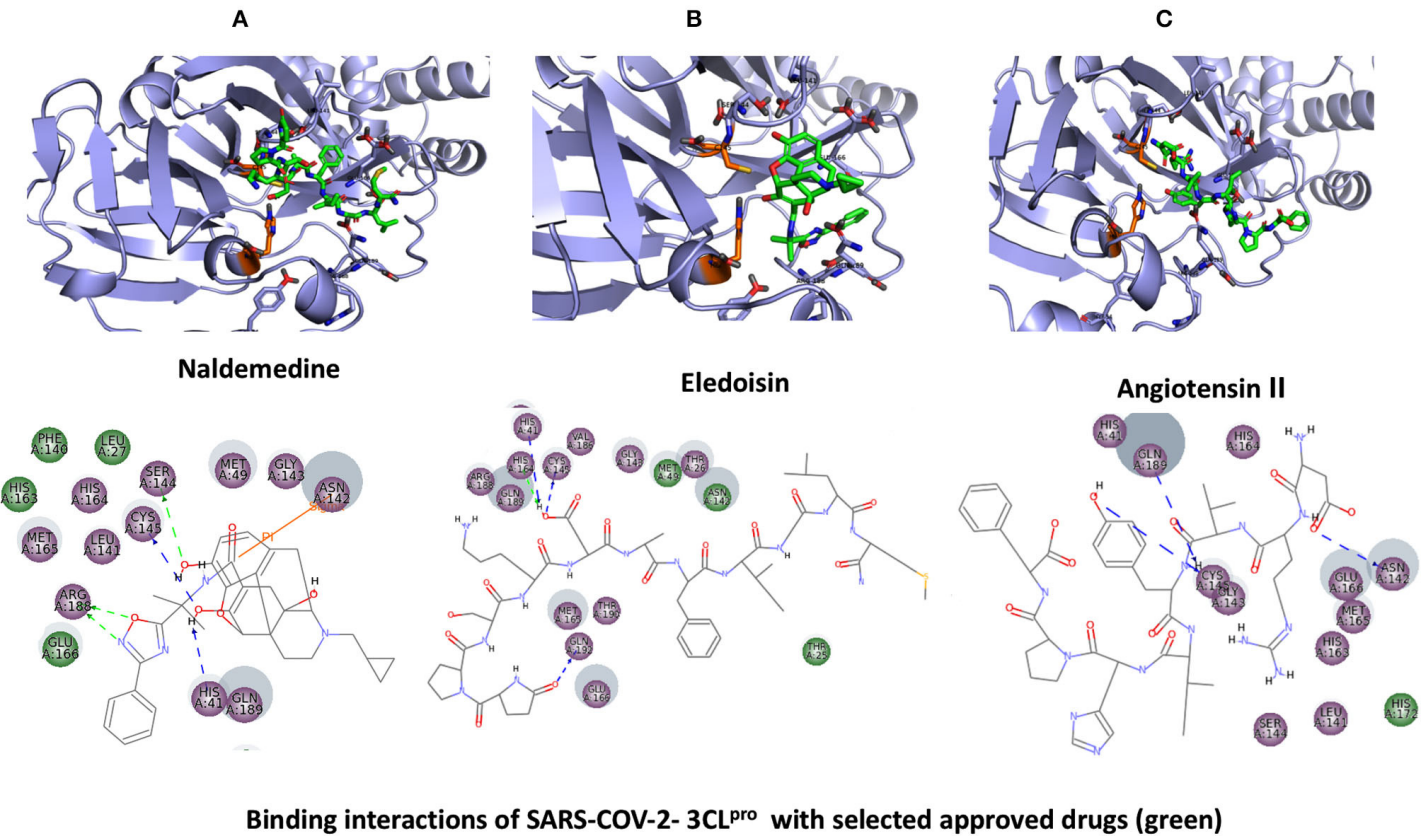
Binding interactions of severe acute respiratory syndrome coronavirus 2 (SARS-CoV-2) - 3CL^pro^ with selected approved drugs (green). The hydrogen bond interactions with protein backbone are indicated in blue dotted lines, and hydrogen bonds with the side-chain atoms are shown in green dotted lines. **(A)** Naldemedine. **(B)** Eledoisin. **(C)** Angiotensin II.

MD simulations do not suggest that Notoamide R engages in notable interactions with the binding site and, in fact, reveals an unstable pattern of protein–ligand contacts. Regarding the time evolution of the RMSDs and protein–ligand contacts, this ligand appears to adopt rather different conformations along the trajectory. During the first 30 ns, it occupies a stable conformation and nominally interacts with His41 and, to a lesser extent, with Cys145. A conformational change then occurs, and the positions adopted by the ligand from 30 to 100 ns preclude contacts with binding-site residues. Thus, this ligand is not considered as a suitable candidate for 3CL^pro^ inhibition (**Figure 7**).

The RMSD graphic of Dianthin E shows a highly stable pose along the first ≈40 ns. After that, a peak in the plot indicates a shift in the pose; nevertheless, the simulation concludes with stable RMSD values for protein and ligand (granted, these values are higher than for the other ligands simulated here). The patterns of protein–ligand contacts are in concordance with the RMSD fluctuations. The first ≈40 ns saw some interaction with Cys145 and, in a clearer way, with Glu166 and Gln189. The ligand reoriented in the next ≈60 ns, corresponding to a weak bond with His41 and to a stabilization of the Gln189 bond (**Figure 7**).

The RMSD trace for Pseudostellarin C shows a high stable pose from the first 40 ns. After that, there is a peak that indicates a pose change, but the simulation ends with stable RMSD values for protein and ligand. The protein–ligand contacts are aligned with the RMSD fluctuation. During the first 40 ns, there was some interaction with Cys145 and with higher interaction score values with Glu166 and Gln189 (Supplementary Figure 2). The ligand reorientation after the following 60 ns was translated to an intermittent bond with His41 and to a persistent bond with Gln189 (**Figure 7**).

### SuperDrug2 Drug Database

**Figure 5:**
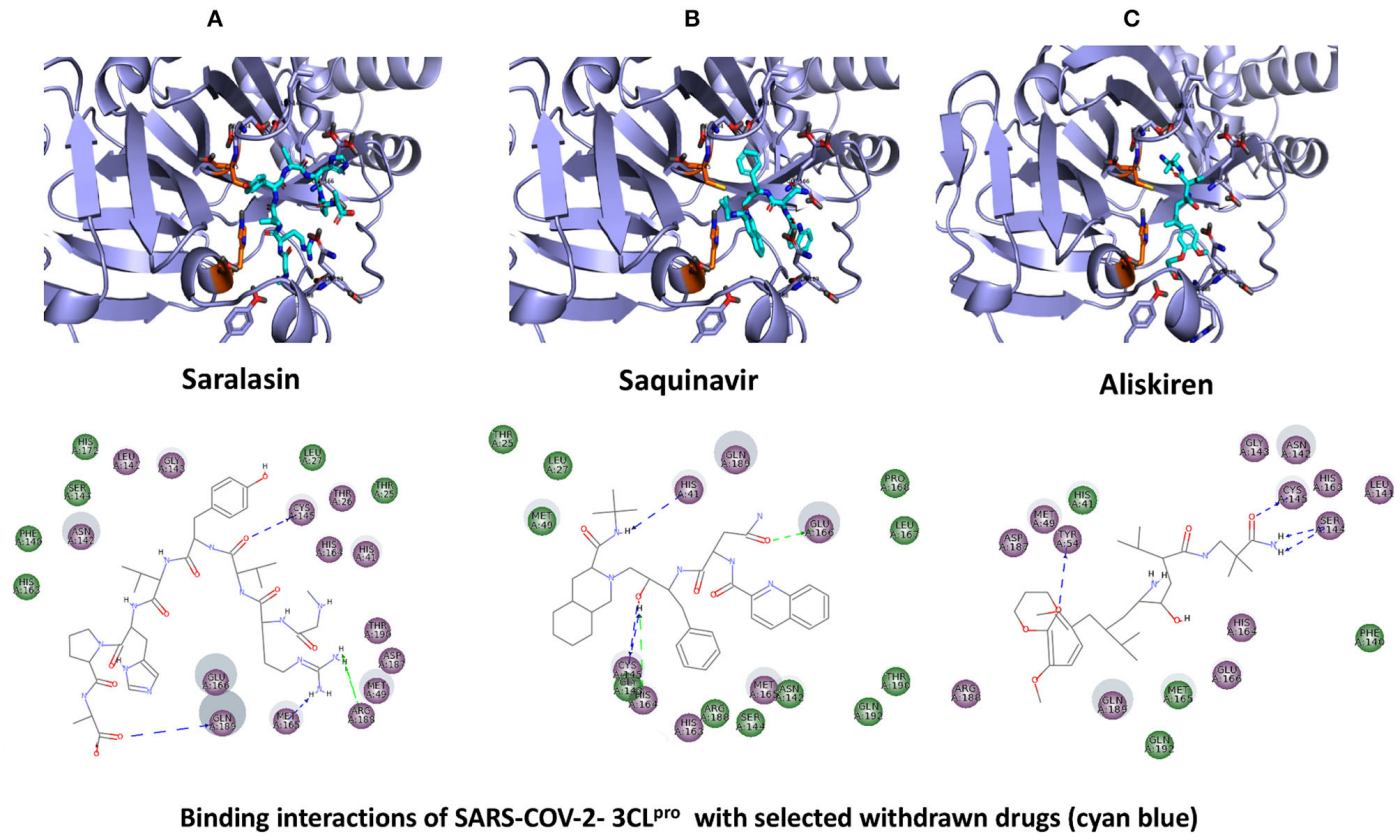
Binding interactions of severe acute respiratory syndrome coronavirus 2 (SARS-CoV-2) - 3CL^pro^ with selected withdrawn drugs (cyan blue). The hydrogen bond interactions with protein backbone are indicated in blue dotted lines, and hydrogen bonds with the side-chain atoms are shown in green dotted lines. **(A)** Saralasin. **(B)** Saquinavir. **(C)** Aliskiren.

The top three selected compounds from the approved drug database include naldemedine, eledoisin, and angiotensin II. Naldemedine interacts with Ser144, Arg188, and Asn142 (Figure 5A). It also interacts with the catalytic residues Cys145 and His41. Eledoisin also interacts with the catalytic residues His41 and Cys145 and with two other residues, Gln192 and His164 (Figure 5B). Angiotensin II interacts with Cys145, Gln189, and Asn142 (Figure 5C). More information on the 10 best drug candidates from the SuperDRUG2 database is reported in Supplementary Table 4.

Naldemedine shows close values for protein and ligand RMSDs, and the ligand pose is considered stable. Regarding protein–ligand contacts, the residues Glu166 and Gln189 were stable in contact throughout the simulation. Initially, also Cys145 showed a continuous interaction, so the S2 sub-pocket would be the main location responsible for 3CL^pro^ inhibition. After the first 60 ns, this interaction becomes less stable and the contacts with Glu166 and Gln189 become more prominent, being the S3–S5 subunits responsible for the inhibition. Hence, an inhibition mode that is initially dominated by interactions at the S2 sub-pocket (and a relatively minor S3–S5 presence) evolves over the course of the trajectory to feature dominant S3–S5 inhibition (**Figure 8**).

Eledoisin shows persistent interaction with Thr26. Also, it interacts with His41 and, to a lesser extent, with Cys145. This means that this ligand would efficiently bind the S2 subsite of 3CL^pro^. In addition, it interacts with Glu166 and Gln189. However, the glutamine contacts, despite being a strong (hydrogen bond) interaction, are relatively transient in the simulation; therefore, interactions at the S3–S5 site are considered less important than at the S2 site. Although the RMSD values are slightly higher than with saquinavir, we suspect that eledoisin could be a viable candidate to inhibit 3CL^pro^ (**Figure 8**).

The protein–ligand complex with angiotensin II exhibits somewhat elevated RMSD values. Angiotensin II reliably contacts the S1 subunit, being Gly143 and Glu166 responsible for the stronger and most stable interactions during the simulation. The S2 site was also the location of interactions with Thr26, but neither His41 nor Cys145 contacts were relevant. Furthermore, it interacts with the S3 and S5 sub-pockets by binding Glu166 and Gln189. The continuity and interaction scores of these contacts were remarkable during the whole simulation, so they are considered the most important key residues (**Figure 8**, Supplementary Figure 3).

### WITHDRAWN Drug Database

**Figure 6:**
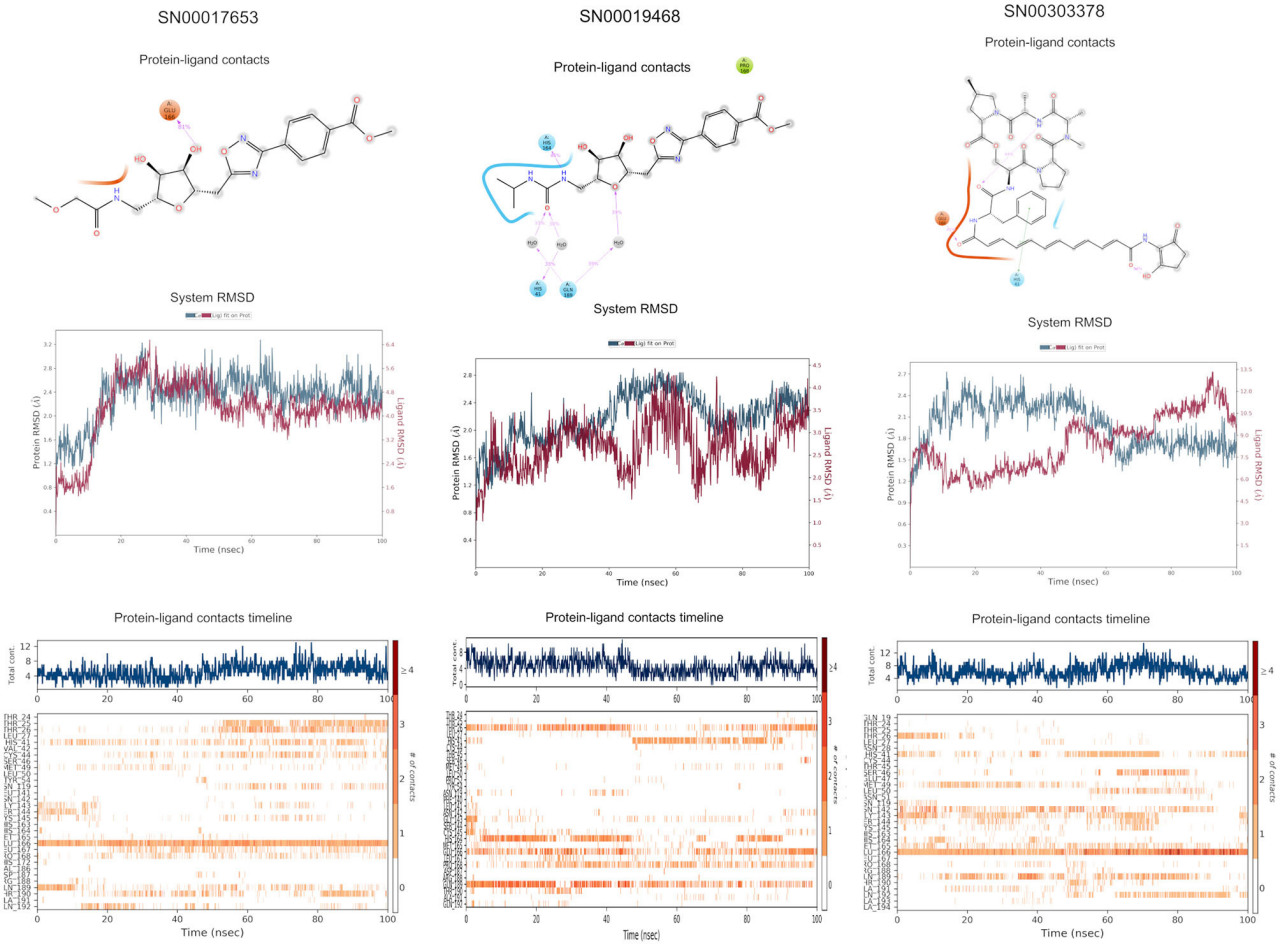
Molecular dynamics (MD) simulation studies of the severe acute respiratory syndrome coronavirus 2 (SARS-CoV-2) main protease with the predicted compounds from Super Natural II database.

The top 3 compounds from the WITHDRAWN database are saralasin, saquinavir, and aliskiren. Saralasin interacts with Cys145, Met165, Gln189, and Arg188 (Figure 6). Saquinavir interacts with Cys145 (backbone), Gly143, His41(backbone), and Glu166 (similar to original N3 ligand) (Figure 6). Aliskiren interacts with Tyr54, Cys145, and Ser144 (Figure 6). More information on the 10 potential drug candidates from the WITHDRAWN database is reported in Supplementary Table 5.

**Figure 7:**
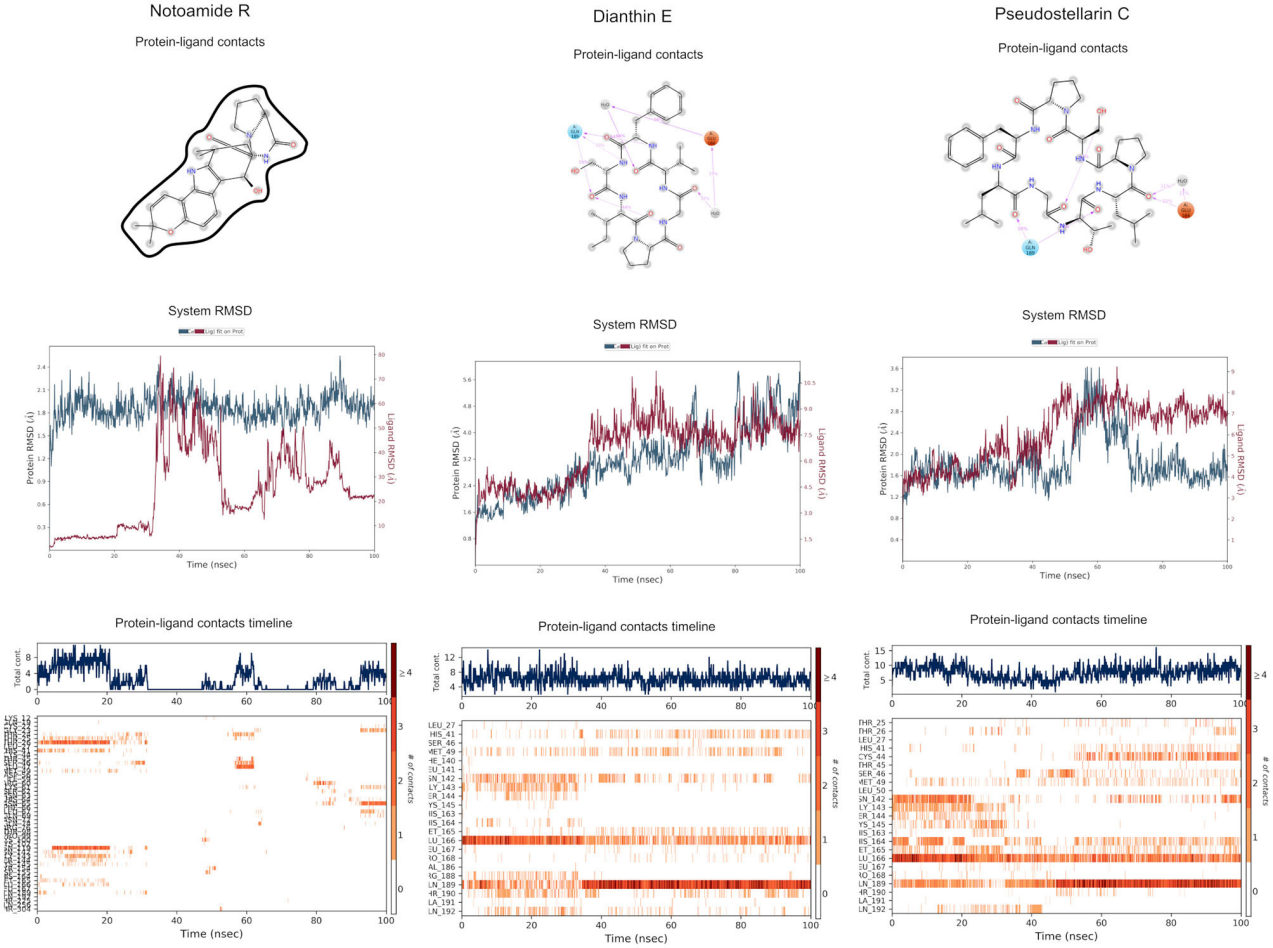
Molecular dynamics (MD) simulation studies of the severe acute respiratory syndrome coronavirus 2 (SARS-CoV-2) main protease with the predicted top 3 compounds from TCM database.

**Figure 8:**
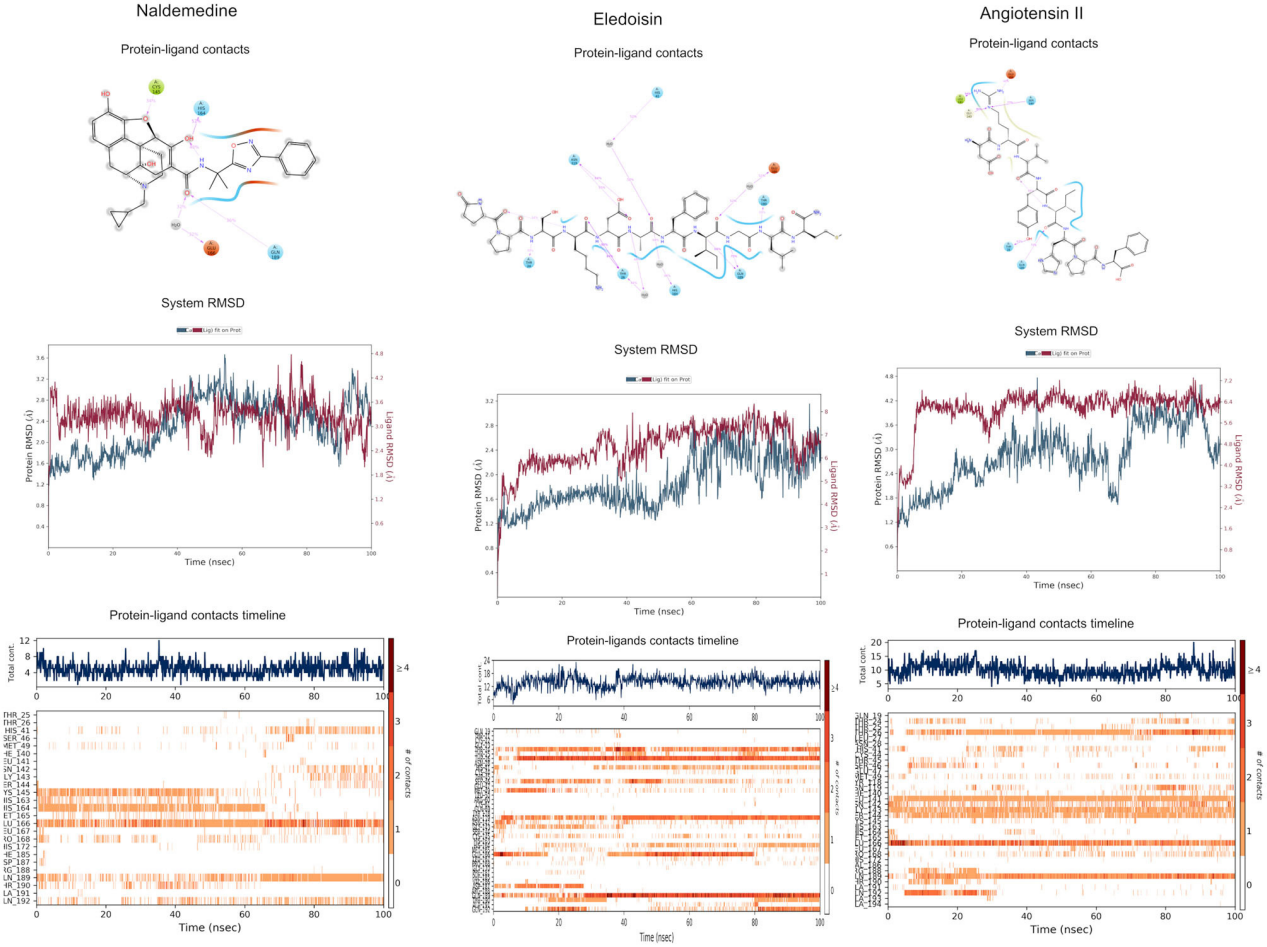
Molecular dynamics (MD) simulation studies of the severe acute respiratory syndrome coronavirus 2 (SARS-CoV-2) main protease with the predicted top 3 compounds from the approved drugs database (SuperDRUG2).

**Figure 9:**
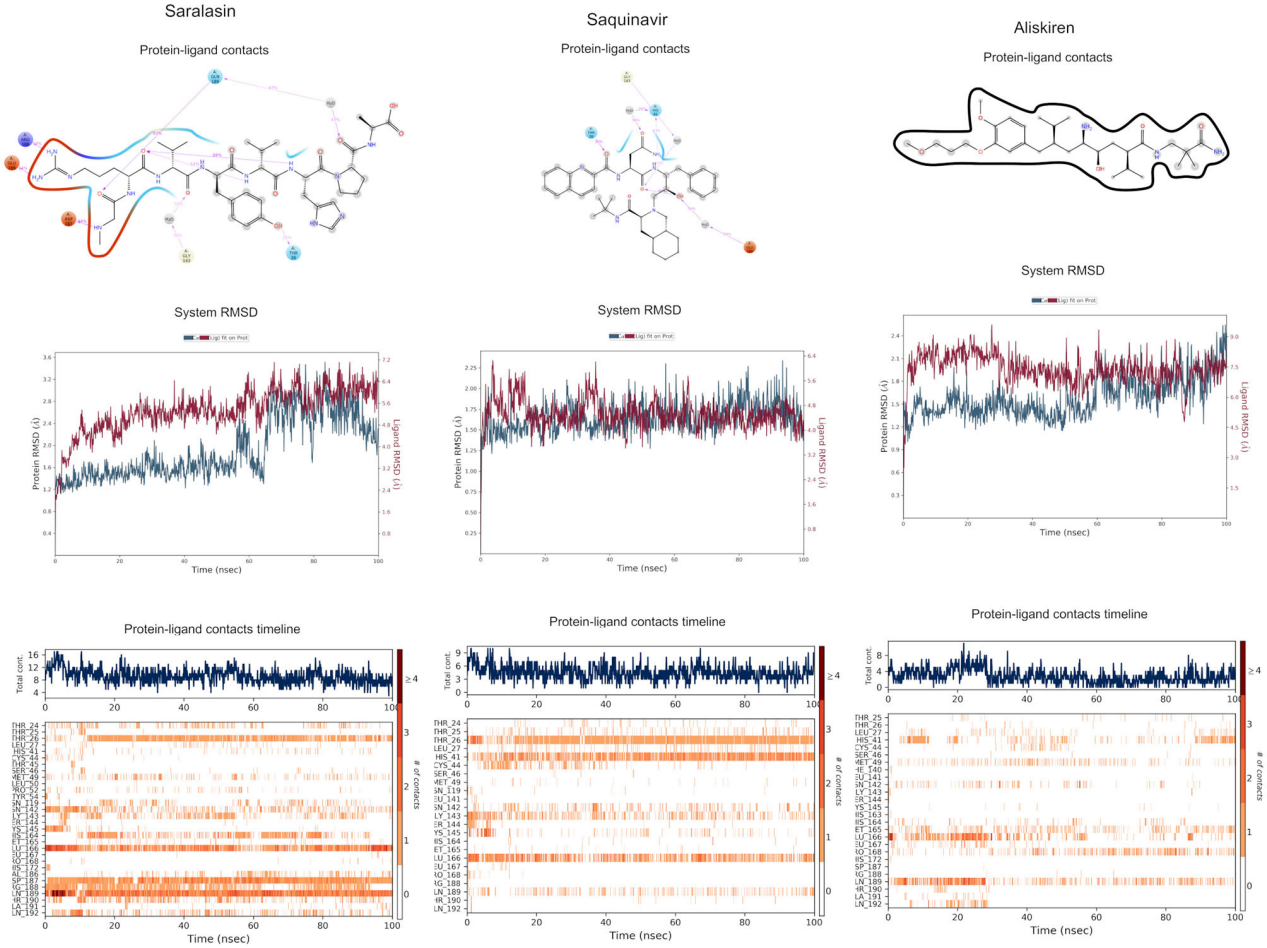
Molecular dynamics (MD) simulation studies of the severe acute respiratory syndrome coronavirus 2 (SARS-CoV-2) main protease with the predicted top 3 compounds from the WITHDRAWN database.

The protein–ligand complex with saralasin exhibits overall structural stability, although the RMSD values are slightly higher than for the other systems. Saralasin has no relevant interactions within the S1 sub-pocket, formed by Thr25, His41, and Cys145 (it interacts with Cys145 during the first 10 ns, but this key contact was lost thereafter). Saralasin does show clear, persistent interactions with the S3–S5 subunit, mediated by interactions with Glu166 and Gln189. The relatively low protein and ligand RMSD values for the saquinavir trajectory reflect the structural rigidity of this system, which maintains a stable conformation during the full 100-ns simulation. The ligand engages in energetically favorable contacts with His41 and Glu166 during the whole simulation. The role of these two key residues as primary positions of interaction/attachment could strongly anchor saquinavir to the protein, so this compound is considered a potential candidate to inhibit 3CL^pro^. The protein–ligand complex with aliskiren has low RMSD values. Nevertheless, despite maintaining the same conformation during the simulation, a sparse and transient pattern of interactions with protein residues would likely correspond to an unstable complex with aliskiren; this compound is not expected to be a good candidate for 3CL^pro^ inhibition (Figure 9, Supplementary Figure 4).

### Calculation of Relative Protein–Ligand Binding Free Energies Using the Molecular Mechanics–Generalized Born Surface Area Method

Notoamide R, which showed an inefficient performance to inhibit 3CL^pro^ during the MD analysis due to poor contacts with binding site residues and high instability, also showed poor MM-GBSA values, with −21.4 kcal/mol.

SN00017653, which is considered a strong inhibitor considering the RMSD analysis from MD results, shows a strong affinity, with −54.5 kcal/mol, and low deviation values. This MM-GBSA value is comparable to SN000303378, but this ligand, despite having a strong affinity value, does not interact with key binding site residues. SN00019468 shows also a strong affinity value, but this ligand does not interact significantly with the binding site residues and also has a high instability, which is translated to an elevated standard deviation value (16.5 kcal/mol), which is comparable to notoamide R; these two ligands are less suitable for inhibition of 3CL^pro^.

Both dianthin E and pseudostellarin C suffer a pose rearrangement during the 100-ns simulation, but the interaction within the binding site persists during the whole simulation despite of interacting with different residues. Thus, they were considered possible 3CL^pro^ inhibitors, and the MM-GBSA values indicate their strong affinity.

Aliskiren showed a stable conformation during the 100-ns simulation, but with intermittent contacts with binding site residues. MM-GBSA values show a strong interaction, but this is not relevant due to the discontinuous contacts.

Both saquinavir and saralasin showed a good performance during the MD analysis, and the MM-GBSA values also indicate a strong affinity of these two ligands within the binding site.

Eledoisin showed an important inhibitory potential during the MD analysis, and this is correlated to the high MM-GBSA affinity value, which shows the best score for all studied ligands (−93.3 kcal/mol).

Angiotensin II also showed persistent contacts with the main key residues of the binding site during the 100-ns simulation, and this is also extrapolated to the MM-GBSA values, which show a high-affinity energy for this ligand (−74.9 kcal/mol).

Naldemedine, which interacts within the key residues of the binding site, but has less persistent contacts than eledoisin and angiotensin II, also shows a lower MM-GBSA affinity (−64.2 kcal/mol) but comparable to the N3 ligand (−64.3 kcal/mol).

Excluding notoamide R, all these ligands showed good MM-GBSA affinity values in comparison to the previously identified N3 inhibitor. Nevertheless, the MD analysis showed poor interaction of SN00019468, SN000303378, and aliskiren within the binding site, so their corresponding MM-GBSA affinities are not considered relevant. Dianthin E, pseudostellarin C, saquinavir, saralasin, eledoisin, angiotensin II, and naldemedine are considered as potential 3CL^pro^ inhibitors, though eledoisin and angiotensin II are the most promising ones due to their lower MM-GBSA values (Table 4). The obtained results (Table 4) indicate the mean of the energy calculated for each pose of the simulation and its corresponding standard deviation.

## Clinical Insights

Our ultimate goal is to identify putative drug compounds that can be used safely and efficaciously while mitigating risks—deleterious side effects likely could not be reliably or robustly tolerated in severe COVID-19 cases. The general strategy of “drug repurposing” involves identifying existing compounds (both approved and withdrawn drugs) via their biological plausibility/rationale (e.g., mechanism-based inhibitors), via *in vitro, in vivo*, and *in silico* studies, or via serendipitous clinical observations. Much clinical pharmacological data, and clinical trial knowledge, are required in order to really elucidate (and extend) the use of a given chemical for a new indication; such efforts can stem from clinical expertise or smaller-scale studies (before a fully systematic, population-wide study). A consideration of the possible strengths and weaknesses of the drug candidates predicted herein will require expertise in translational drug development, including clinical pharmacologists and infectious disease specialists conducting clinical trials related to COVID-19. As an alternative to traditional drug development strategies, which are often slow, financially costly, and failure-prone, drug repositioning approaches, though computationally intricate, can be especially useful in emergency situations such as the COVID-19 pandemic. Identifying and selecting molecular candidates for drug repositioning entails numerous factors—e.g., pharmacokinetics, clinical indications, drug-related adverse events, drug–drug interactions, toxicity profiles, and available formulations.

With regard to pharmacokinetics, metabolism plays a key role in this selection. The Phase I metabolism, through the CYP450 family, significantly increases the risk of drug–drug interactions when coadministered with CYP inhibitors or inducers. Moreover, idiosyncratic genetic variability in the CYP gene family may affect an individual's response to the administered treatment, both in terms of effectiveness and tolerability. By these considerations, we suggest that eledoisin, daptomycin, and angiotensin II, which have no interaction with the CYP system (Supplementary Table 4), could be potential inhibitors of the SARS-CoV-2 main protease. However, the vasodilatory activity of eledoisin may render it unsuitable in critically ill patients because severe cases (e.g., septic shock) often require vasopressor support; in contrast, angiotensinamide, which is an oligopeptide used to increase blood pressure by vasoconstriction, could be an interesting option in septic patients. Drugs not specifically indicated for cardiovascular disease may also affect hemodynamics. For instance, dihydroergocornine, a dopamine agonist used as an anti-Parkinson agent, presents large hypotensive effects; therefore, it should not be considered as a potential medication for COVID-19-infected patients. A consensus seems to be emerging that cardiovascular drugs (or other medications with significant hemodynamic effects) should not be considered promising candidates for targeting SARS-CoV-2, as their overall efficacy (and potential side effects) is too coupled to other clinical aspects of a patient's condition.

There are also additional considerations in the drug selection process, such as the environments of use and how a patient will interact with the drug, and these factors may influence the chosen administration route. Most infected COVID-19 patients have been managed in non-critical areas; therefore, in this context, the oral route of administration can be considered feasible, alongside with intravenous injections. Naldemedine, which is an oral peripherally acting μ-opioid receptor antagonist (PAMORA), indicated for opioid-induced constipation (Coluzzi et al., 2020), may represent a practicable alternative in less severe COVID-19 patients. Its use, in healthy subjects, was associated with a slight increase in the incidence of diarrhea (Fukumura et al., 2018). Conversely, in critically ill patients, even when the enteral nutrition is guaranteed, oral formulations could be unsuitable if they cannot be crushed or dissolved and administered through the enteral feeding tube, as is the case with [CM1] naldemedine. From a clinical point of view, the approved drug indication could be a relevant criterion for selection, analogous to what holds true for antimicrobials and antiviral drugs. Indeed, the COVID-19 pandemic has seen remdesivir, an established drug with broad-spectrum antiviral activity, receive emergency use authorization from the FDA and the European Medicines Agency (EMA) (Grein et al., 2020). Similarly, the antiviral medication telaprevir, a hepatitis C virus protease inhibitor, could represent an alternative. Among antibacterial agents, daptomycin, which is a lipopeptide antibiotic with *in vitro* bactericidal activity against Gram-positive bacteria, could be interesting potential inhibitors for SARS-CoV-2 targets. Daptomycin remains one of the main treatment options for methicillin-resistant *Staphylococcus aureus* (MRSA) infections; however, sporadic cases of resistance have been noted (Barros et al., 2019). In drug repositioning approaches, even withdrawn drugs can become reborn—particularly if the reason for market withdrawal was commercial (i.e., not safety issues). The top three withdrawn compounds that we have identified here include two cardiovascular drugs and an antiviral. Saralasin, an old partial agonist of angiotensin II receptors, has been withdrawn from sale for commercial reasons. Similarly, aliskiren, a direct renin inhibitor—which failed to show benefit over angiotensin-converting-enzyme (ACE) inhibitors in heart failure^21^—was withdrawn from the European market, without intention to market it in the future. Saquinavir, indicated for treatment of HIV-1-infected adult patients, also could be an appealing potential drug for COVID-19 patients. However, apart from its withdrawal from the market, the CYP activity profile causes significant potential clinically relevant drug–drug interactions with a number of coadministered drugs. When considering the top 10 withdrawn compounds (Supplementary Table 5)—excluding cardiovascular drugs (for concerns expressed above) and antineoplastic agents (for reasons of toxicity)—we suggest that antibacterial drugs could be considered. Azlocillin, a wide-spectrum acylated form of ampicillin with antibacterial activity, has been recently proposed as a potential drug candidate for Lyme disease (targeting drug-tolerant *Borrelia burgdorferi*). Besides its efficacy, the safety of azlocillin was one of the main criteria for selecting this drug (Pothineni et al., 2020). In terms of drug repositioning, note that azlocillin has also been investigated as a potential new therapeutic agent for prostate cancer (Turanli et al., 2019). Among antibacterial agents, azlocillin does not present toxicity issues and has no interactions with the CYP family; therefore, it could be considered a first-choice molecule in this pharmacologic class of agents.

## Conclusions

This study reports potential inhibitors for the SARS-CoV-2 main protease, 3CL^pro^, *via* an integrated computational approach to drug repositioning. After our docking trials, a divergent pose of ligands was generated, and the pose with the optimal docking score and binding interactions was considered as the best pose for further processing and manual analysis. The docking of compounds to the 3CL^pro^ protease was visualized in terms of interactions in the substrate recognition pockets of the protein, and the dynamical stability of drug–protein contacts was evaluated *via* MD simulations of each putative drug−3CL^pro^ pair. We identified compounds from four different sources—namely, the Super Natural II, TCM, approved drugs, and WITHDRAWN drugs databases. Most of the compounds identified in our present work exhibit favorable interactions with the main protease residues (Cys145, Ser144, Glu166, His41, Gln189, and Gln192), suggesting that enthalpically optimal interactions at least *can* occur (see Results section). Our proposed compounds, as bound in the active site (pocket) of the 3CL^pro^ protein, are shown in Figures 2–5. The steric accommodation of selected compounds in 3CL^pro^ hinges upon particular amino acid residues that engage in interactions, as shown in Figures 6–9. Our analyses elucidate, at least *in silico*, these potential drug−3CL^pro^ interactions. Some drugs, like naldemedine and candidates from Super Natural II (SN00017653) and TCM (pseudostellarin C), have not been identified in previous studies as potential inhibitors of SARS-CoV-2 main protease and are suggested here for the first time. Interestingly, pseudostellarin C, which is a compound found in the roots of a traditional Chinese plant (*Pseudostellaria heterophylla*), is known medicinally for its application in dry cough arising from “lung dryness” (Hu et al., 2019).

Naturally occurring compounds are a rich resource for drug innovation and development. We suggest that the COVID-19-related leads reported here can support the discovery and development of high-potency inhibitors *in vitro* and *in vivo*. New leads from the Super Natural II databases (Figure 2, Table 2) are novel promising candidates, as they have similar binding interaction profiles [including overall good structural similarity (0.73 and above)] as compared to the main N3 inhibitors. Besides that, the compounds from the TCM database have also shown good interactions with the main protease (Figures 3, 7, Table 2). Specially, compound pseudostellarin C is a promising candidate from the TCM chemical space, sharing similar binding interactions as the N3 ligand and showing a high stable pose from the first 40 ns (Figure 7) on MD simulation studies.

Additionally, the repurposed drugs (as shown in Table 3, Figures 4, 5) have shown good interactions; in particular, naldemedine from the approved drug set, which is a PAMORA recently approved for the treatment of opioid-induced constipation in adult patients (Hu and Bridgeman, 2018). This drug is also supported as a clinically valid alternative due to its safe profile. Furthermore, based on the MD simulation studies, naldemedine shows close values for protein and ligand RMSD, so the ligand pose is considered stable. Another interesting candidate is saquinavir from the withdrawn dataset, which interacts with the main protease in a similar manner as compared to its original ligand (Figure 6). It has a structural similarity of 0.67 with the original N3 ligand. Saquinavir is an antiretroviral protease (peptidomimetic) inhibitor that is used in the therapy and prevention of human immunodeficiency virus (HIV) infection and the acquired immunodeficiency syndrome (AIDS). This drug is discontinued in Europe^10^, and due to its CYP activity profile, which causes significant potential clinically relevant drug–drug interactions with a number of coadministered drugs, this drug becomes clinically less preferable as a COVID-19 potential drug candidate. Saquinavir is also reported as a potential repurposed drug for COVID-19 disease by other studies (Montenegro et al., 2020). On the other hand, drugs such as angiotensin II, aliskiren, and phytochemicals like notoamide R, SN00019468, dianthin E, and SN00303378 cannot be considered as optimal candidates based on this study, as some of these compounds were stable but showed poor contacts with crucial residues of the main protease, as well as some showed higher RMSD values on MD simulation studies, and sometimes both poor contacts and high ligand RMSD fluctuations were observed (see Results section). Additionally, in this study, we have also addressed the toxicity and cytochrome activity of the reported compounds. Most of the resulting compounds predicted to be immunotoxic (that is, cytotoxicity of the B and T cells). Thus, we believe gaining insight into the molecular mechanism responsible for protein–ligand recognition through this study will facilitate the development of drugs for the treatment of COVID-19 disease.

The work reported here addresses an important concern and urgent need for drugs for the treatment of SARS-CoV-2 infection. As demonstrated via this integrated approach, computational prediction of approved drugs, withdrawn drugs, and phytochemicals for inhibition of SARS-CoV-2 main protease has resulted in some promising leads for further experimental validation. We hope that the *in silico* results and predictions obtained in this study, including the potential clinical insights, could facilitate the discovery of highly potent inhibitors of the SARS-CoV-2 main protease. Overall, our computational drug repositioning strategy predicts some promising drug candidates that, if borne out *via* experimental and clinical approaches, could contribute toward resolving the global crisis of the COVID-19 pandemic.

## Data Availability Statement

The raw data supporting the conclusions of this article will be made available by the authors, without undue reservation.

## Author Contributions

RP, MS, and PB conceived the project. RA, MP, HP-S, QC, FC, MR, RP, and PB designed the project workflow. RA, QC, MP, and PB prepared the databases. QC performed SuperTCM data curations. RA, MP, HP-S, and PB implemented the project. RA performed molecular docking. RA and PB analyzed and selected the compounds. MP and HP-S performed the molecular dynamics. FC, MP, PC, and MS contributed to the clinical insights and clinical trial study. RA, MP, and PB provided the figures. RA and QC provided the Supplementary Material. PB, RA, MP, FC, CM, and PB contributed to manuscript writing. All authors proofread the final manuscript.

## Conflict of Interest

The authors declare that the research was conducted in the absence of any commercial or financial relationships that could be construed as a potential conflict of interest. The reviewer KV declared a past co-authorship with one of the authors HP-S to the handling Editor.

## Footnotes

^1^https://www.who.int/health-topics/coronavirus#tab=tab_1 (accessed July 28, 2020).

^2^WHO Coronavirus Disease (COVID-19) Dashboard | WHO Coronavirus Disease (COVID-19) Dashboard. Available online at: https://covid19.who.int/?gclid=CjwKCAjwsO_4BRBBEiwAyagRTezBE06lkOlefHglbg0emog5Zo38YH6hOcVz3YyPcI5LkKi0DCIxwhoCkGgQAvD_BwE (accessed July 28, 2020).

^3^DrugBank. Available online at: https://www.drugbank.ca/covid-19 (accessed July 28, 2020).

^4^Draft landscape of COVID-19 candidate vaccines. Available online at: https://www.who.int/publications/m/item/draft-landscape-of-covid-19-candidate-vaccines (accessed July 28, 2020).

^5^Solidarity clinical trial for COVID-19 treatments. Available online at: https://www.who.int/emergencies/diseases/novel-coronavirus-2019/global-research-on-novel-coronavirus-2019-ncov/solidarity-clinical-trial-for-covid-19-treatments (accessed July 28, 2020).

^6^Coronavirus Disease 2019 (COVID-19) | CDC. Available online at: https://www.cdc.gov/coronavirus/2019-ncov/index.html (accessed July 30, 2020).

^7^Super Natural II–a database of natural products - PubMed. Available at: https://pubmed.ncbi.nlm.nih.gov/25300487/ (accessed July 28, 2020).

^8^ETCM: an encyclopaedia of traditional Chinese medicine. Available online at: https://pubmed.ncbi.nlm.nih.gov/30365030/ (accessed July 28, 2020).

^9^SuperDRUG2: a one stop resource for approved/marketed drugs. Available online at: https://pubmed.ncbi.nlm.nih.gov/29140469/ (accessed July 28, 2020).

^10^WITHDRAWN–a resource for withdrawn and discontinued drugs – PubMed. Available online at: https://pubmed.ncbi.nlm.nih.gov/26553801/ (accessed July 28, 2020).

^11^RCSB PDB: Homepage. Available online at: https://www.rcsb.org/ (accessed July 31, 2020).

^12^Structural Bioinformatics Group. Available online at: http://bioinf-apache.charite.de/main/index.php (accessed July 31, 2020).

^13^Traditional Chinese Medicine: Institut für Theorie, Geschichte, Ethik Chinesischer Lebenswissenschaften - Charité – Universitätsmedizin Berlin Available online at: https://icl.charite.de/forschung/buch_details_2011_2020/traditional_chinese_medicine (accessed July 31, 2020).

^14^KEGG PATHWAY Database. Available online at: https://www.genome.jp/kegg/pathway.html (accessed July 31, 2020).

^15^RDKit. Available online at: https://www.rdkit.org/ (accessed July 31, 2020).

^16^Free Download: BIOVIA Discovery Studio Visualizer - Dassault Systèmes. Available online at: https://discover.3ds.com/discovery-studio-visualizer-download (accessed July 31, 2020).

^17^PyMOL | pymol.org. Available online at: https://pymol.org/2/ (accessed July 31, 2020).

^18^Maestro | Schrödinger. Available online at: https://www.schrodinger.com/maestro (accessed July 31, 2020).

^19^ProTox-II: a webserver for the prediction of toxicity of chemicals. Available online at: https://pubmed.ncbi.nlm.nih.gov/29718510/ (accessed July 28, 2020).

^20^SuperCYPsPred—a web server for the prediction of cytochrome activity. Available online at: https://academic.oup.com/nar/article/48/W1/W580/5809167 (accessed July 28, 2020).

^21^End of the road for Aliskiren in heart failure. Available online at: https://academic.oup.com/eurheartj/article/38/5/312/2990021 (accessed July 28, 2020).

## Abbreviations

SARS-CoV-2: severe acute respiratory syndrome coronavirus 2
WHO: World Health Organization
COVID-19: coronavirus disease 2019
FDA: Food & Drug Administration
EMA: European Medicines Agency
TCM: Traditional Chinese Medicine
MD: molecular dynamics
VS: virtual screening
2D: two-dimensional
3D: three-dimensional
LBVS: ligand-based virtual screening
SBVS: structure-based virtual screening
ADMET: absorption, distribution, metabolism, elimination, and toxicity
LD: lethal dose
RMSD: root-mean-square deviation
CYP: cytochrome P450
ACE: angiotensin-converting-enzyme.
