# Supplementary material for: Computational Prediction of Potential Inhibitors of the Main Protease of SARS-CoV-2
Source: Front Chem. 2020 Dec 23;8:590263. doi: 10.3389/fchem.2020.590263 (PMC7786237; doi:10.3389/fchem.2020.590263)

***Supplementary Material***

**Supplementary material** contains tables and figures:

**Supplementary Table 1 -** with fingerprints comparison, based on approved drugs dataset and 3 types of fingerprints (MACCS, ECFP-4, E-State).

**Supplementary Table 2 -** with 10 potential inhibitors of SARS-CoV-2 main protease from Super Natural II database with additional information on interacting residues, predicted toxicity and predicted CYP activity.

**Supplementary Table 3-** with 10 potential inhibitors of SARS-CoV-2 main protease from Traditional Chinese Medicine (SuperTCM ) with additional information on interacting residues, predicted toxicity and predicted CYP activity.

**Supplementary Table 4 –** with 10 potential inhibitors of SARS-CoV-2 main protease from SuperDrug2 database with additional information on interacting residues, predicted toxicity, predicted CYP activity and therapeutic endpoints.

**Supplementary Table 5 -** with 10 potential inhibitors of SARS-CoV-2 main protease from Withdrawn database with additional information on interacting residues, predicted toxicity and predicted CYP activity and therapeutic endpoints.

**Supplementary Table 6 -** with 10 potential inhibitors of SARS-CoV-2 main protease from Traditional Chinese Medicine (SuperTCM) and additional information on their respective TCM plants and indications.

**Supplementary Table 7 -** with 40 compounds (based on the highest Tanimoto score) from screening of potential of SARS-CoV-2 main protease 3CL inhibitors for a reference molecule N3 from PDB 6LU7. Results are divided into 4 categories: Approved (drugs from SuperDrug2 database), Withdrawn (drugs WITHDRAWN database), SuperTCM (Traditional Chinese Medicine compounds), Super Natural II (natural products from Super Natural II database).

**Supplementary Table 8 -** with 40 compounds (based on the highest Tanimoto score) from screening of potential of SARS-CoV-2 main protease 3CL inhibitors for a reference molecule 6OK from PDB 6Y2F. Results are divided into 4 categories: Approved drugs (drugs from SuperDrug2 database), Withdrawn drugs (drugs WITHDRAWN database), SuperTCM (Traditional Chinese Medicine compounds), Super Natural II compounds (natural products database).

**Supplementary Table 9 -** includes images of two-dimensional protein-ligand binding interactions from molecular docking studies. Compounds presented in Supplementary Table 7 and Supplementary Table 8 were docked to the crystal structure of 3CL^pro^ of SARS-CoV-2 (PDB 6LU7). In total 76 compounds were docked (as four compounds were common in the hit list of the databases). Eight compounds out of this screened 76 compounds did not show any ligand-protein interaction and so was excluded from the analysis. 12 best results were presented in the main manuscript as best candidates. The additional 56 compounds are presented in this table.

**Supplementary Table 10** – with GOLD docking scores (GoldScore fitness) for 12 chosen compounds and re-docked 3CLpro ligands (N3 and O6K).

**Supplementary Figure 1 -** protein-ligand contacts diagram (presenting intention type and score)

for 3 chosen compounds from Super Natural II database.

**Supplementary Figure 2 -** protein-ligand contacts diagram (presenting intention type and score)

for 3 chosen compounds from Traditional Chinese Medicine database (SuperTCM).

**Supplementary Figure 3** protein-ligand contacts diagram (presenting intention type and score)

for 3 chosen compounds from SuperDrug2 database.

**Supplementary Figure 4 -** protein-ligand contacts diagram (presenting intention type and score)

for 3 chosen compounds from WITHDRAWN database.

**Supplementary Figure 5 –** protein-ligand contacts diagram for N3 ligand.

**Supplementary Figure 6 -** MD simulation studies of the SARS-CoV-2 main protease with the N3 ligand.

**Table 1: Fingerprints comparison**

| **APPROVED DRUS** | **6OK inhibitor** |  | **APOPROVED DRUGS** | **N3 inhibitor** |
| --- | --- | --- | --- | --- |
| MACCS | Tanimoto score |  | MACCS | Tanimoto score |
| Lopinavir | 0.83 |  | Elcatonin | 0.74 |
| Angiotensinamide | 0.82 |  | Secretin Porcine | 0.74 |
| Indinavir | 0.82 |  | Daptomycin | 0.74 |
| Dihydroergocristine | 0.81 |  | Enfuvirtide | 0.73 |
| Dihydroergocornine | 0.81 |  | Albiglutide | 0.73 |
| Dihydroergocryptine | 0.81 |  | Secretin Human | 0.73 |
| Epicriptine | 0.80 |  | Angiotensin II | 0.73 |
| Bivalirudin | 0.80 |  | Naldemedine | 0.72 |
| Angiotensin II | 0.80 |  | Angiotensinamide | 0.72 |
| Dihydroergotamine | 0.79 |  | Eledoisin | 0.72 |
| **ECFP4** |  |  | **ECFP4** |  |
| Lopinavir | 0.28 |  | Secretin Human | 0.30 |
| Remikiren | 0.27 |  | Secretin Porcine | 0.30 |
| Buramate | 0.27 |  | Ubenimex | 0.29 |
| Lacosamide | 0.26 |  | Carfilzomib | 0.28 |
| Saquinavir | 0.25 |  | Bortezomib | 0.28 |
| Nateglinide | 0.24 |  | Sermorelin | 0.28 |
| Pivampicillin | 0.24 |  | Angiotensinamide | 0.27 |
| Alacepril | 0.24 |  | Glucagon | 0.27 |
| Pivhydrazine | 0.24 |  | Albiglutide | 0.27 |
| Atazanavir | 0.23 |  | Thymalfasin | 0.26 |
| **E-state** |  |  | **E-state** |  |
| Bitolterol | 0.92 |  | Dalfopristin | 0.87 |
| Levobunolol | 0.92 |  | Capsaicin | 0.85 |
| Carteolol | 0.92 |  | Zucapsaicin | 0.85 |
| Dipivefrine | 0.92 |  | Ixabepilone | 0.80 |
| Diprafenone | 0.92 |  | Lusutrombopag | 0.80 |
| Docetaxel | 0.92 |  | Naldemedine | 0.80 |
| Paclitaxel | 0.92 |  | Befunolol | 0.79 |
| Talinolol | 0.92 |  | Enocitabine | 0.79 |
| Tobuterol | 0.92 |  | Meproscillarin | 0.79 |
| Cetamolol | 0.92 |  | Proscillaridin | 0.79 |

**Table 2: Top 10 compounds screened from the Super Natural II database**

| **Compounds** | **Interacting residues (3CL)** | **Acute Toxicity** | **Toxicity endpoints** | **CYP activity** |
| --- | --- | --- | --- | --- |
| N3-inhibitor | Glu166, Cys145, Gly143 | class 5 | NA | CYP3A4 |
| SN00017653 | Cys145, Gly143, Glu166, Ser144, Leu141, Thr26 | class 4 | NA | NA |
| SN00019468 | His41, Cys145, Gly143, Ser144, Leu141 | class 4 | NA | NA |
| SN00303378 | His41, Thr190, Gln192, Ser144 | class 3 | Immunotoxic | CYP3A4 |
| SN00314990 | Cys145, Asn142, Ser46, Thr24, His41, Gly143 | class 3 | Immunotoxic | NA |
| SN00254530 | Cys145, His164, His41 | class 4 | Immunotoxic | NA |
| O6K -inhibitor | Glu166, Gly143, Cys145, Ser144 | class 4 | NA | 3A4 |
| SN00012917 | Cys145, Thr26, His163 | class 4 | NA | NA |
| SN00107903 | Gly143, Cys145, Thr26 | class 5 | NA | 3A4, 2C9 |
| SN00101324 | Gln189, Glu166, Cys145, Gly143 | class 4 | NA | 3A4, 2D6 |
| SN00101312 | Cys145, Thr26, Glu166 | class 4 | NA | 3A4, 2D6 |
| SN00077453 | Glu166, Cys144, Leu27, Gln192 | class 4 | Immunotoxic | 3A4 |

**Table 3: Top 10 screened compounds from Traditional Chinese Medicine database (SuperTCM)**

| Traditional Chinese Medicine | Interacting residues (3CL) | Acute Toxicity | Toxicity endpoints | CYP activity |
| --- | --- | --- | --- | --- |
| N3-inhibitor | Glu166, Cys145, Gly143 | class 5 | None | 3A4 |
| Notoamide R | Cys145, His41, His164, Gln189 | class 4 | Immunotoxic | 3A4 |
| Dianthin E | Cys145, Gln189, Glu166, Ser144, Gly143 | class 4 | None | None |
| Pseudostellarin C | Cys145, His164, His41, Glu166 | class 4 | Immunotoxic | None |
| Nummularine A | Cys145 | class 3 | None | 3A4 |
| Jubanine A | Cys145 | class 3 | Immunotoxic | 3A4 |
| O6K-Inhibitor | Glu166, Gly143, Cys145, Ser144 | class 4 | None | 3A4 |
| Hirudin | His41, Cys145, Tyr54, Gln189, Asp48 | class 4 | None | None |
| Celogenamide A | Gln192, Glu166, Arg188, Ser144 | class 4 | Immunotoxic | 3A4 |
| Liciumin C Methyl | His41, Gln192, Cys145, Thr190 | class 4 | None | 3A4 |
| Segetalin E | His41, Tyr54, Cys145, Gln192 | class 4 | Immunotoxic | 3A4 |
| Notoamide O | Cys145 | class 4 | Immunotoxic | None |

**Table 4: Top 10 repurposed compounds from SuperDrug2 drugs database**

| Approved drugs | Interacting residues (3CL) | Therapeutic endpoints | Acute  Toxicity | Toxicity endpoints | CYP activity profile |
| --- | --- | --- | --- | --- | --- |
| N3-inhibitor | Glu166, Cys145, Gly143 | - | class 5 | None | 3A4 |
| Eledoisin | Ser144, Arg188, Asn142, Cys145, His41 | vasodilator | class 5 | None | None |
| Naldemedine | His41, Cys145, Gln192, His164 | ‎ Alimentary tract and metabolism | class 4 | Immunotoxic | 2D6 |
| Angiotensin II | Cys145, Gln189, Asn142 | Cardiac therapy | class 5 | None | None |
| Daptomycin | Gly143, Glu166, Cys145, Leu141, Ser144, Arg188 | Antibacterial | class 4 | Immunotoxic | None |
| Elcatonine | Tyr54, His41, Leu27, Leu141, Cys145 | Calcium homeostasis | class 4 | None | None |
| O6K- inhibitor | Glu166, Gly143, Cys145, Ser144 | - | class 4 | None | 3A4 |
| Bivalirudin | Gln189, Asn142, Pro168 | Antithrombotic agent | class 5 | None | None |
| Angiotensinamide | Cys145, Glu166, Met165, Arg188 | Cardiovascular system | class 5 | None | 11B1, 11B2, 21A |
| Dihydroergocornine | Gln189, Cys145 | Anti-Parkinson drugs | class 5 | None | 3A4, 2C19, 2C9 |
| Telaprevir | Gln189, Cys145 | Antiviral | class 4 | None | 3A4 |
| Indinavir | His41 | Antiviral | class 5 | Hepatotoxic, Immunotoxic | 3A4, 2C19, 2C9, 2D6 |

**Table 5: Top 10 repurposed compounds from Withdrawn drugs database**

| Withdrawn drugs | Interacting residues (3CL) | Therapeutic endpoints | Acute  Toxicity | Toxicity endpoints | CYP activity profile |
| --- | --- | --- | --- | --- | --- |
| N3-inhibitor | Glu166. Cys145. Gly143 |  | class 5 | None | 3A4 |
| Saralasin | Cys145, Met165, Gln189, Arg188 | Cardiac therapy | class 5 | None | None |
| Saquinavir | Cys145, Gly143, His41 and Glu166 | Antiviral | class 4 | None | 2C8, 2C9, 2C19, 2D6, 3A4, 3A5 |
| Aliskiren | Tyr54, Cys145, Ser144 | Cardiac therapy | class 5 | Immunotoxic | 3A4 |
| Abarelix | Glu166, Arg40, Ser139, Cys145 | Antineoplastic agents | class 4 | Immunotoxic | 3A4 |
| Topotecan | Glu166, Gln189 | Antineoplastic agents | class 3 | Phosphoprotein (Tumor Suppressor) p53, Cytotoxic, Immunotoxic | 3A4 |
| O6K-inhibitor | Glu166, Gly143, Cys145, Ser144 |  | class 4 | None | 3A4 |
| Alatrofloxacin | Gln192, Gln189, Thr26 | Antibacterial | class 5 | Hepatotoxic | None |
| Lypressin | Cys145, His41, Thr26 | Vasopressin and analogues | class 2 | None | 2D6, 3A4 |
| Azlocilin | Leu141, Cys145 | Antibacterial | class 5 | None | None |
| Telithromycin | Cys145, Glu166 | Antibacterial | class 3 | Hepatotoxic, Immunotoxic | 1A2, 2D6, 3A4, 3A7 |
| Pentagastrin | His164, Ser144, Cys145 | Diagnostic agent | class 3 | None | 3A4 |

**Table 6: Top 10 screened compounds from Traditional Chinese Medicine database and additional information on their respective TCM herbs**

| **Compound**  **name** | **Botanical**  **name** | **Chinese pharmaceutical**  **name** | **Pharmaceutical latin name** | **Indications *** | **Effects*** |
| --- | --- | --- | --- | --- | --- |
| **Segetalin E** | Gypsophila vaccaria (L.) Sm. | 王不留行 | Vaccariae Semen | Galactostasis, Amenorrhea, Dysmenorrhea, mammary welling abscess. | To quicken blood and free menstruation, promote lactation and disperse edema |
| **Pseudostellarin C** | Pseudostellaria heterophylla (Miq.) Pax | 太子参 | Pseudostellariae Radix | Spleen vacuity and fatigued body, Inappetence, weakness during convalescence, qi and yin vacuity, thirst due to spontaneous sweating. dry cough due to lung dryness. | To boost qi and fortify spleen, moisten lung and engender liquid. |
| **Celogenamide A** | Celosia argentea L. | 青葙 | Celosiae Semen | Red eyes with gall, eye screen, dizziness and dim vision, hypertension, nosebleed(epistaxis), wind-heat itching of skin, sore and lichen. | To dispel wind-heat, clear liver fire, brighten eyes and eliminate eye screens. |
| **Hirudin** | Gardenia jasminoides J.Ellis | 水栀 | Gardeniae Grandiflorae Fructus | Heat toxin, jaundice, nosebleed(epistaxis), nephritis with edema, sprain and contusion. | To clear heat and resolve toxin, disperse swelling and relieve pain. |
| **Dianthin E** | Dianthus superbus L. | 瞿麦 | Dianthi Herba | Strangury, heat strangury, blood strangury, stone strangury, urinary tract infection, urinary stoppage, dripping with inhibited pain, amenorrhea, carcinoma of esophagus, carcinoma of rectum. | To disinhibit urine and free strangury, break blood and free menstruation, anticancer. |
| **Notoamide R** | Euphorbia marginata Pursh | 银边翠 | NA | Irregular menstrual periods, injuries caused by falls, unknown pyogenic infections. | To promote blood flow for regulating menstruation, detumescence and drawing out poison. |
| **Notoamide O** | Euphorbia marginata Pursh | 银边翠 | NA | Irregular menstrual periods, injuries caused by falls, unknown pyogenic infections. | To promote blood flow for regulating menstruation, detumescence and drawing out poison. |
| **Nummularine A** | Ziziphus jujuba Mill. | 大枣 | Jujubae Fructus | Reduced food intake due to spleen vacuity, lack of strength and sloppy stool, visceral agitation. | To supplement center and boost qi, nourish blood and quiet spirit. |
| **Jubanine A** | Ziziphus jujuba Mill. | 大枣 | Jujubae Fructus | Reduced food intake due to spleen vacuity, lack of strength and sloppy stool, visceral agitation. | To supplement center and boost qi, nourish blood and quiet spirit. |
| **Lyciumin C** | Lycium chinense Mill. | 枸杞子 | Lycii Fructus | Lassitude in lumbus and knees. Emission, dizziness and dim vision, lung disease with cough, diabetes mellitus. | To nourish liver, brighten eyes, enrich kidney, moisten lung, lower blood pressure. |
| **Lyciumin C** | Lycium barbarum L. | 宁夏枸杞根皮 | Lycii Radix Cortex | Diabetes mellitus. malaria. tidal fever with night sweat, child gan accumulation with fever, cough and hemoptysis or dyspnea, blood ejection, spontaneous external bleeding. | To eliminate heat in blood, lower vacuity heat. |

*Source: Encyclopedia of Traditional Chinese Medicines - Molecular Structures, Pharmacological Activities, Natural Sources and Applications, Zhou, Jiaju, Xie, Guirong, Yan, Xinjian, 2011

**Table 7: Top 40 compounds - potential of SARS-CoV-2 main protease 3CL inhibitors / reference molecule - N3 inhibitor**

|  | **APPROVED DRUGS** |  | **WITHDRAWN DRUGS** |  |
| --- | --- | --- | --- | --- |
| **Reference** | **Drug name** | **Tanimoto** | **Drug name** | **Tanimoto** |
| N3 | Elcatonin | 0.74 | Saralasin | 0.71 |
| N3 | Secretin porcine | 0.74 | Romidepsin | 0.71 |
| N3 | Daptomycin | 0.74 | Abarelix | 0.69 |
| N3 | Enfuvirtide | 0.73 | Saquinavir | 0.67 |
| N3 | Albiglutide | 0.73 | Trabectedin | 0.67 |
| N3 | Secretin human | 0.73 | Topotecan | 0.66 |
| N3 | Angiotensin II | 0.73 | Pentagastrin | 0.65 |
| N3 | Naldemedine | 0.72 | Rescinnamine | 0.65 |
| N3 | Angiotensinamide | 0.72 | Dirithromycin | 0.64 |
| N3 | Eledoisin | 0.72 | Aliskiren | 0.63 |
|  | **TCM** |  | **SUPER NATURAL II** |  |
| **Tanimoto** | **Compound name** | **Tanimoto** | **Compound SN ID** | **Tanimoto** |
| N3 | Pseudostellarin C | 0.77 | SN00308384 | 0.76 |
| N3 | Nummularine A | 0.76 | SN00017653 | 0.75 |
| N3 | Notoamide G | 0.76 | SN00227324 | 0.74 |
| N3 | Segetalin D | 0.76 | SN00270610 | 0.74 |
| N3 | Jubanine A | 0.75 | SN00354661 | 0.74 |
| N3 | Notoamide R | 0.75 | SN00254530 | 0.74 |
| N3 | Mauritine A | 0.75 | SN00314990 | 0.74 |
| N3 | Dianthin E | 0.74 | SN00019468 | 0.74 |
| N3 | Segetalin E | 0.74 | SN00303378 | 0.73 |
| N3 | Mauritine B | 0.74 | SN00238988 | 0.73 |

**Table 8: Top 40 compounds - potential of SARS-CoV-2 main protease 3CL inhibitors / reference molecule – O6K inhibitor**

|  | **APPROVED DRUGS** |  | **WITHDRAWN DRUGS** |  |
| --- | --- | --- | --- | --- |
| **Reference** | **Drug name** | **Tanimoto** | **Drug name** | **Tanimoto** |
| O6K | Lopinavir | 0.83 | Saralasin | 0.78 |
| O6K | Angiotensinamide | 0.82 | Saquinavir | 0.77 |
| O6K | Indinavir | 0.82 | Trabectedin | 0.74 |
| O6K | Dihydroergocristine | 0.81 | Tofacitinib Citrate | 0.73 |
| O6K | Dihydroergocornine | 0.81 | Lypressin | 0.73 |
| O6K | Dihydroergocryptine | 0.81 | Alatrofloxacin | 0.73 |
| O6K | Epicriptine | 0.80 | Nelfinavir | 0.73 |
| O6K | Bivalirudin | 0.80 | Pentagastrin | 0.72 |
| O6K | Dihydroergotamine | 0.79 | Azlocillin | 0.72 |
| O6K | Telaprevir | 0.79 | Telithromycin | 0.71 |
|  | **TCM** |  | **SUPER NATURAL II** |  |
| **Tanimoto** | **Compound name** | **Tanimoto** | **Compound SN ID** | **Tanimoto** |
| O6K | Hirudin | 0.82 | SN00109804 | 0.83 |
| O6K | Segetalin E | 0.80 | SN00087725 | 0.82 |
| O6K | Lyciumin C | 0.80 | SN00077453 | 0.82 |
| O6K | Celogenamide A | 0.80 | SN00077454 | 0.82 |
| O6K | Notoamide O | 0.80 | SN00101312 | 0.82 |
| O6K | Ergosine | 0.79 | SN00108593 | 0.82 |
| O6K | Ergosinine | 0.79 | SN00107903 | 0.8 |
| O6K | Ergocornine | 0.79 | SN00213824 | 0.8 |
| O6K | Ergocorninine | 0.79 | SN00012917 | 0.79 |
| O6K | Ergocryptine | 0.79 | SN00101324 | 0.79 |

| **Table 9:** Binding interactions of SARS-CoV-2 main protease 3CL^pro^ and docked 56 compounds | | |
| --- | --- | --- |
| NR | COMPOUND NAME | APPROVED DRUGS / REFERENCE MOLECULE: N3 |
| 1 | Elcatonin | 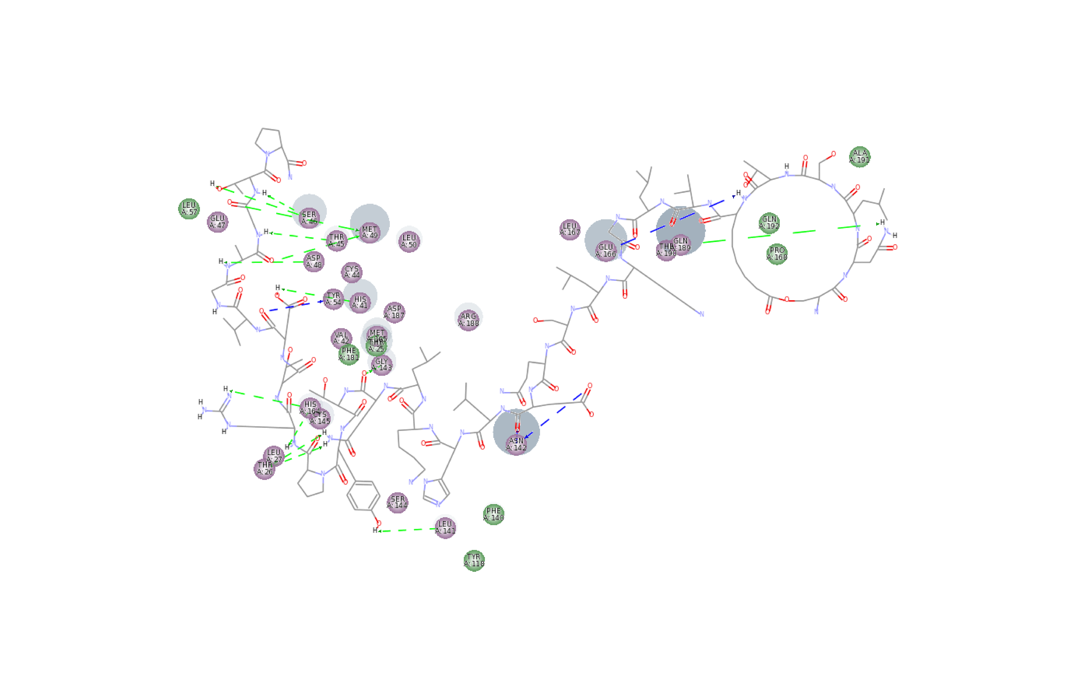 |
| 2 | Secretin porcine | 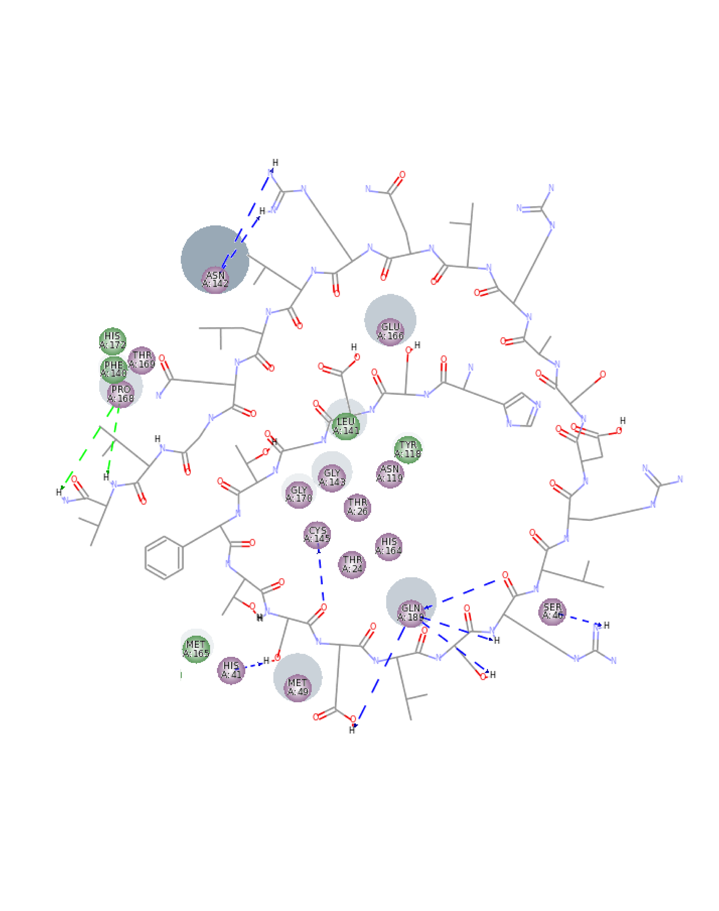 |
| 3 | Daptomycin | 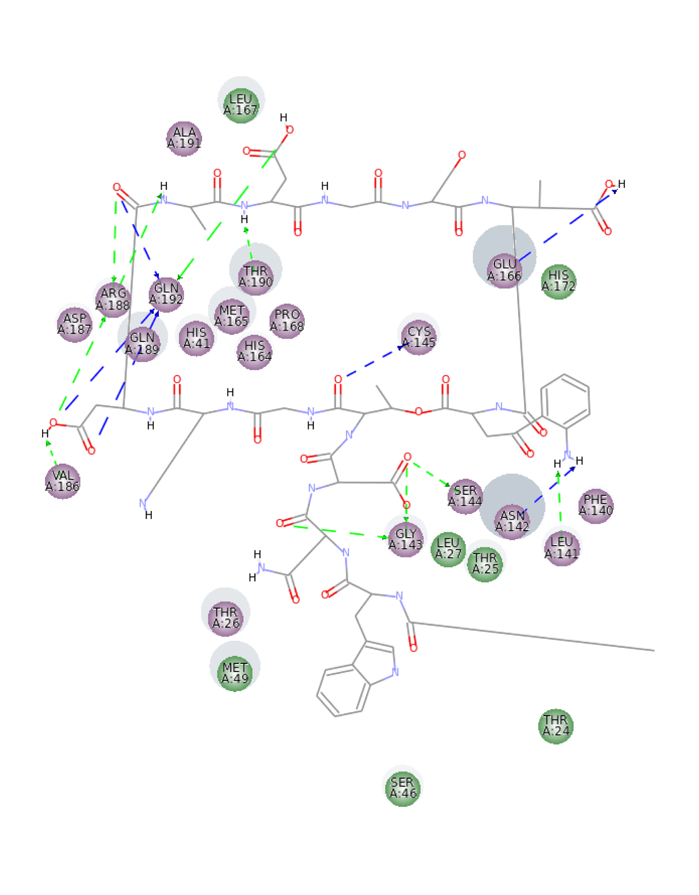 |
| 4 | Enfuvirtide | 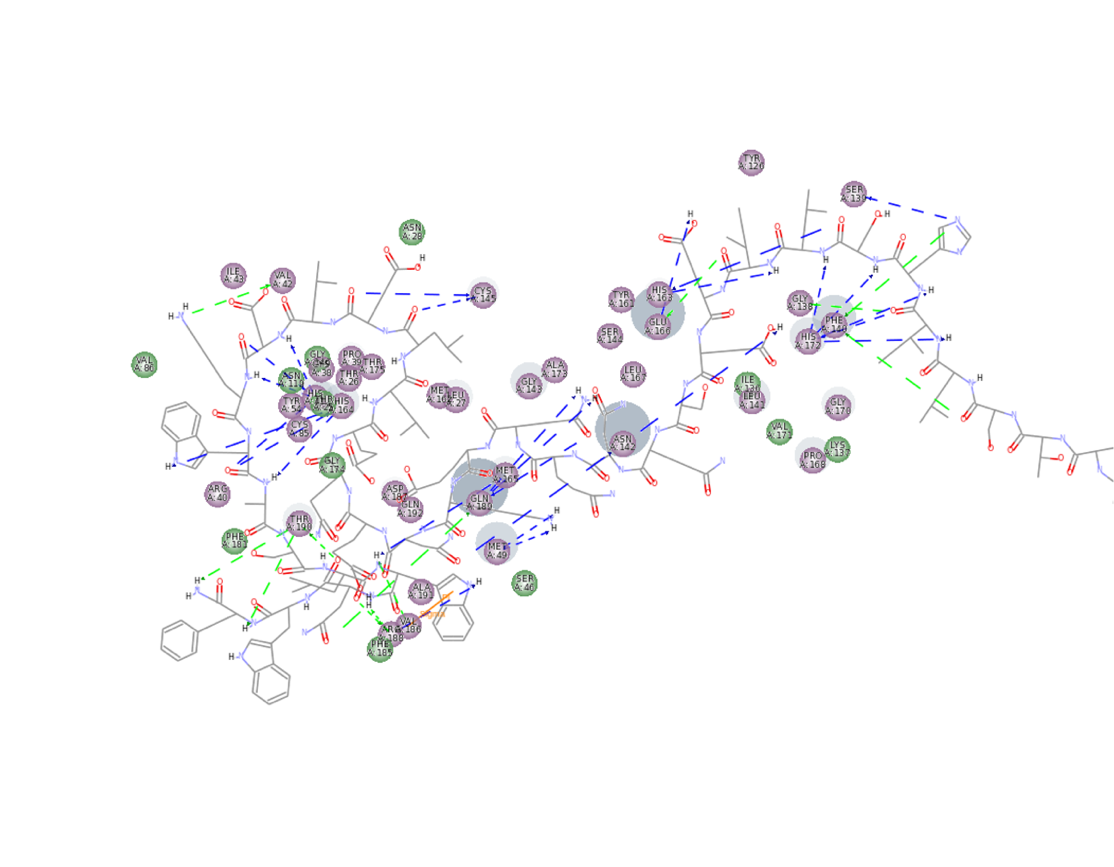 |
|  |  | WITHDRAWN DRUGS / REFERENCE MOLECULE: N3 |
| 5 | Romidepsin | 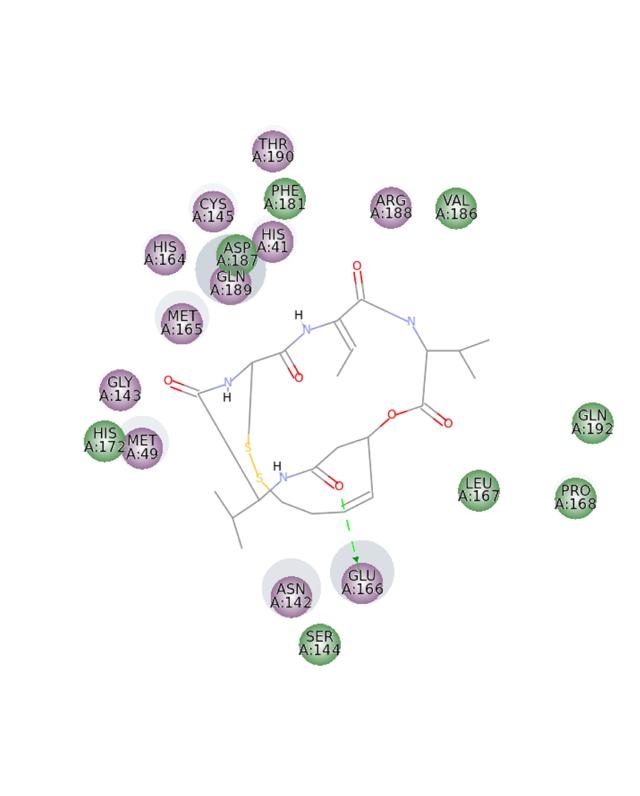 |
| 6 | Abarelix | 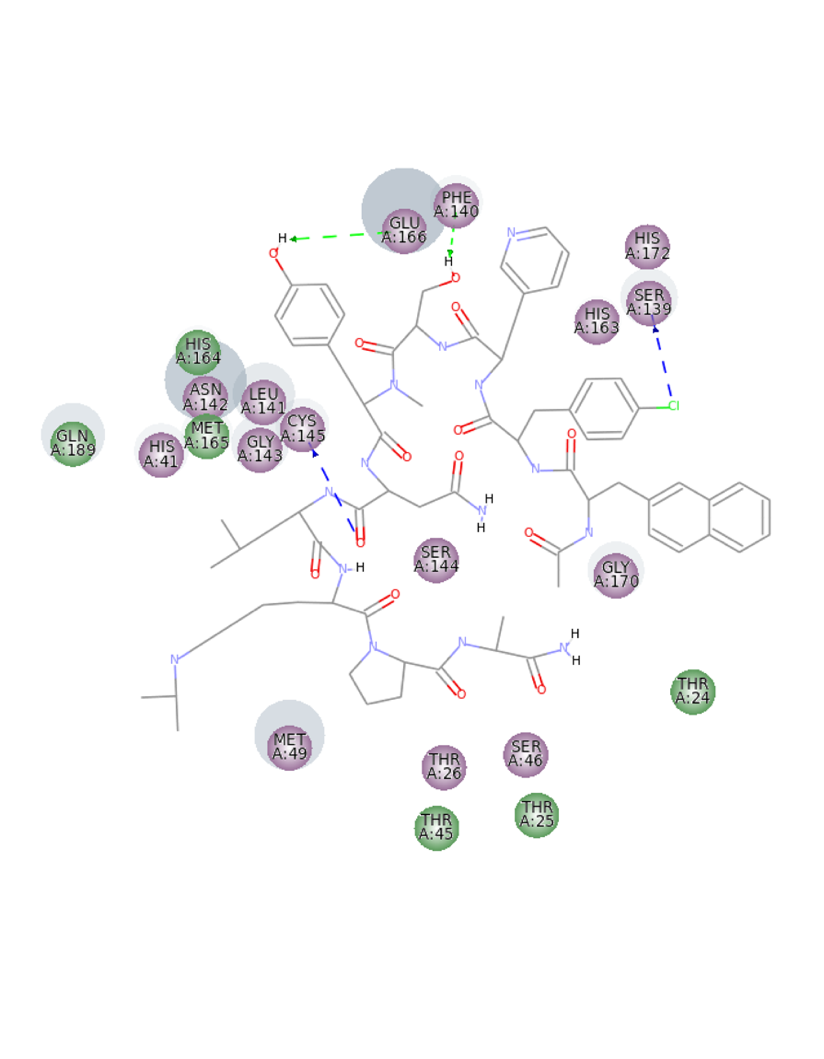 |
| 7 | Trabectedin | 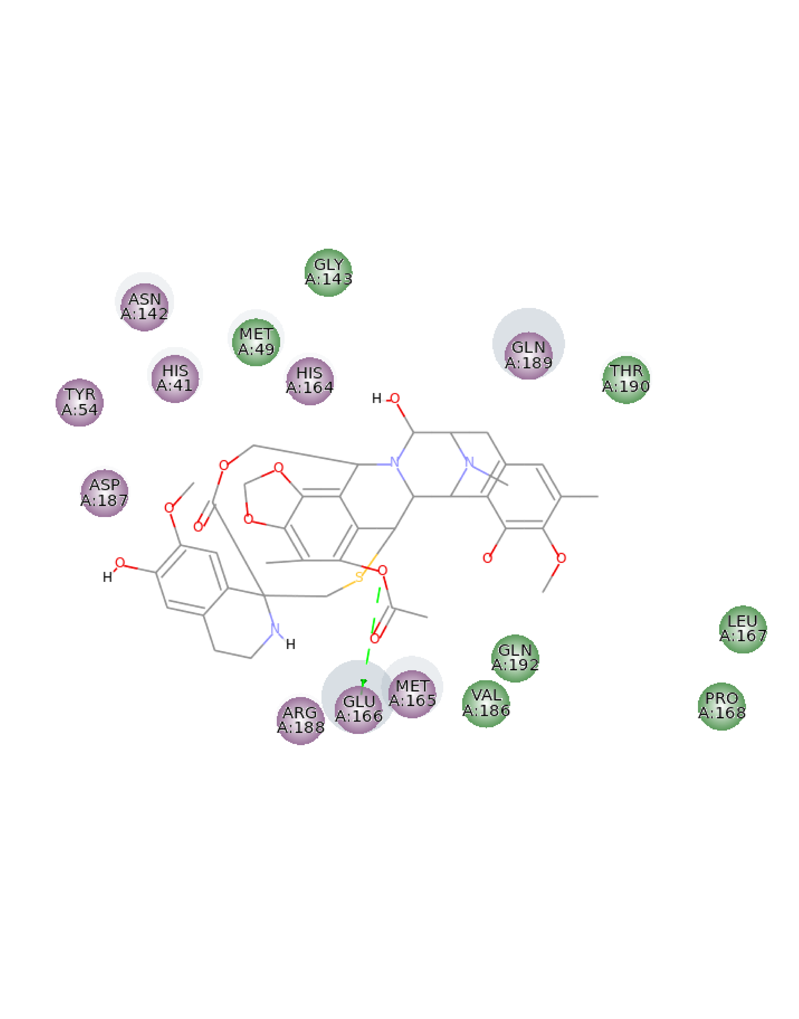 |
| 8 | Topotecan | 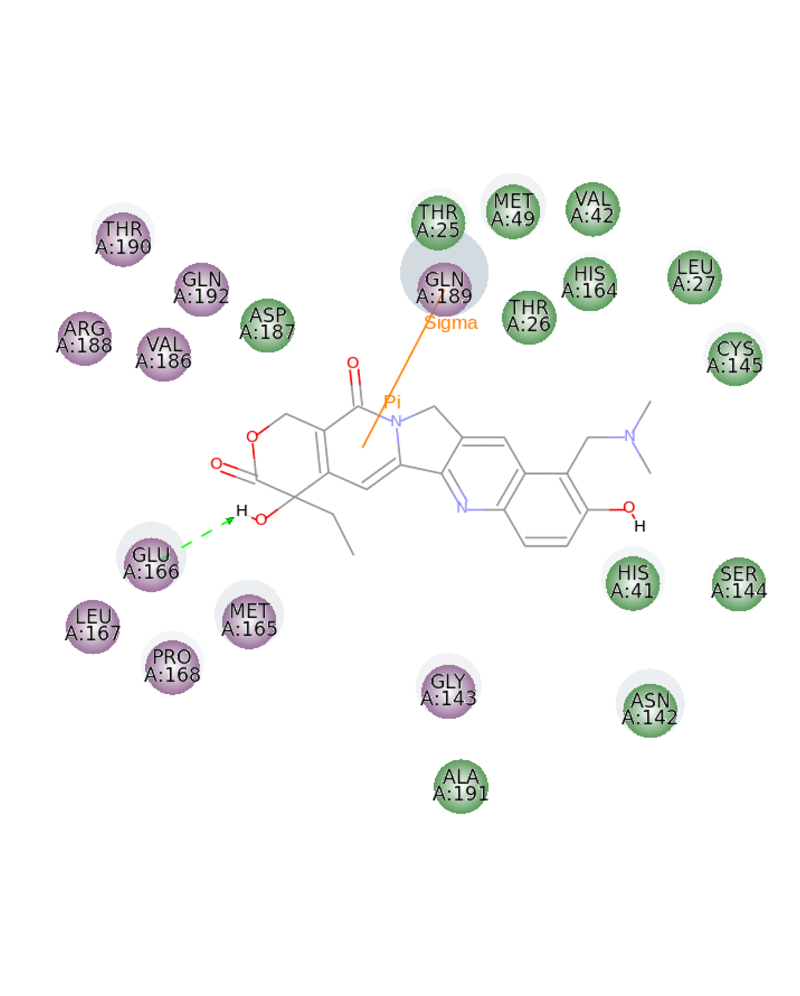 |
| 9 | Rescinnamine | 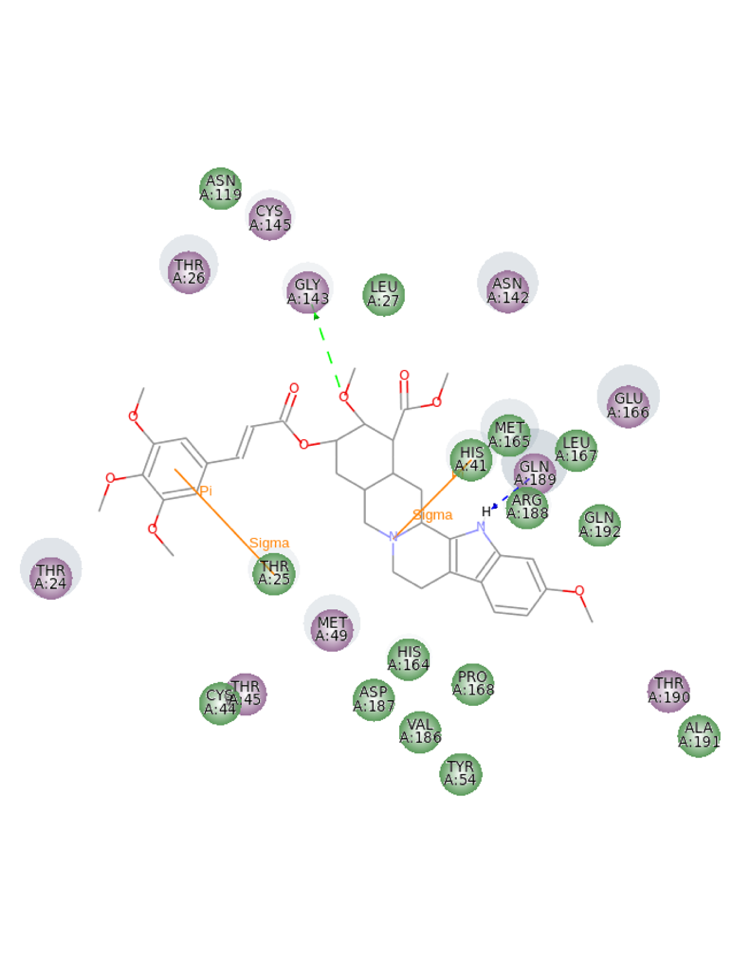 |
| 10 | Dirithromycin | 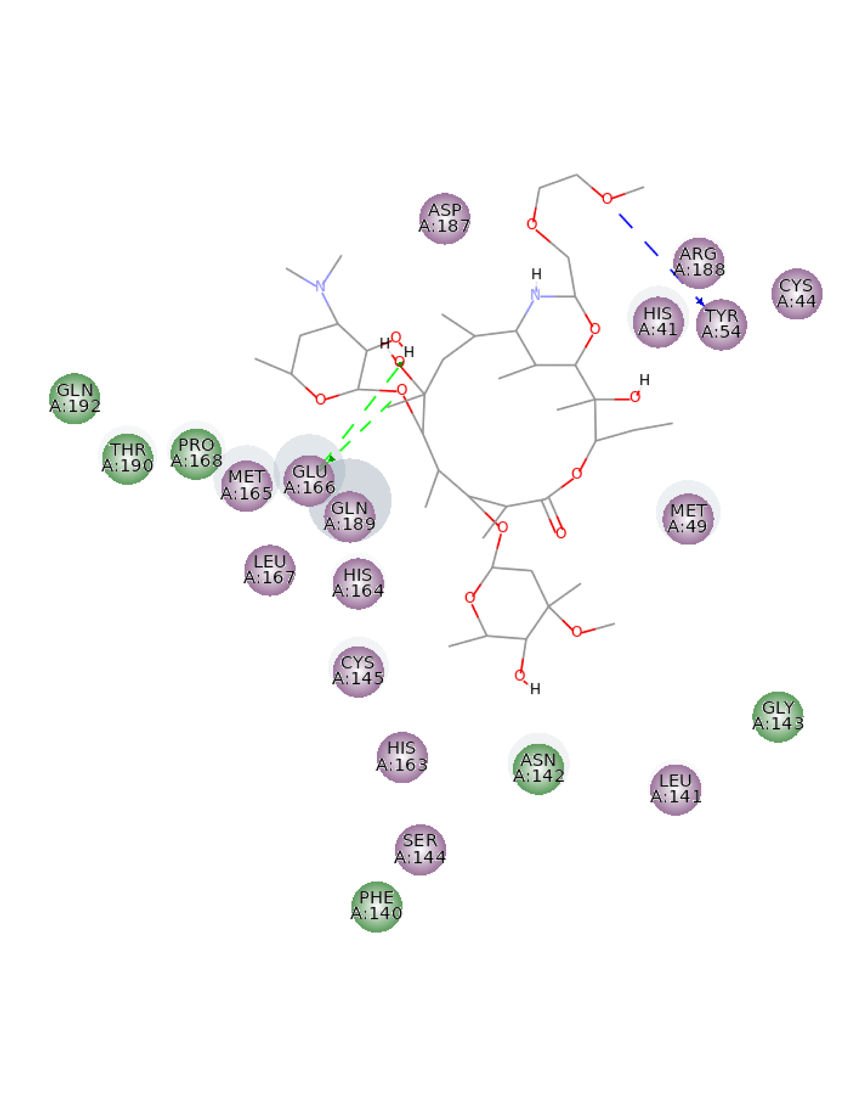 |
|  |  | TCM COMPOUNDS / REFERENCE MOLECULE: N3 |
|  |  |  |
| 11 | Nummularine A | 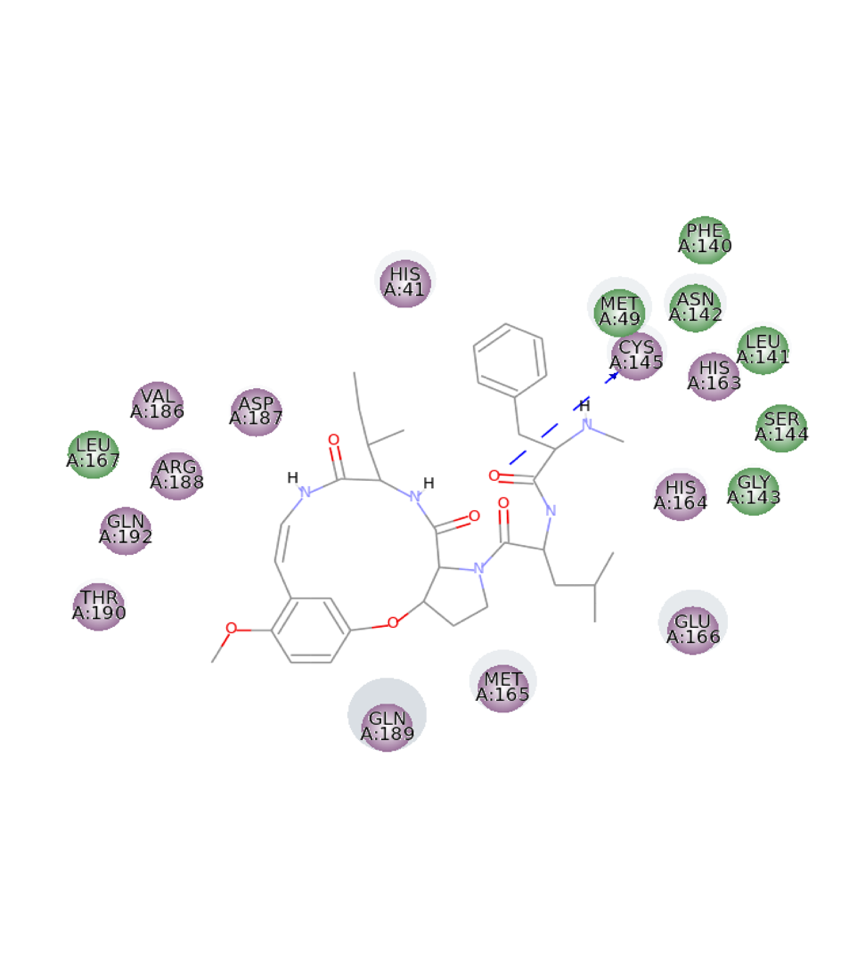 |
| 12 | Notoamide G | 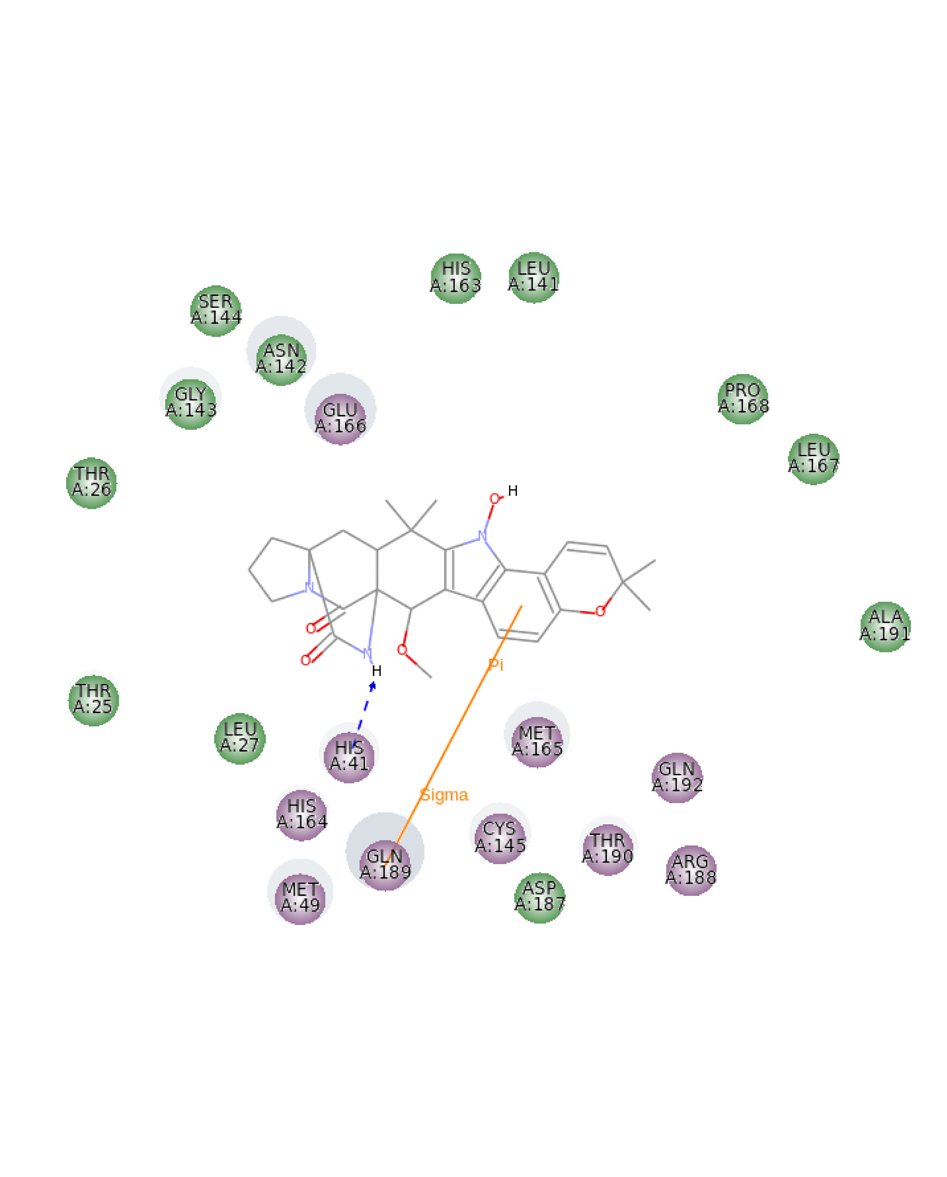 |
| 13 | Jubanine A | 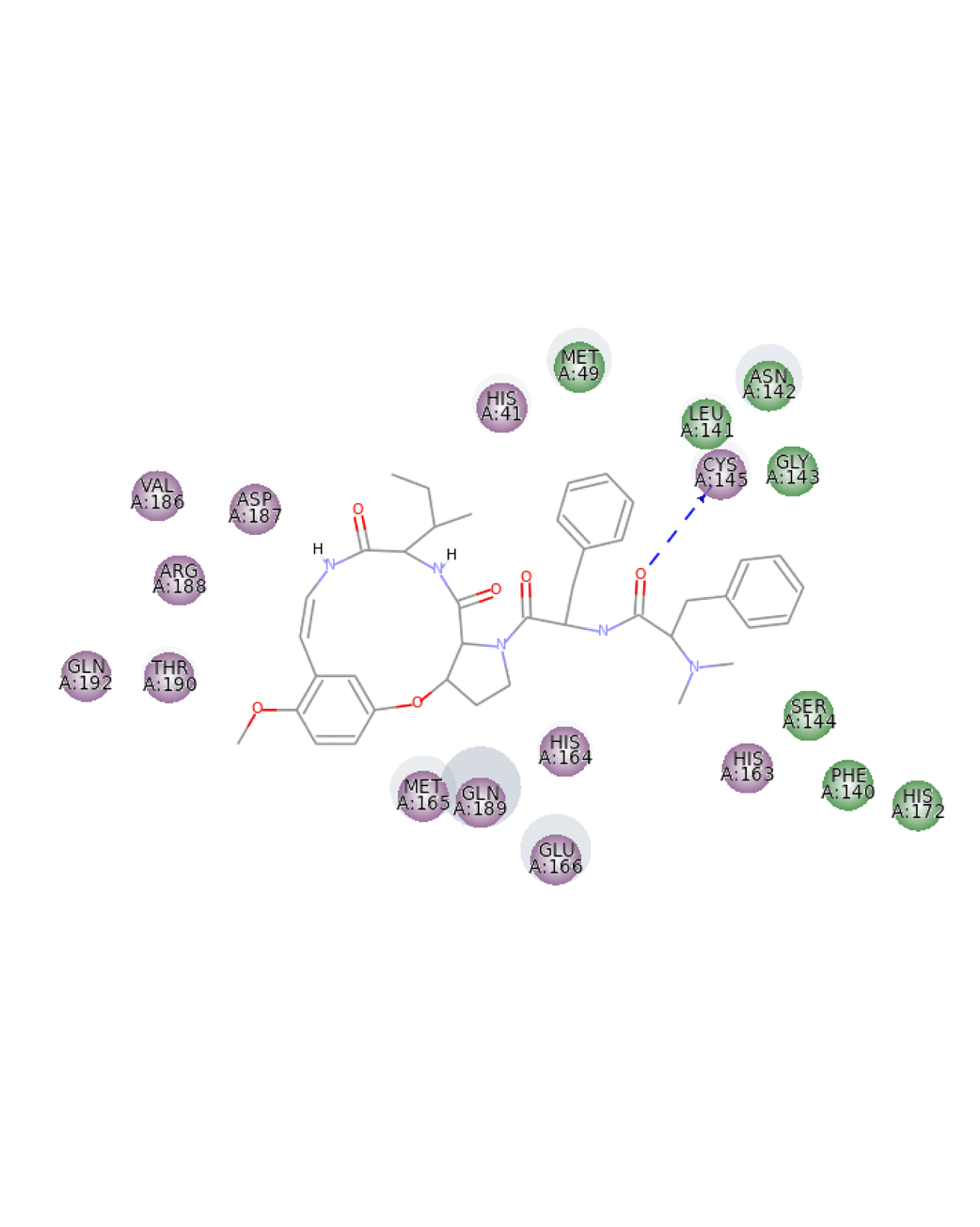 |
| 14 | Mauritine A | 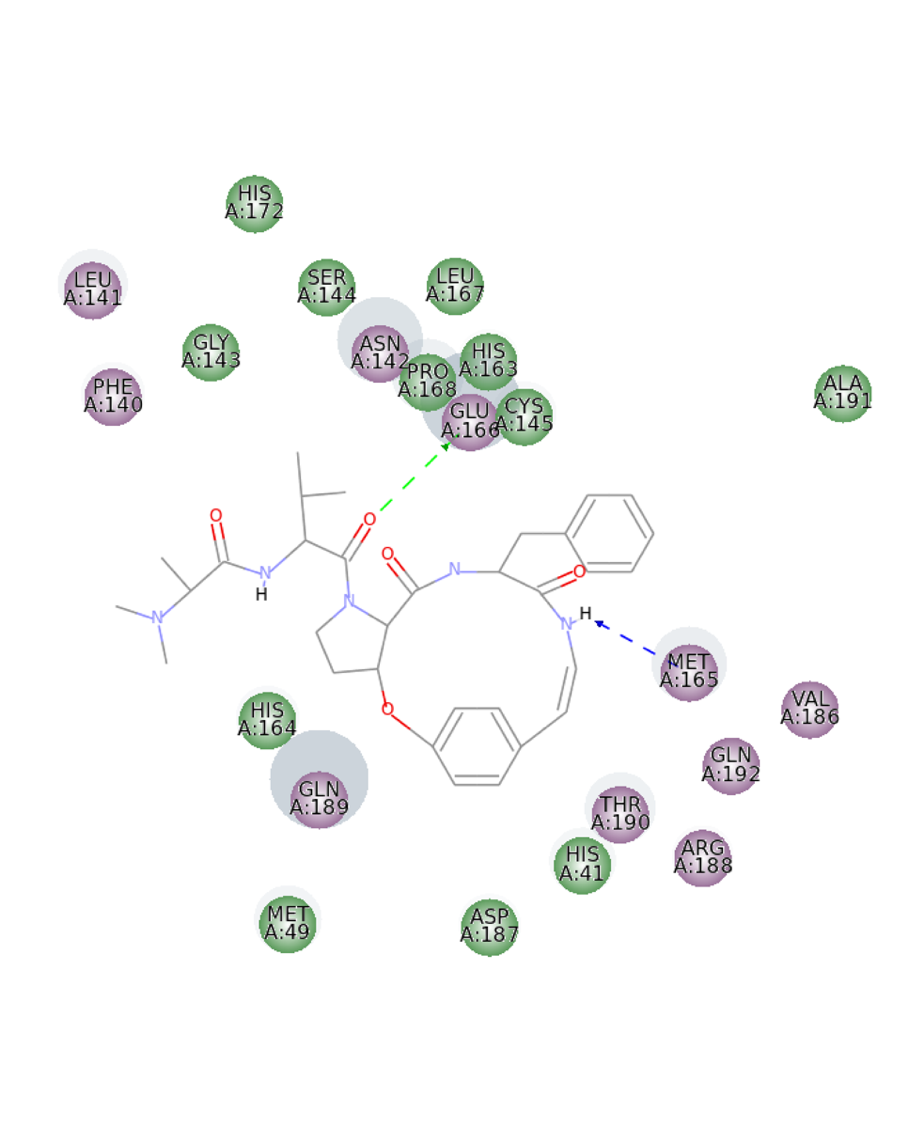 |
| 15 | Mauritine B | 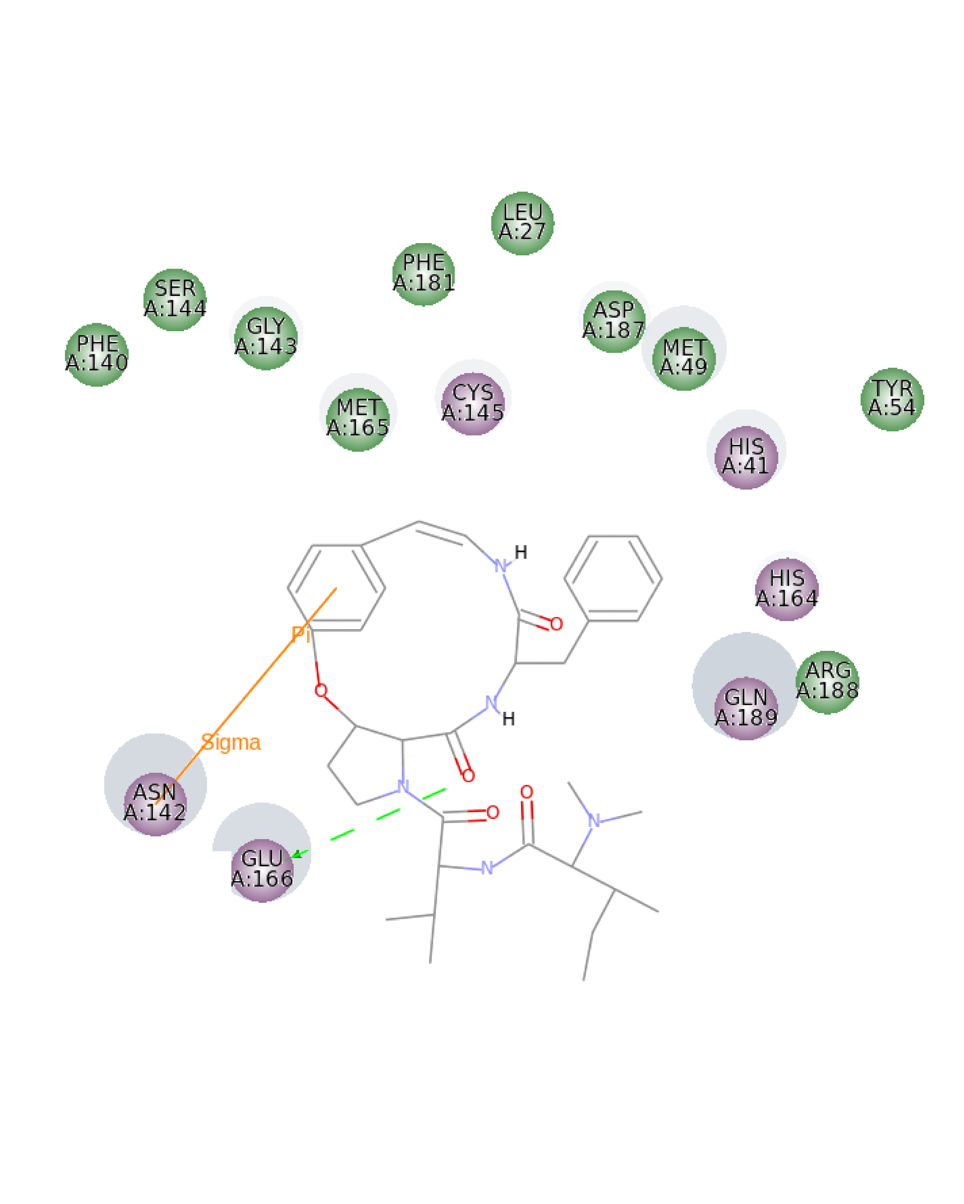 |
|  |  | SUPERNATURAL COMPOUNDS / REFERENCE MOLECULE: N3 |
| 16 | SN00308384 | 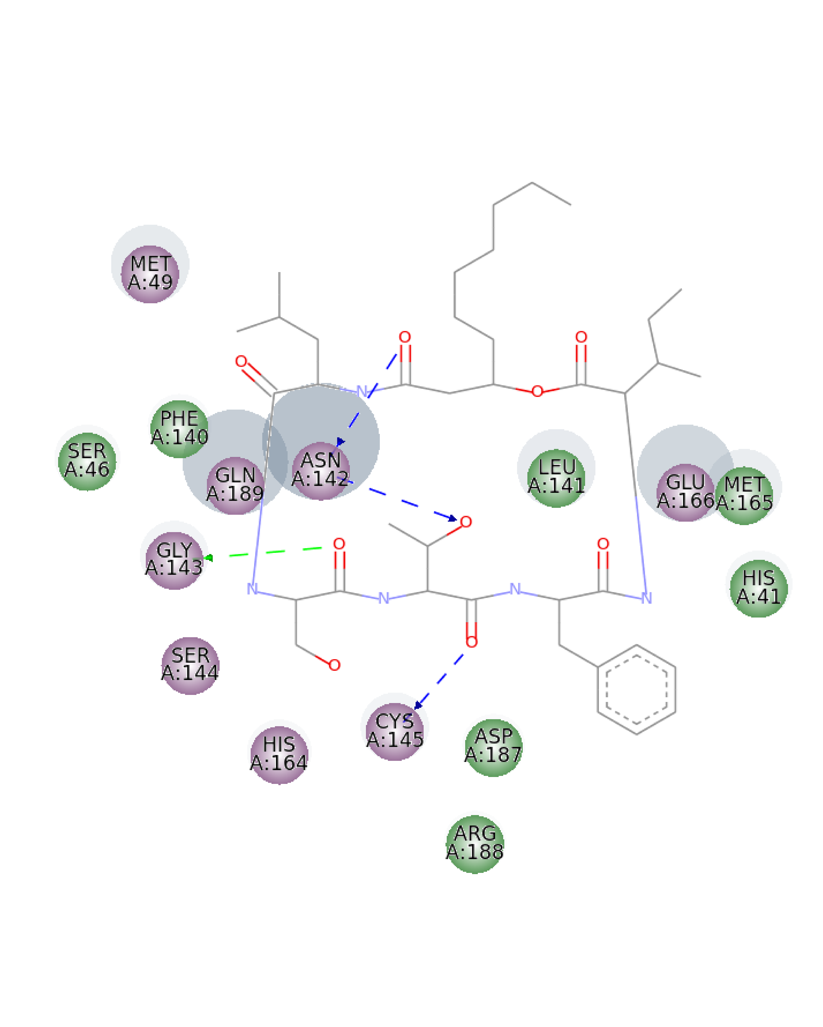 |
| 17 | SN00227324 | 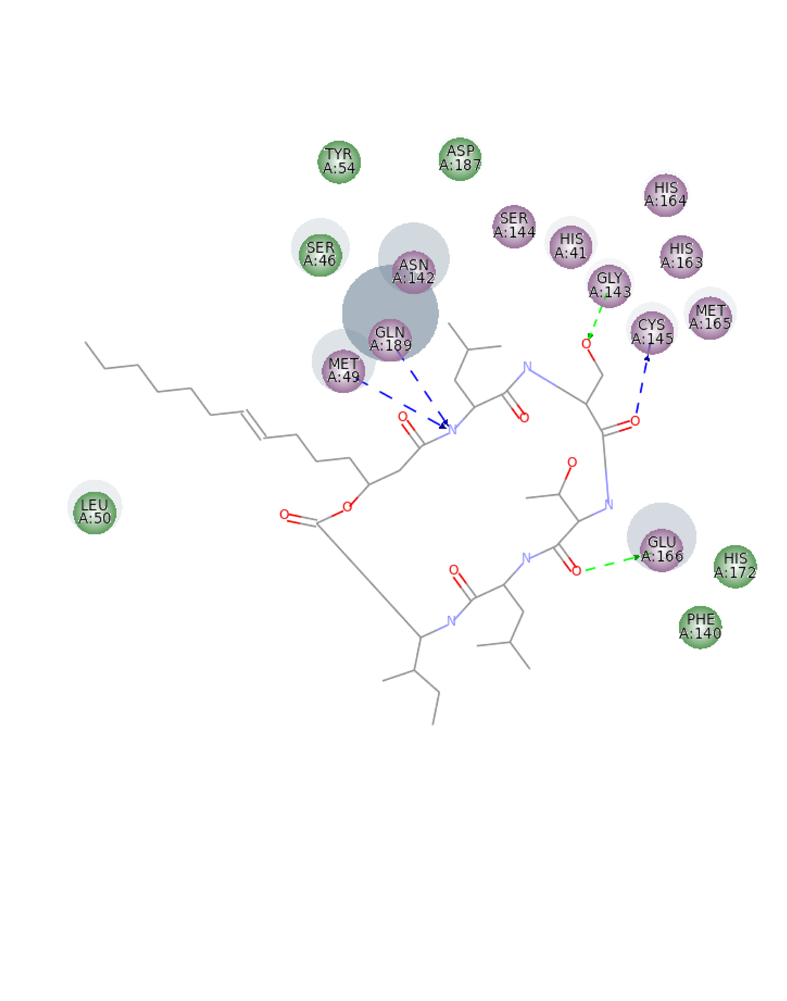 |
| 18 | SN00270610 | 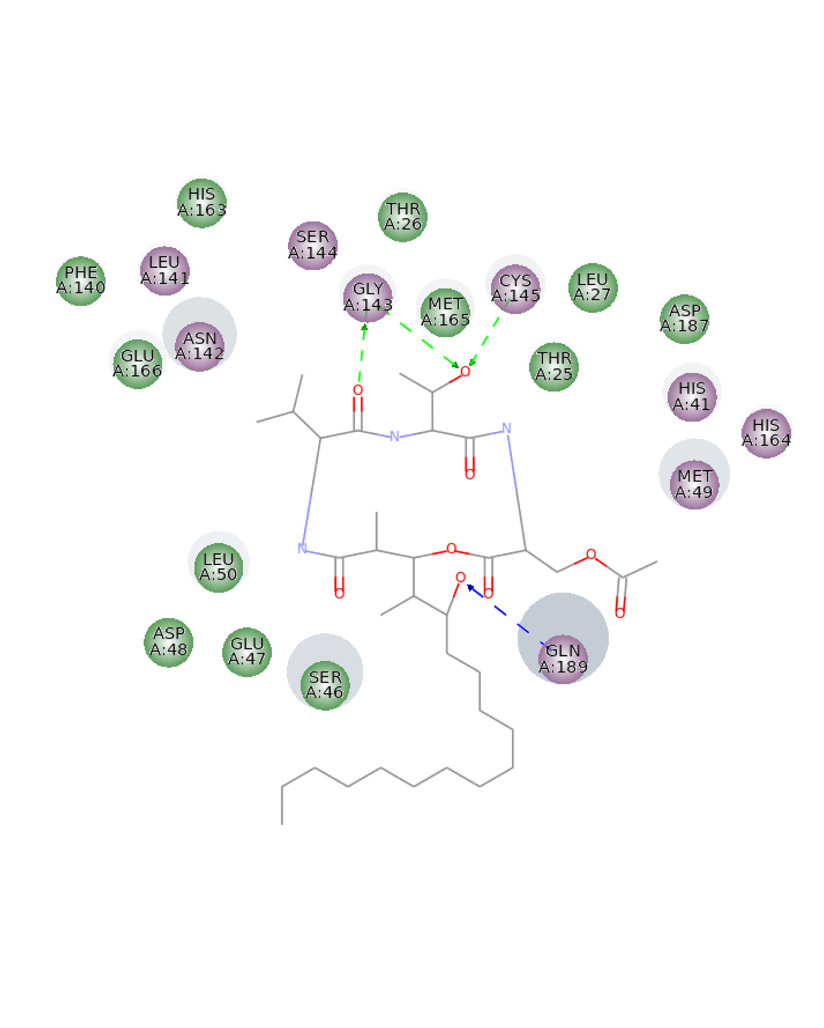 |
| 19 | SN00354661 | 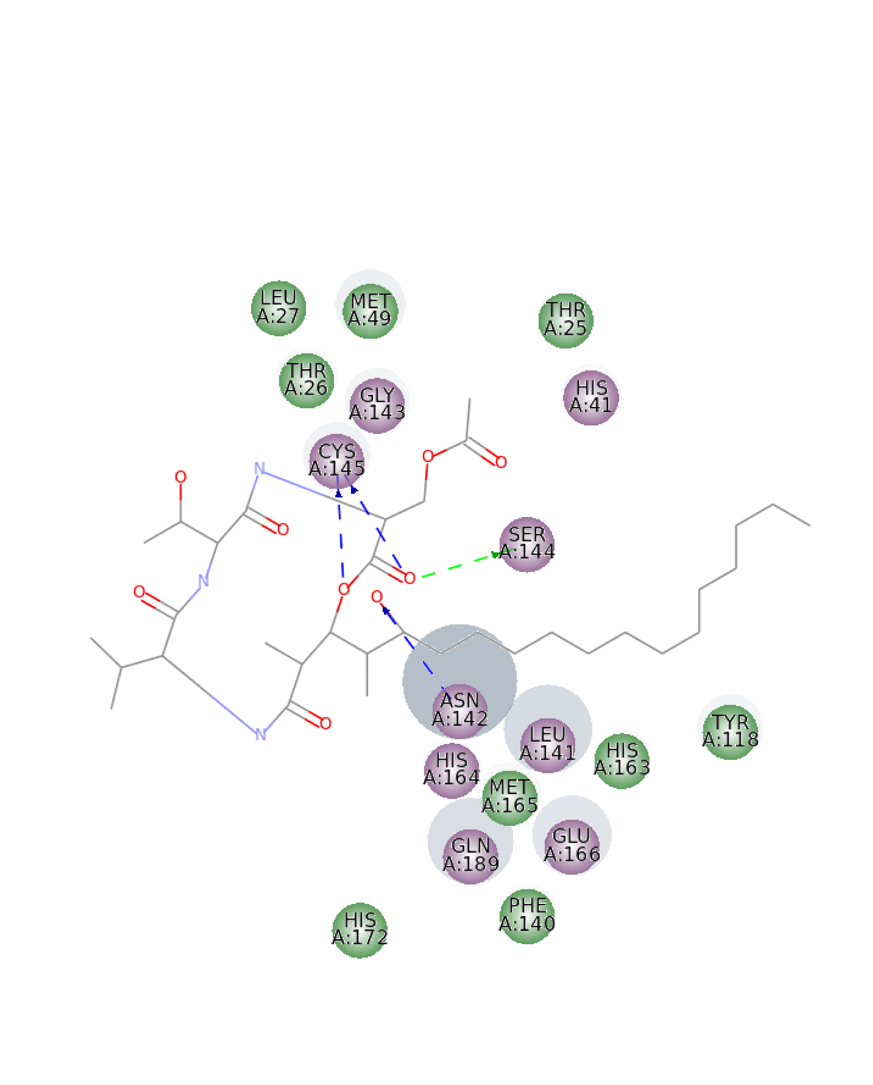 |
| 20 | SN00254530 | 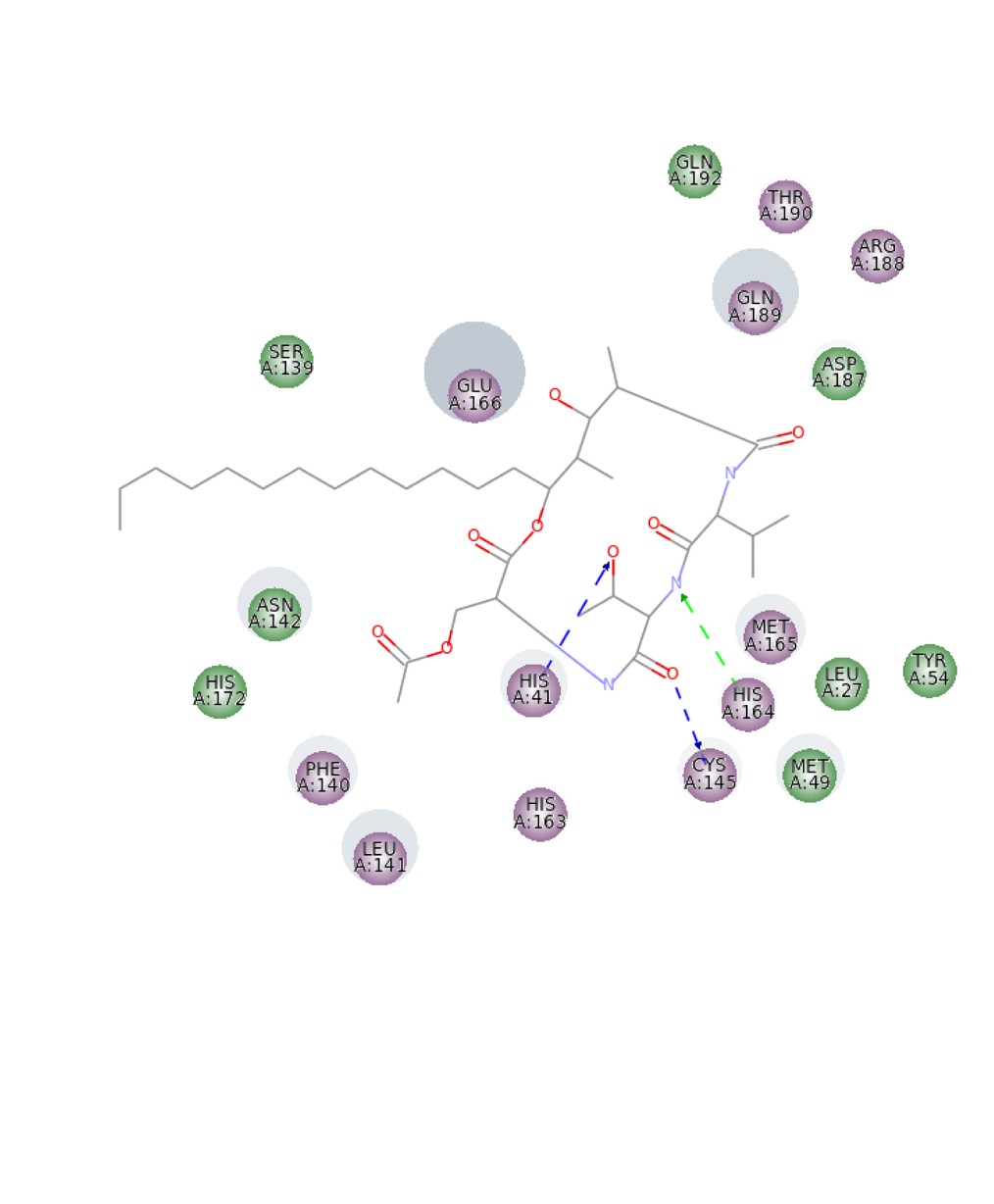 |
| 21 | SN00314990 | 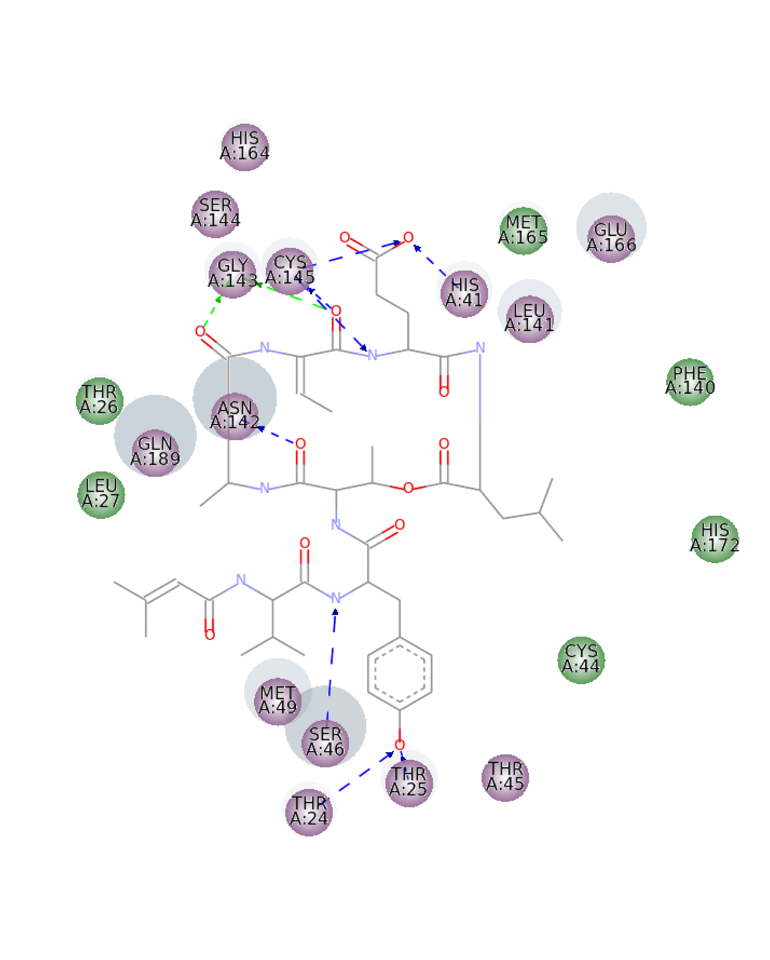 |
| 22 | SN00238988 | 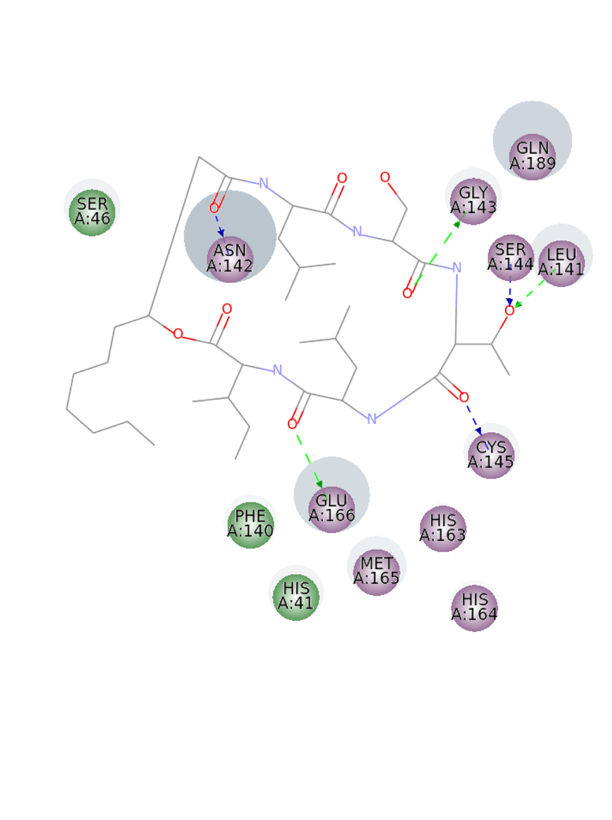 |
|  |  | APPROVED DRUGS / REFERENCE MOLECULE: O6K |
| 23 | Lopinavir | 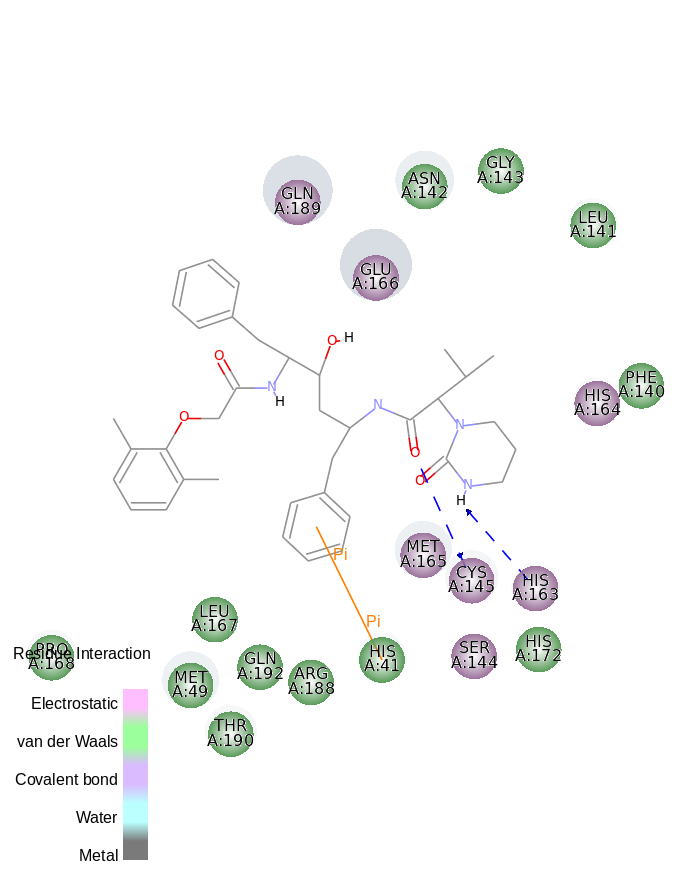 |
| 24 | Angiotensinamide | 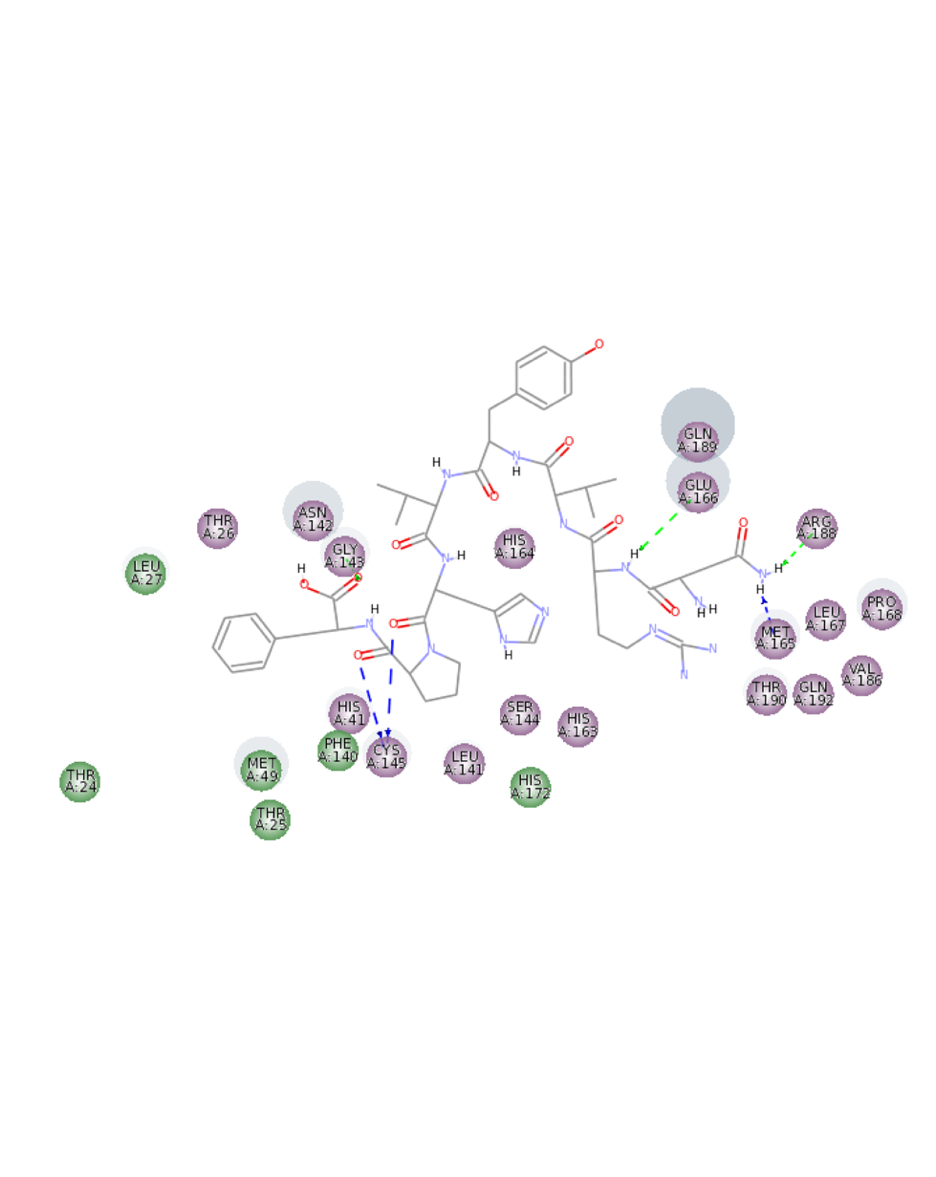 |
| 25 | Indinavir | 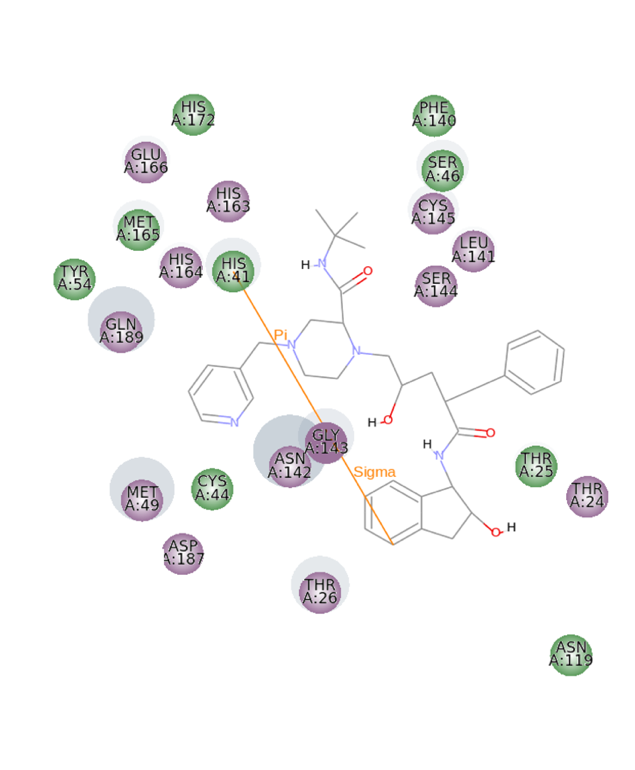 |
| 26 | Dihydroergocornin E | 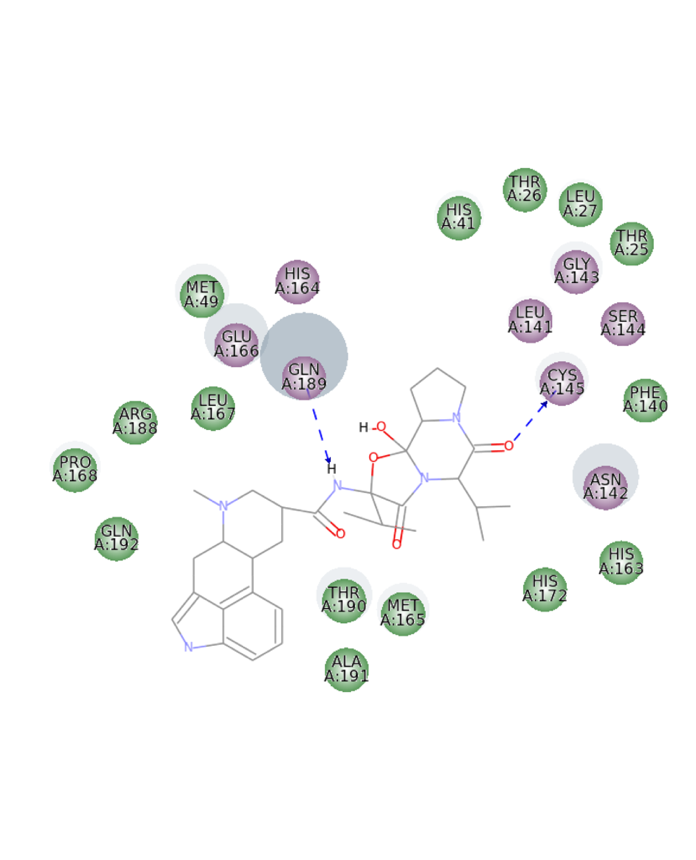 |
| 27 | DihydroergocryptinE | 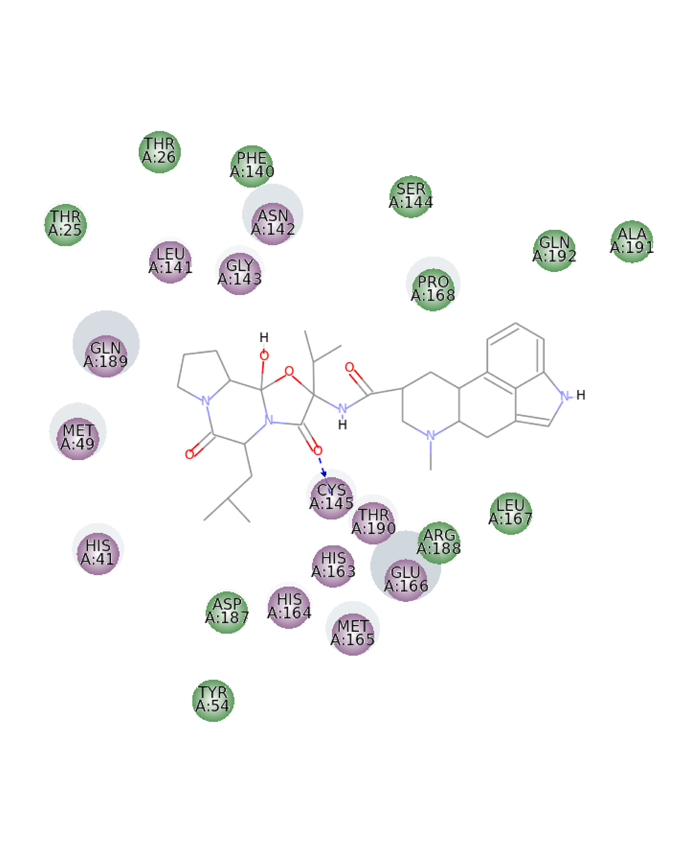 |
| 28 | Epicriptine | 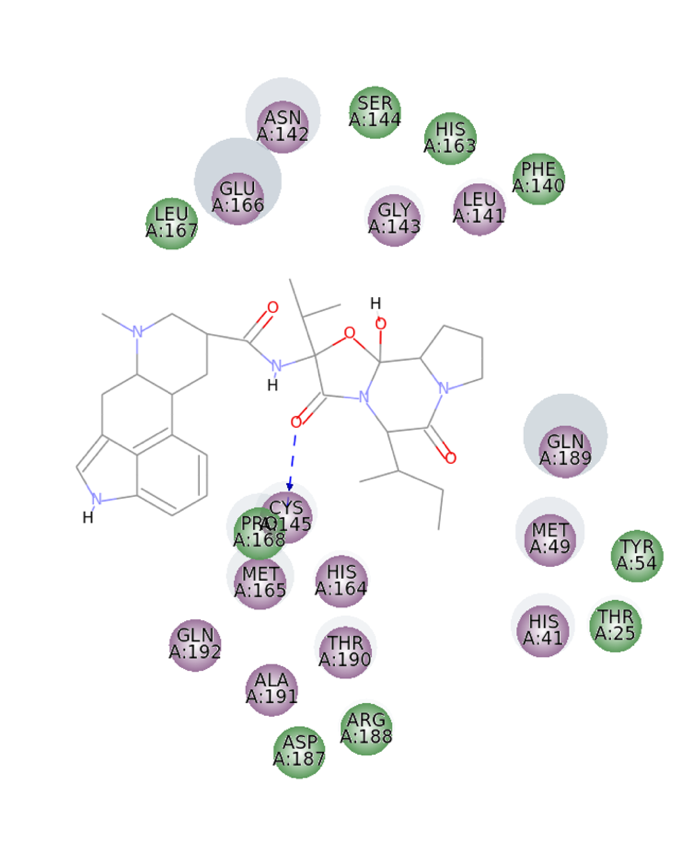 |
| 29 | Bivalirudin | 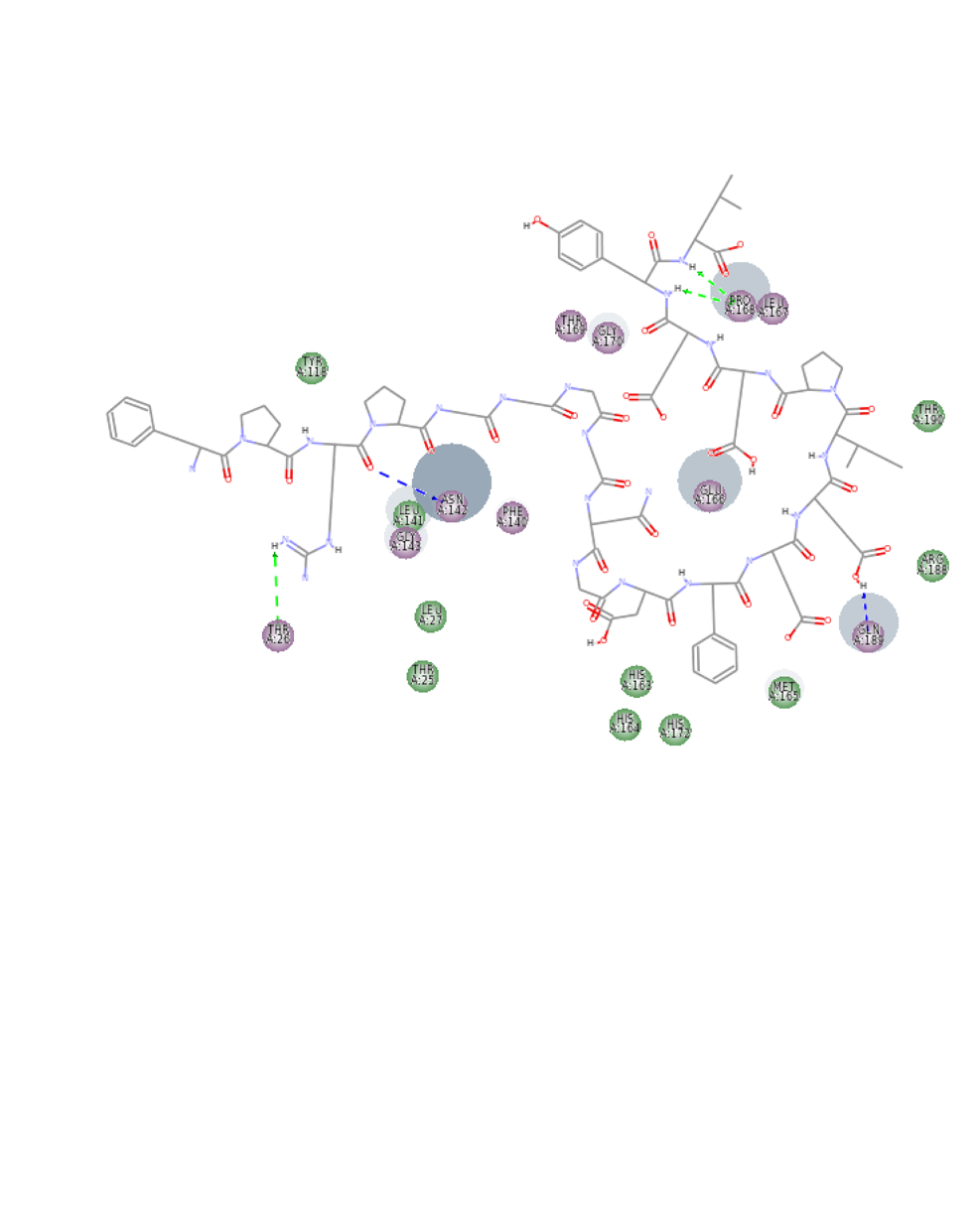 |
| 30 | Dihydroergotamin E | 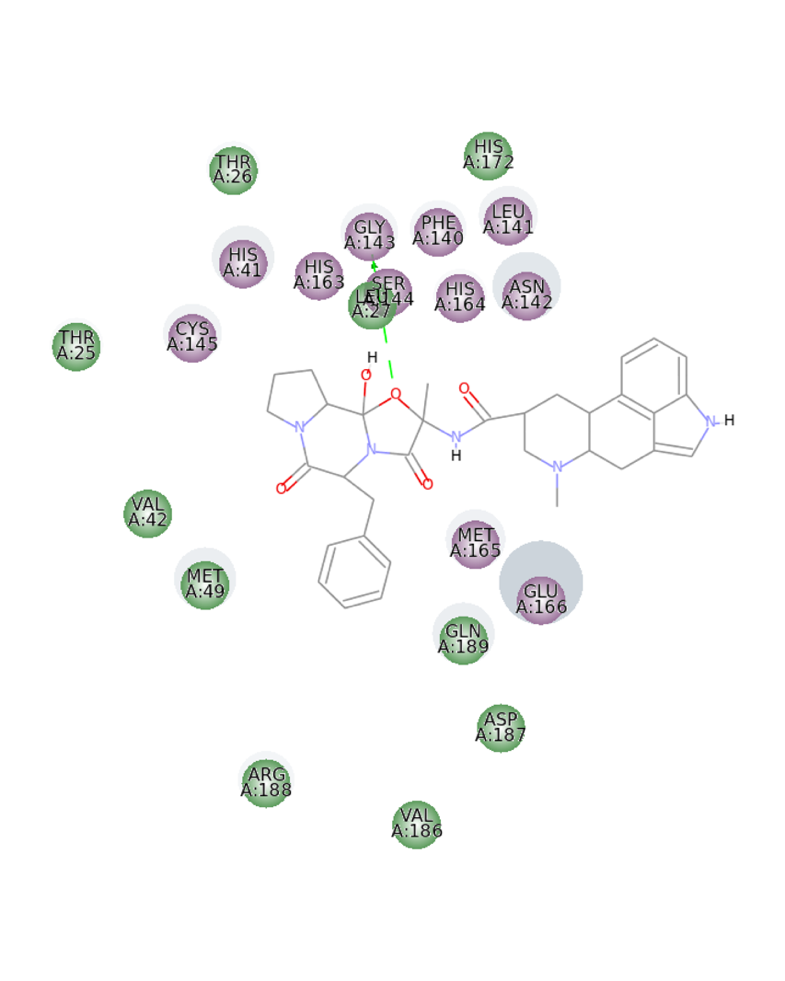 |
| 31 | Telaprevir | 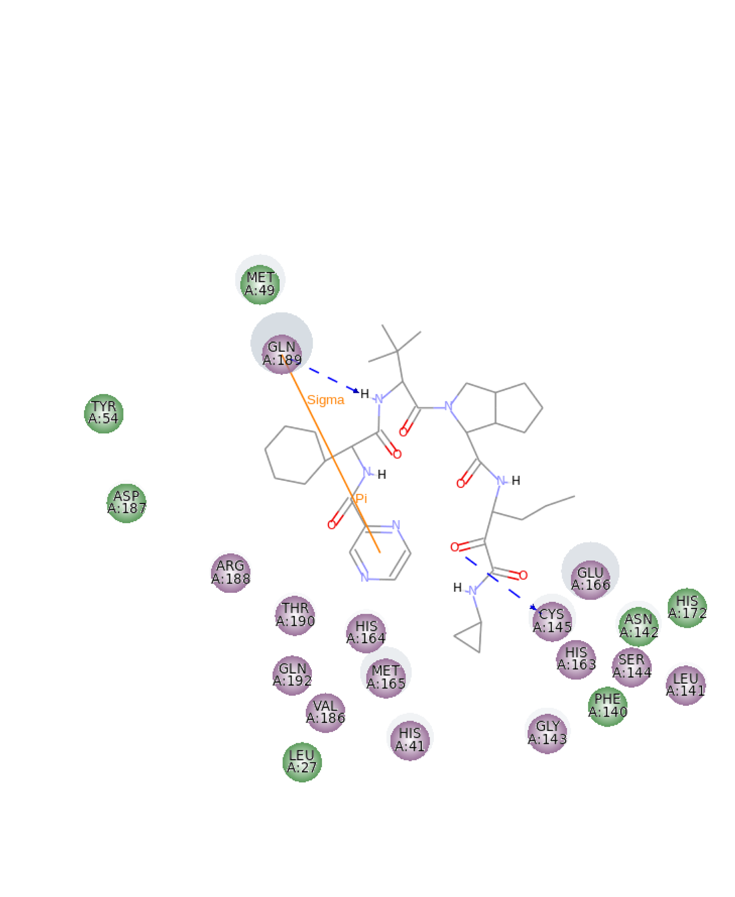 |
| 32 | Alpha-Ergocryptine | 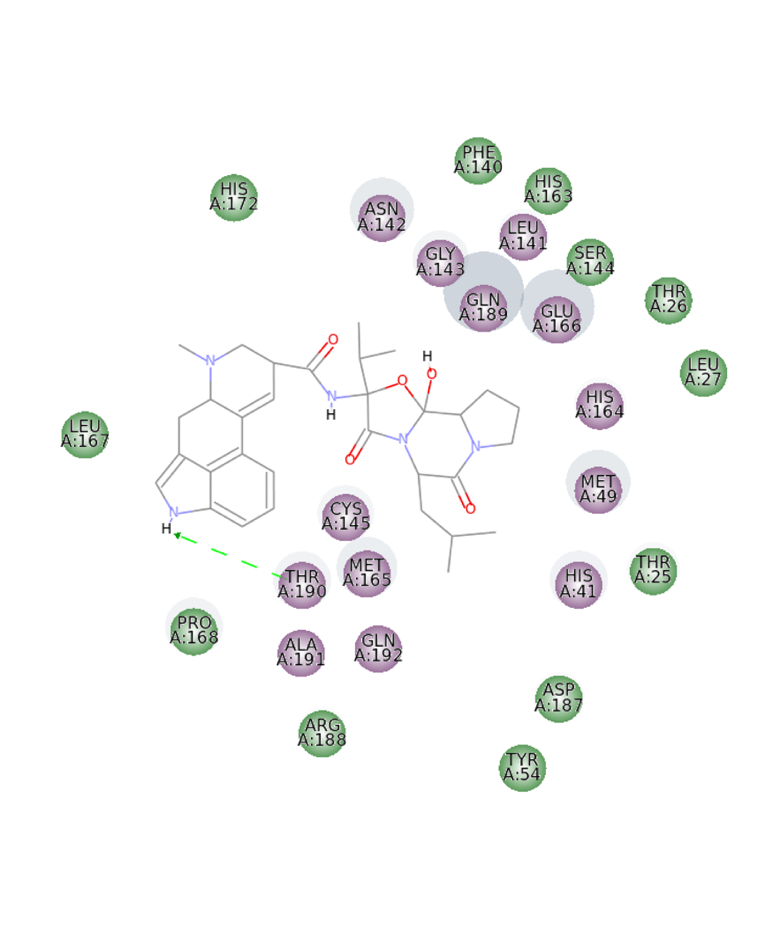 |
|  |  | WITHDRAWN DRUGS / REFERENCE MOLECULE: O6K |
| 33 | Lypressin | 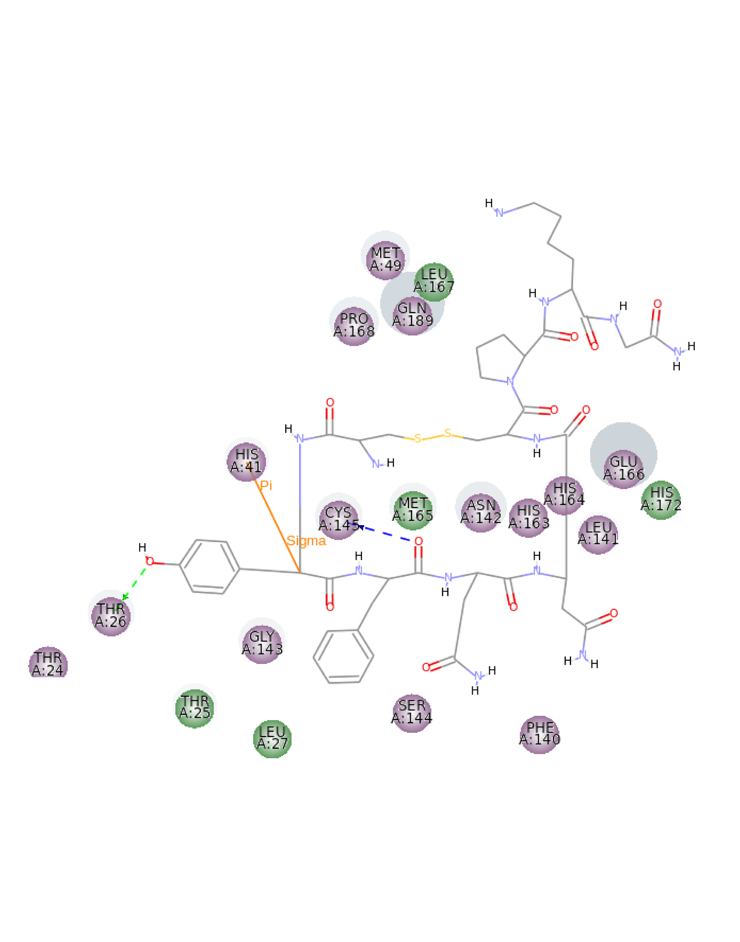 |
| 34 | Alatrofloxacin | 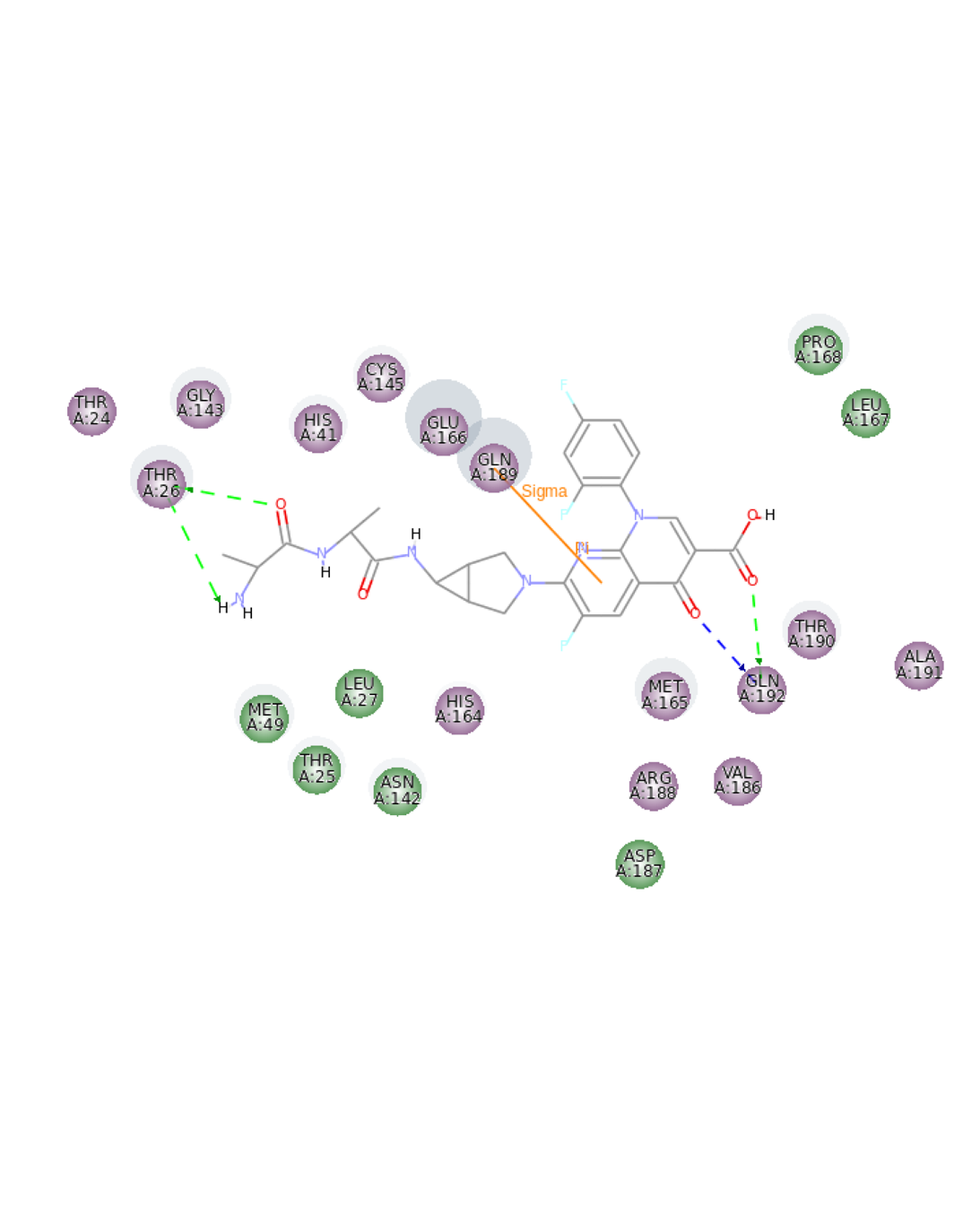 |
| 35 | Nelfinavir | 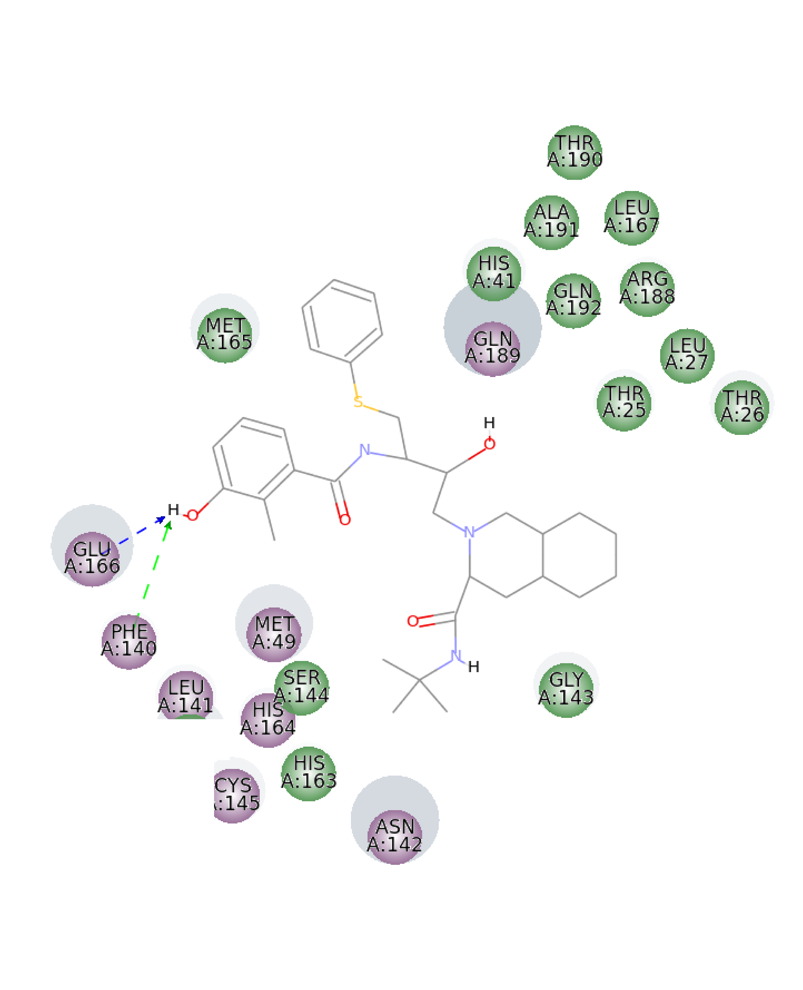 |
| 36 | Pentagastrin | 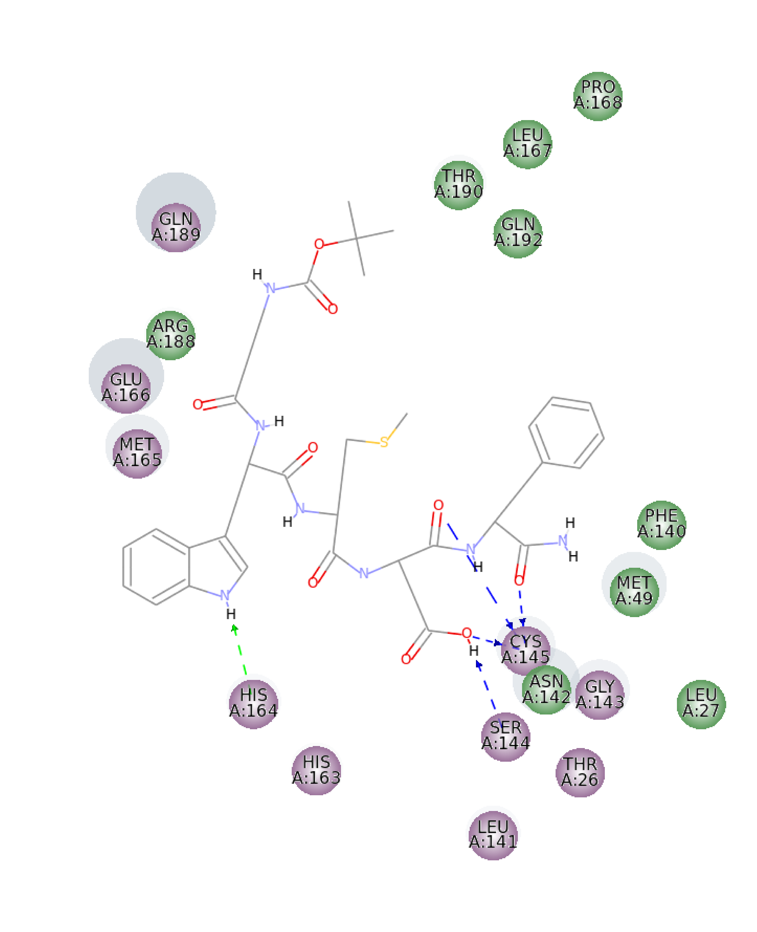 |
| 37 | Azlocillin | 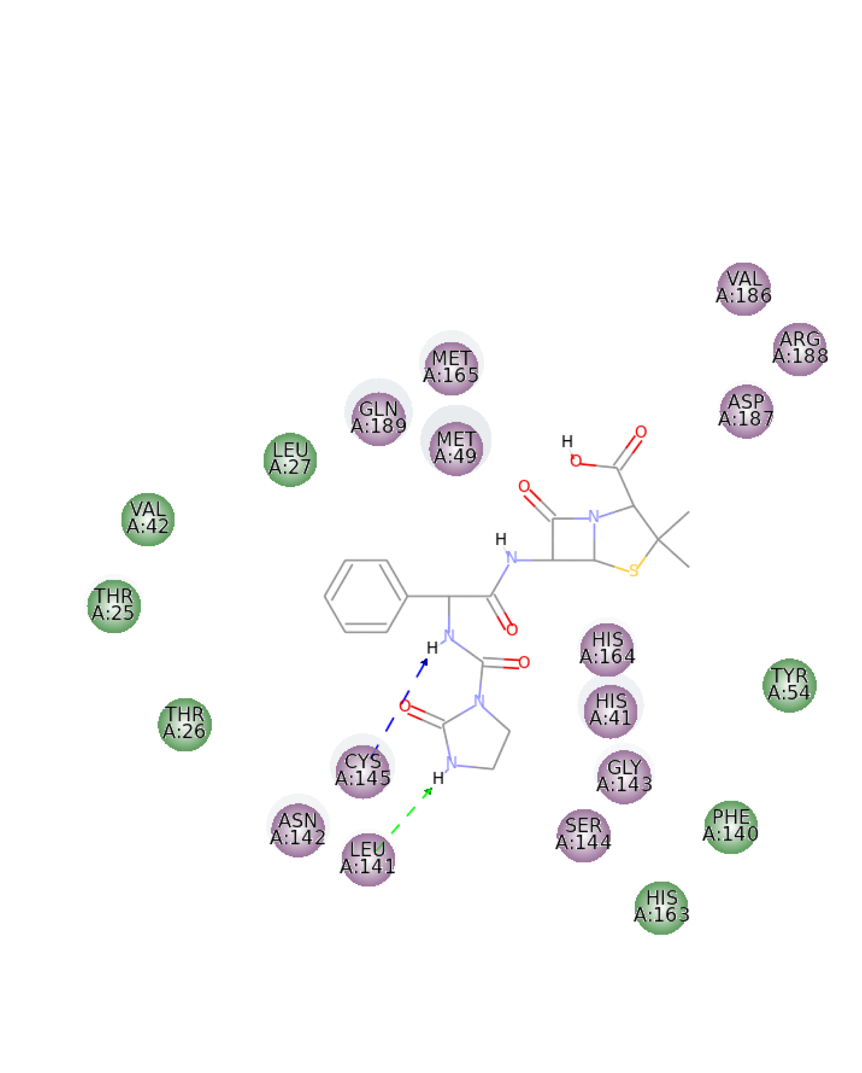 |
| 38 | Telithromycin | 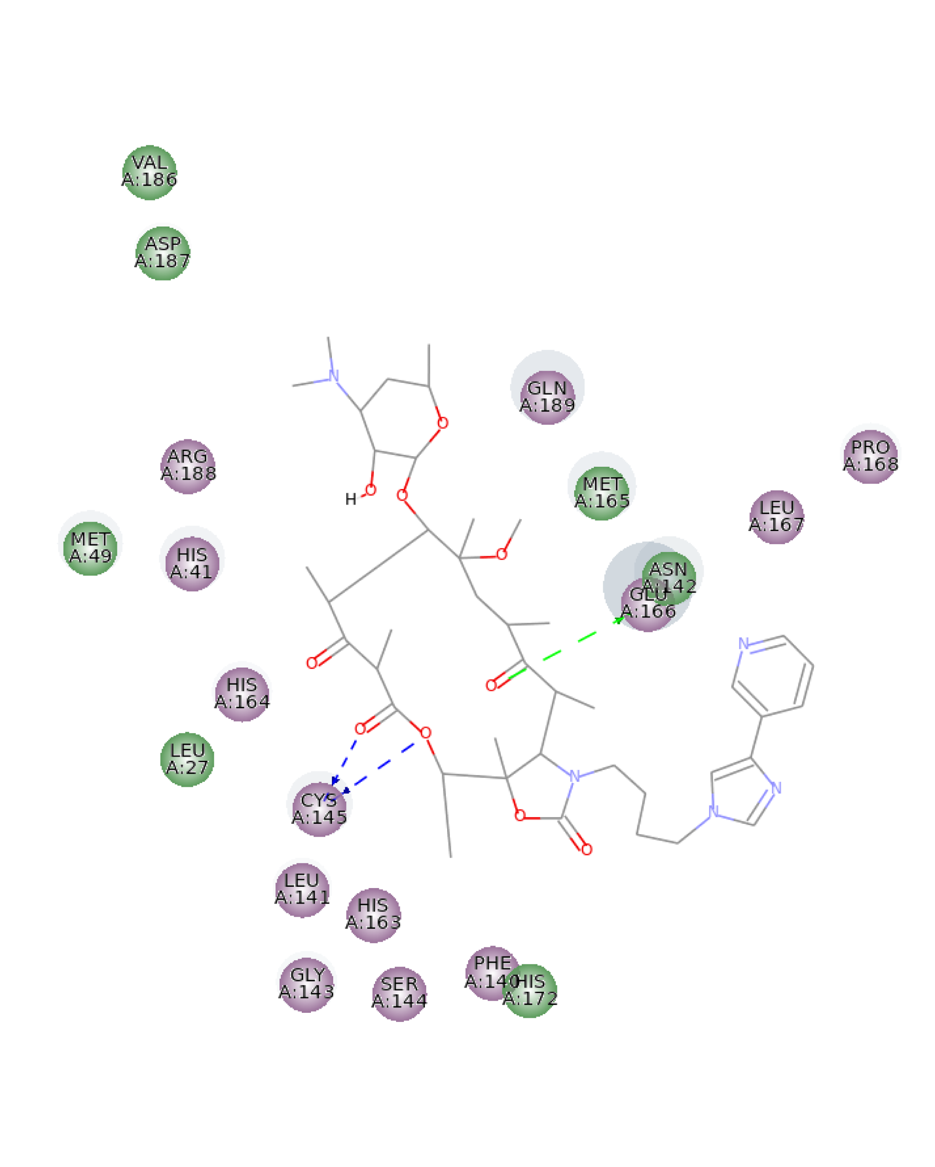 |
|  |  | TCM COMPOUNDS / REFERENCE MOLECULE: O6K |
| 39 | Hirudin | 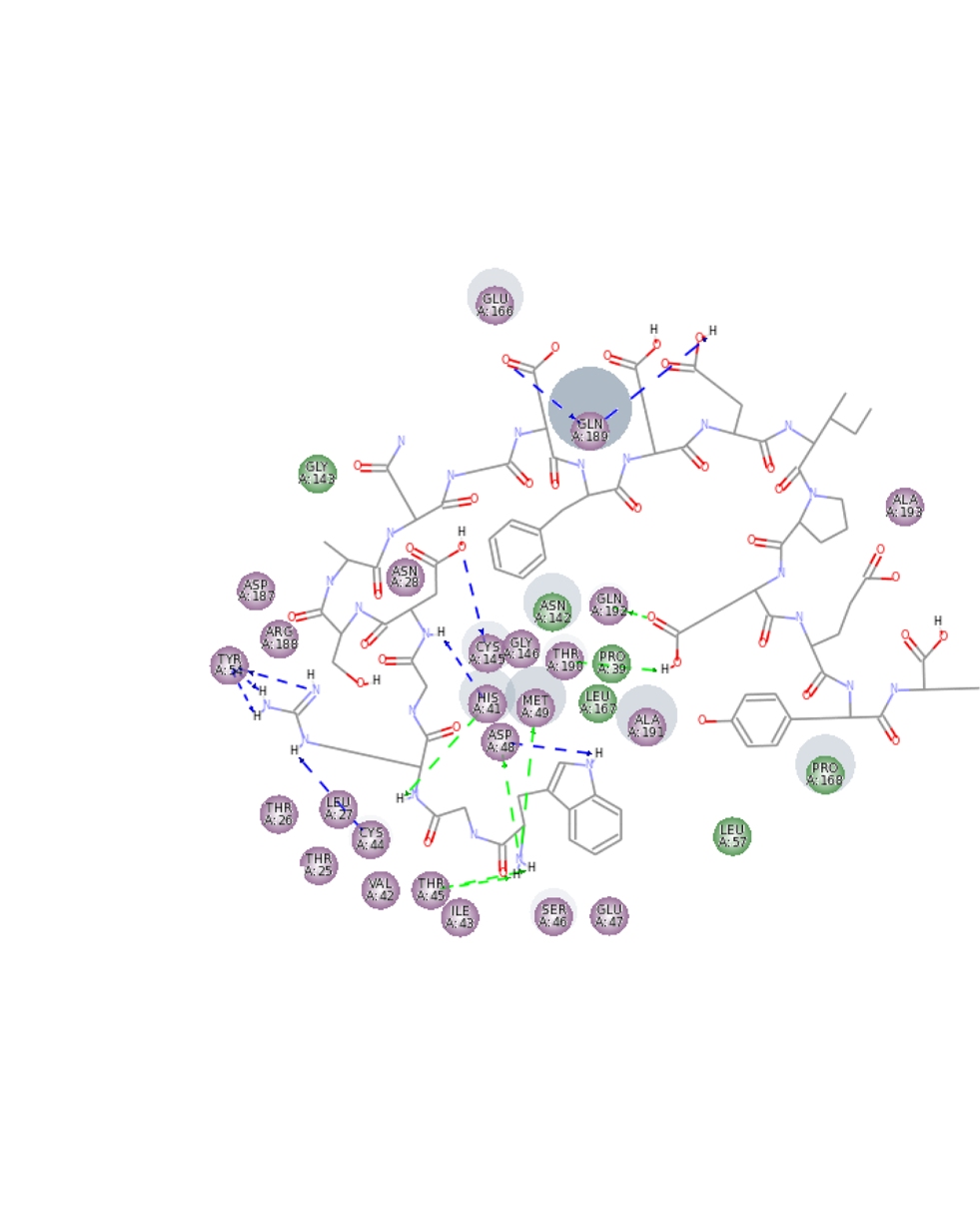 |
| 40 | Segetalin E | 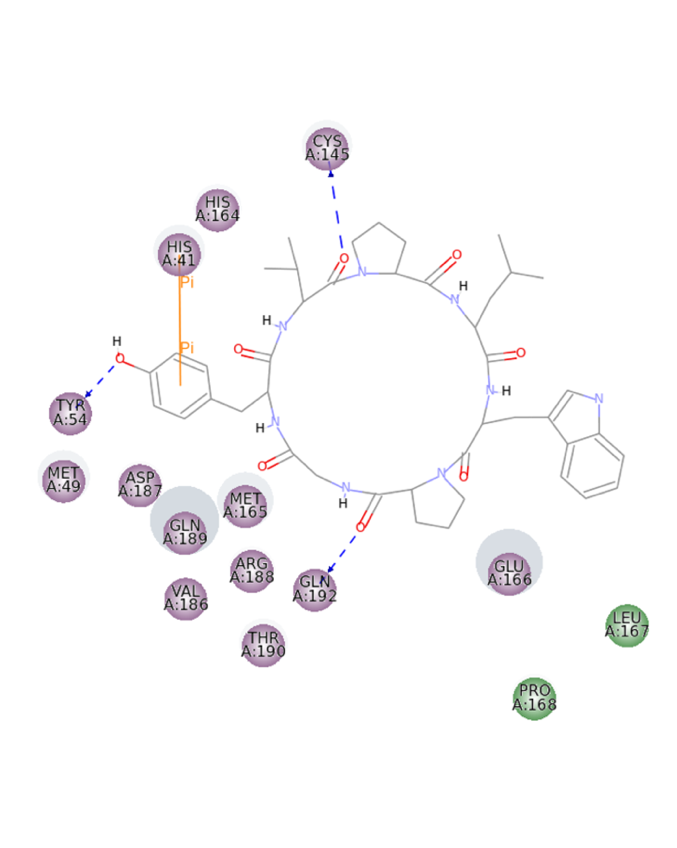 |
| 41 | Lyciumin C | 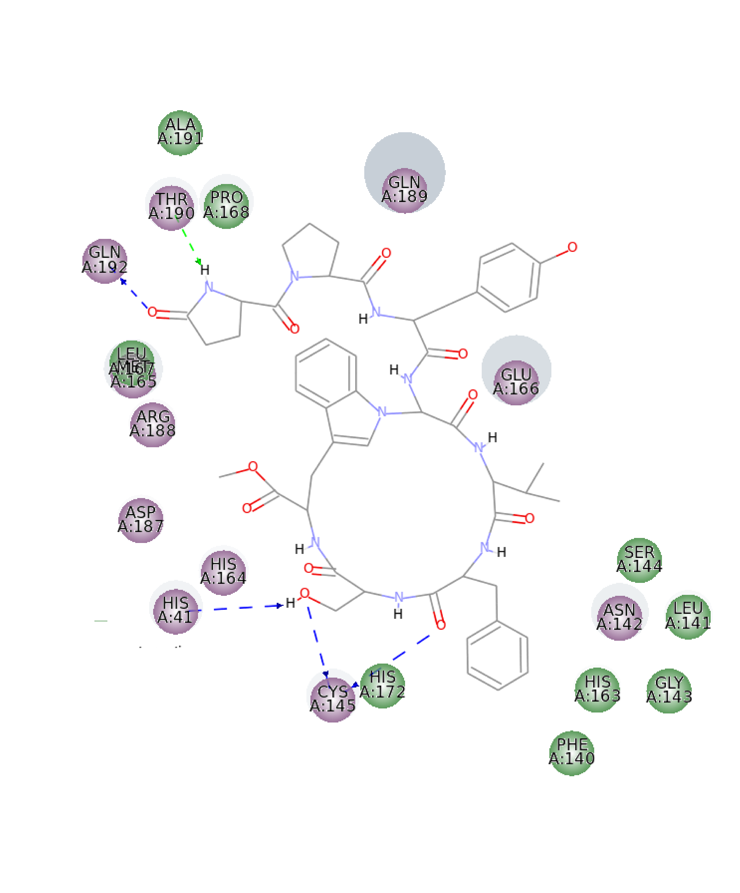 |
| 42 | Celogenamide A | 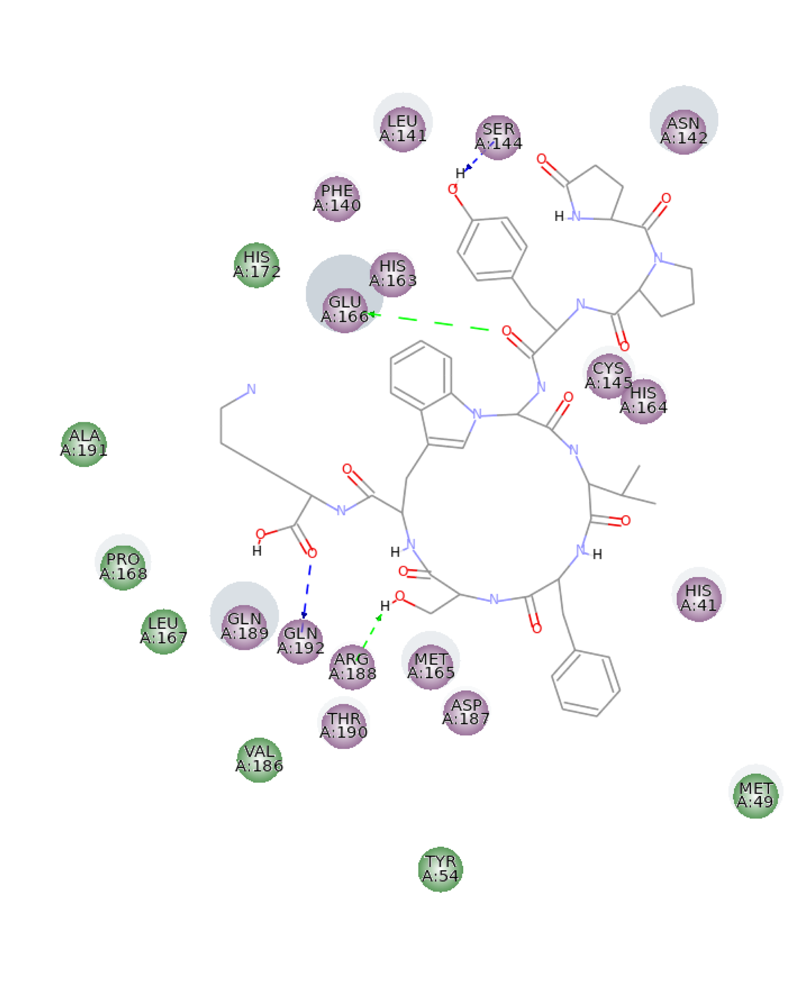 |
| 43 | Notoamide O | 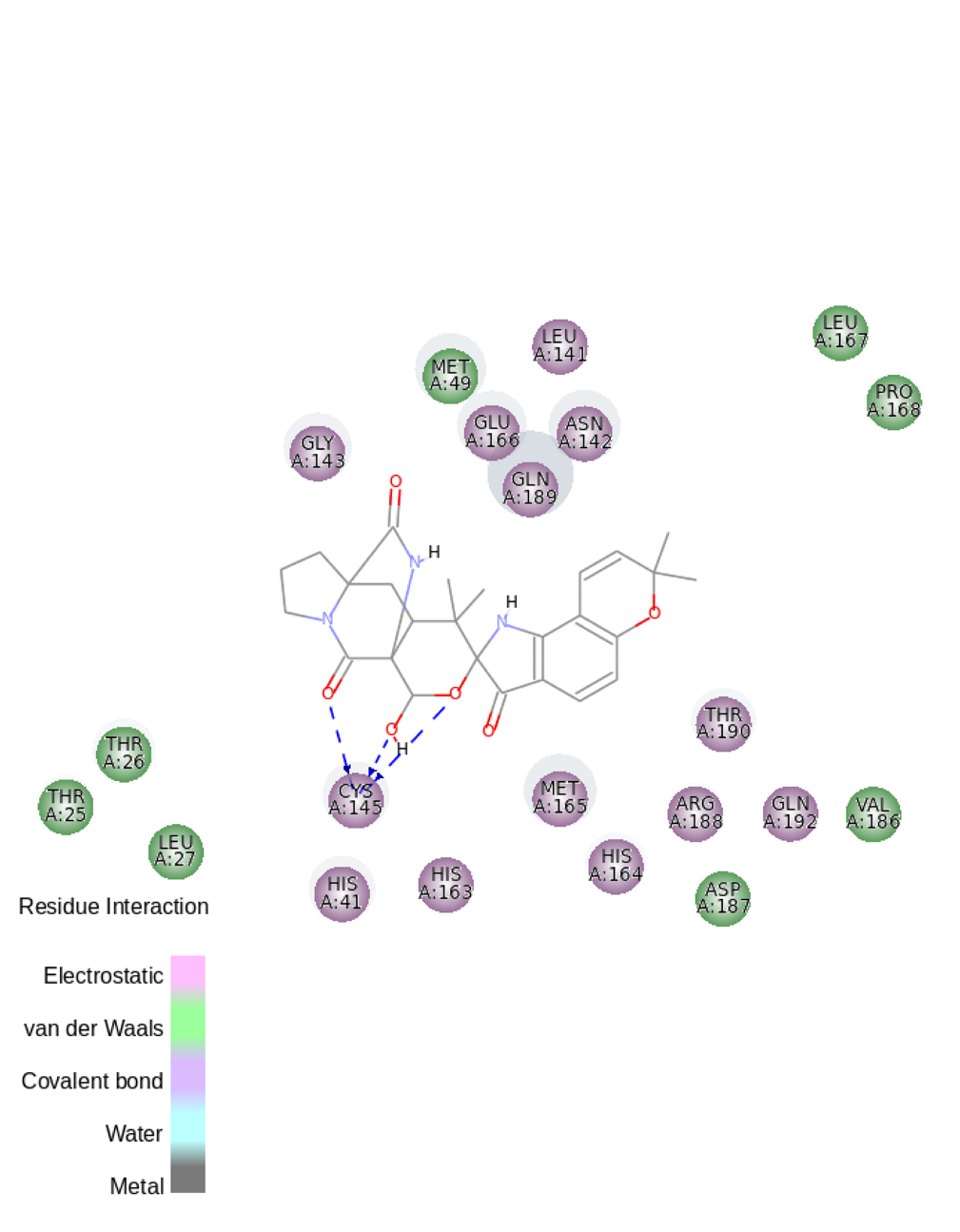 |
| 44 | Ergosine | 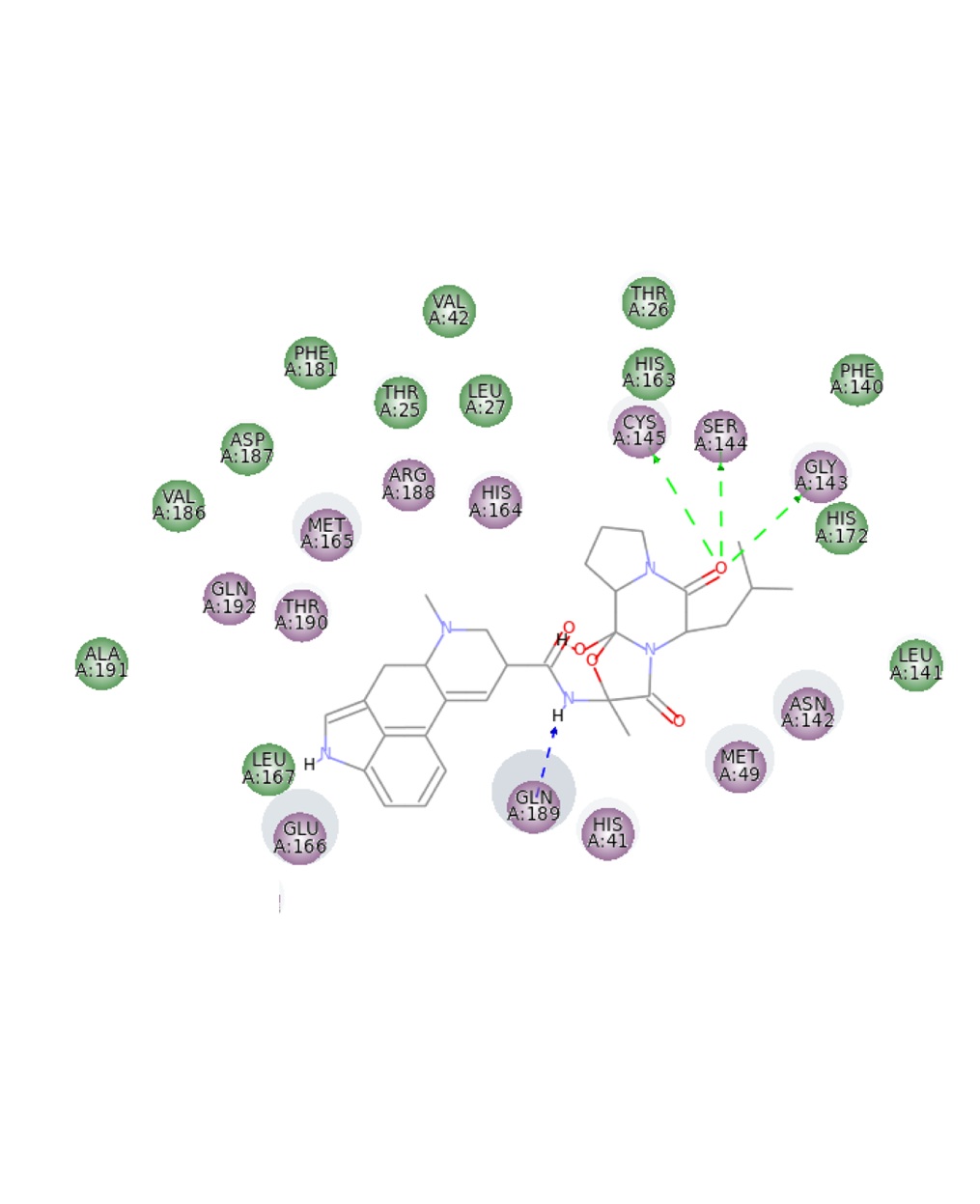 |
| 45 | Ergosinine | 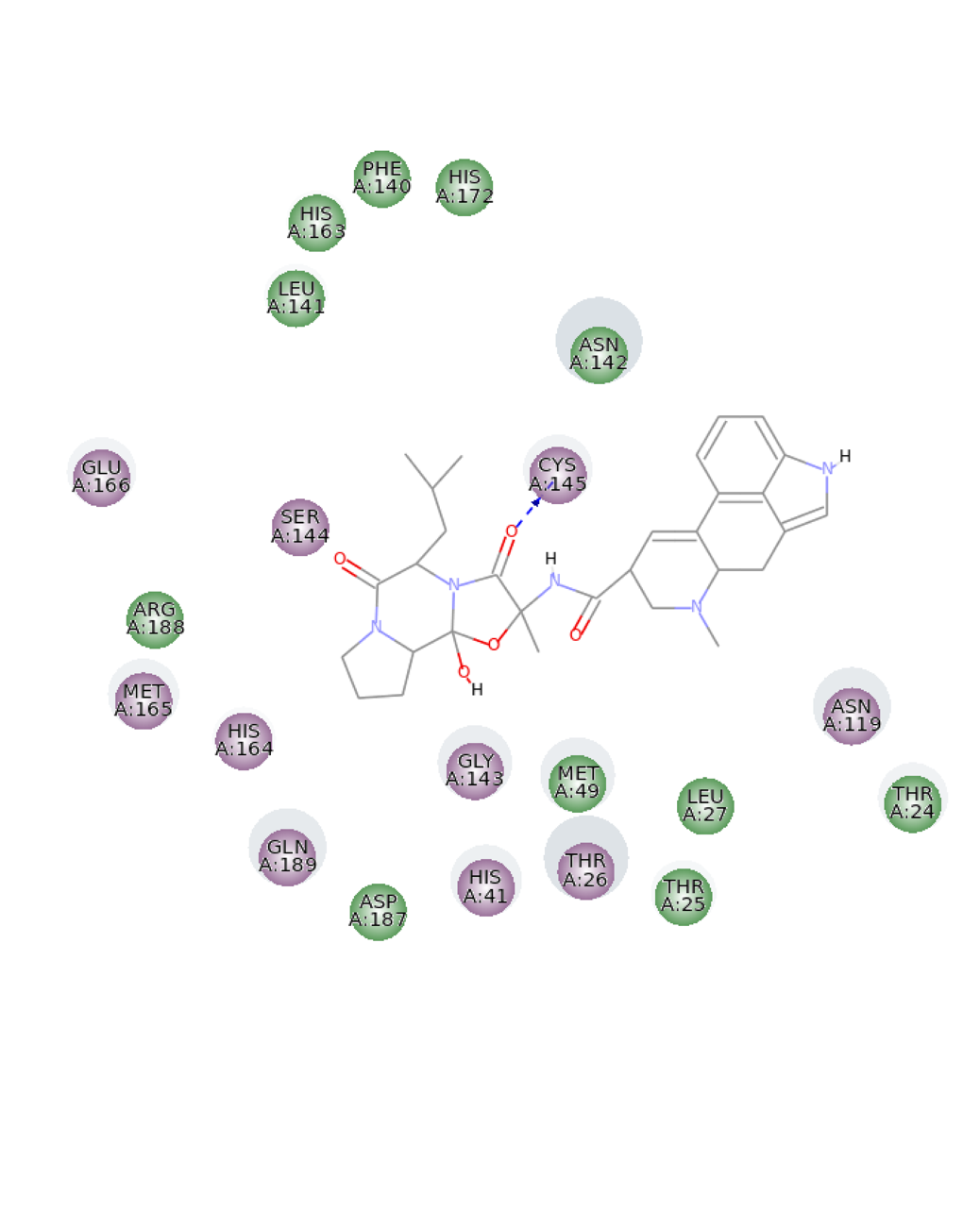 |
| 46 | Ergocornine | 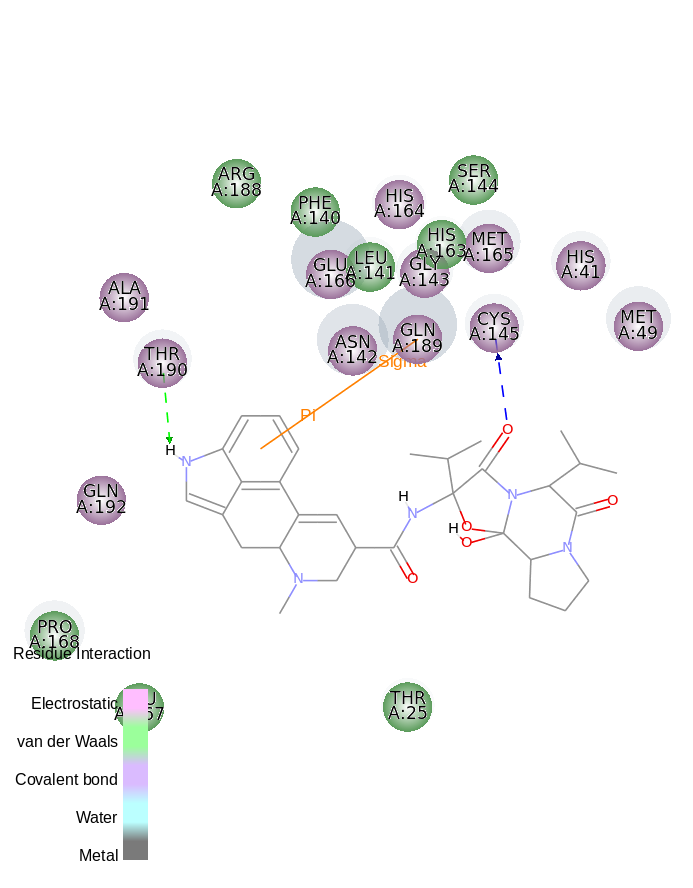 |
| 47 | Ergocryptine | 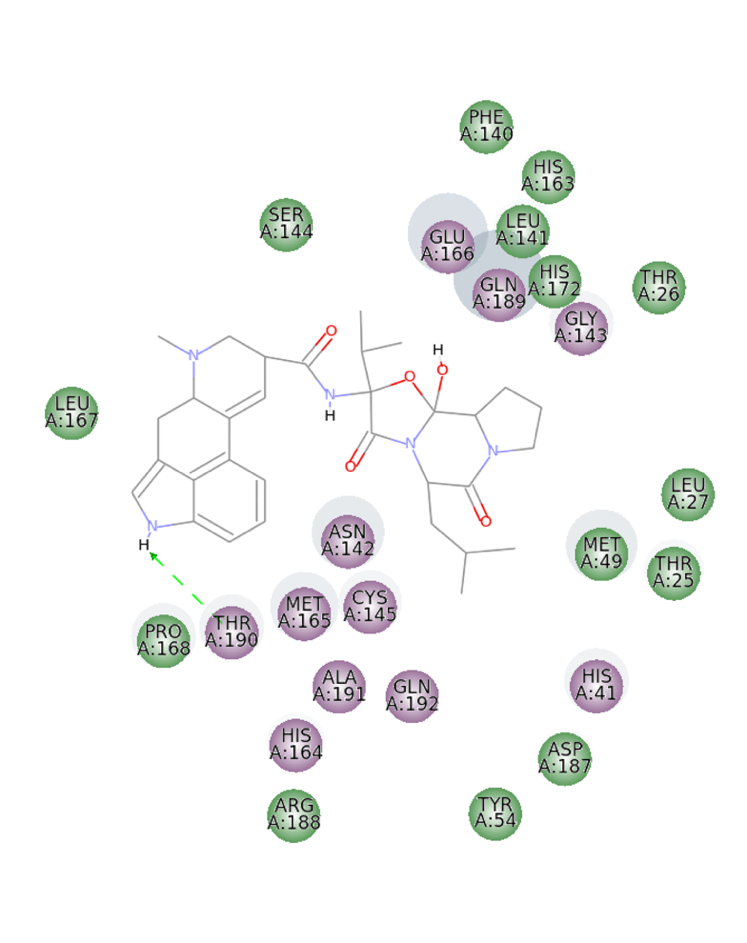 |
|  |  | SUPERNATURAL COMPOUNDS / REFERENCE MOLECULE: O6K |
| 48 | SN00109804 | 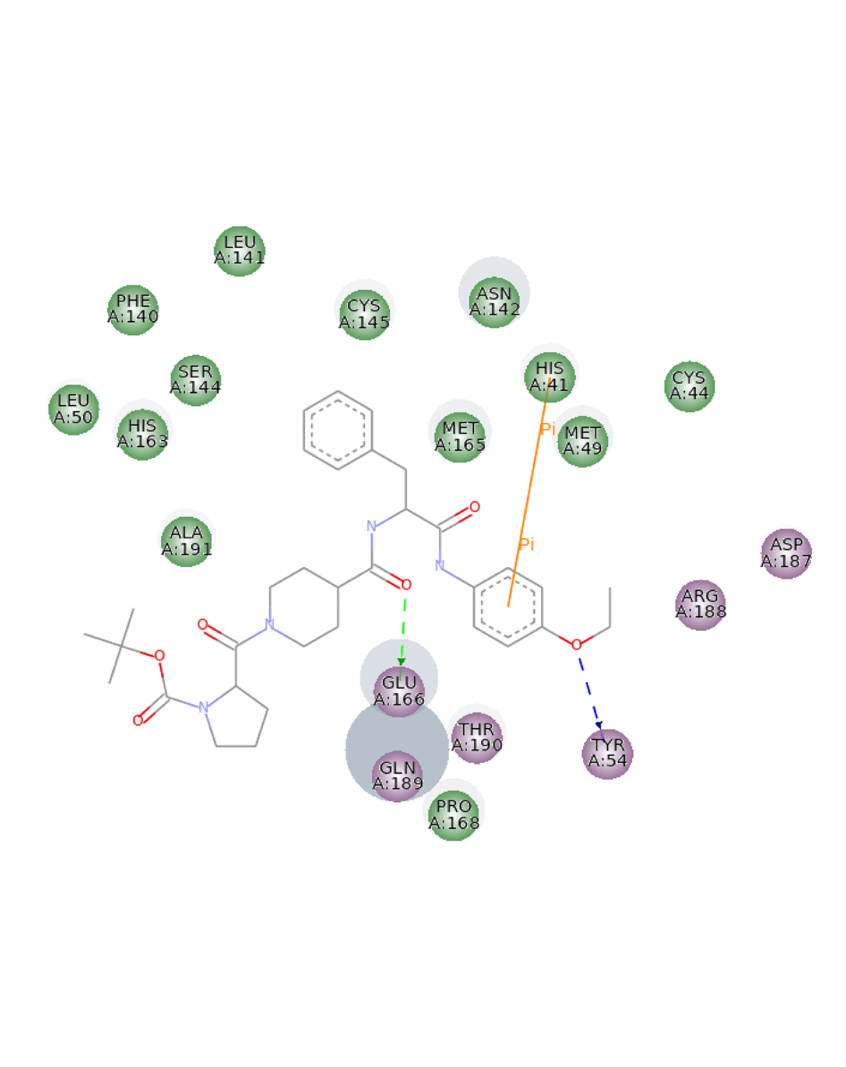 |
| 49 | SN00087725 | 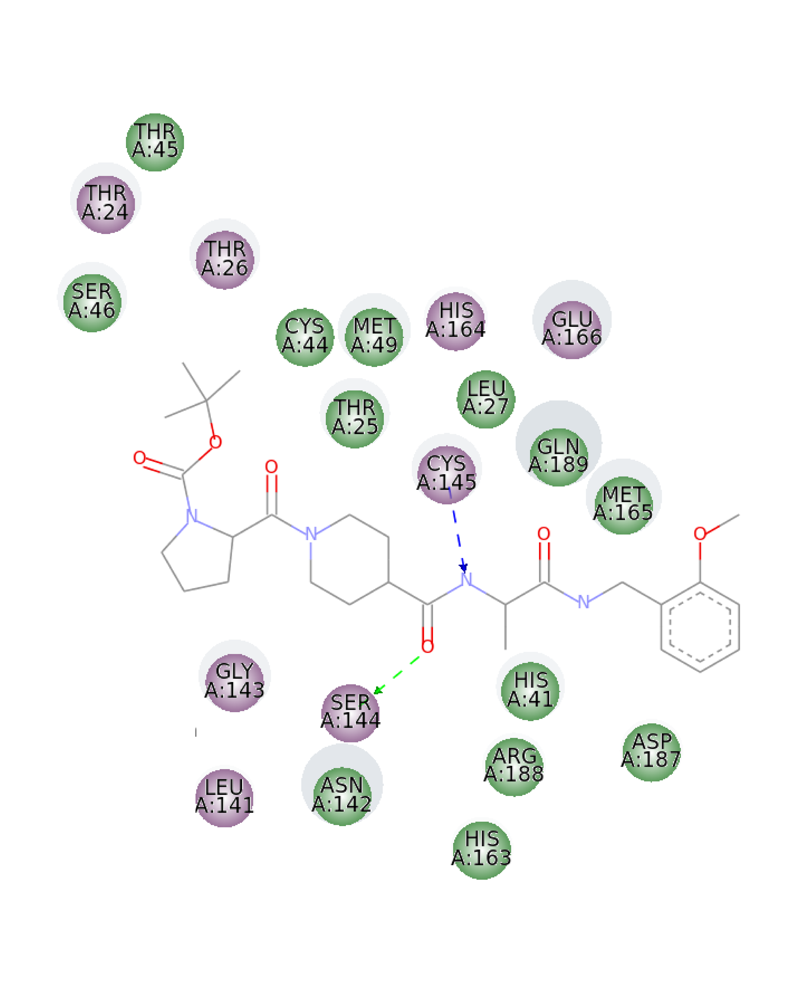 |
| 50 | SN00077453 | 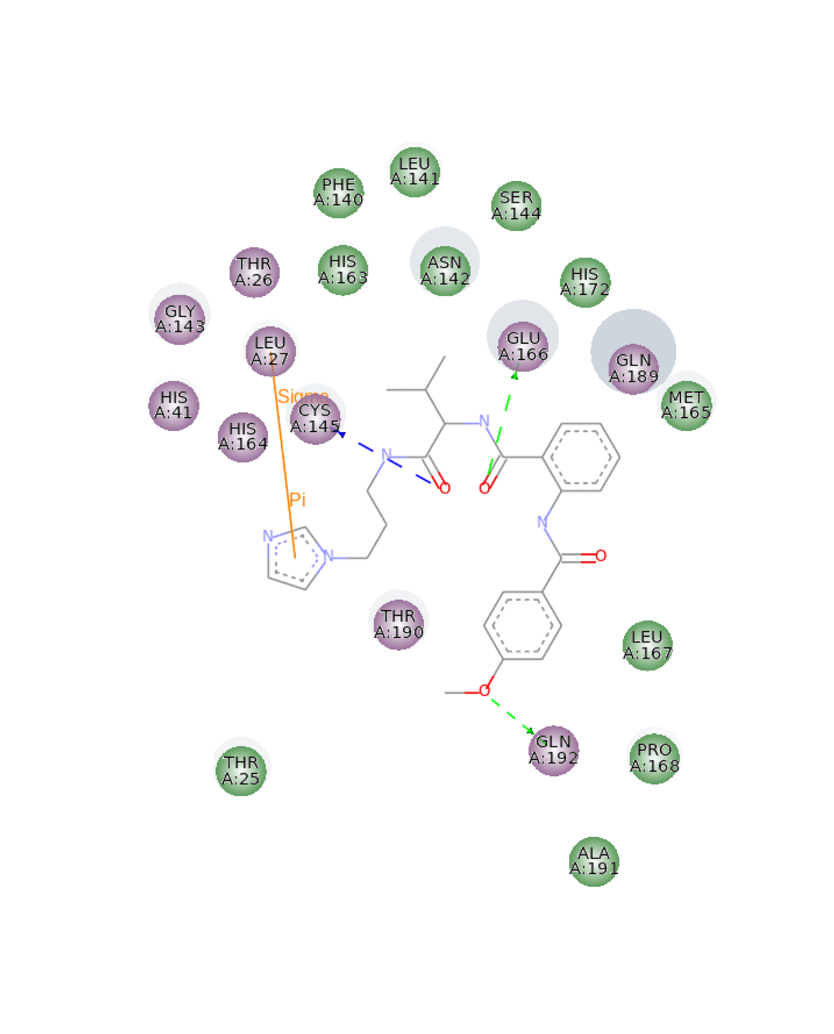 |
| 51 | SN00077454 | 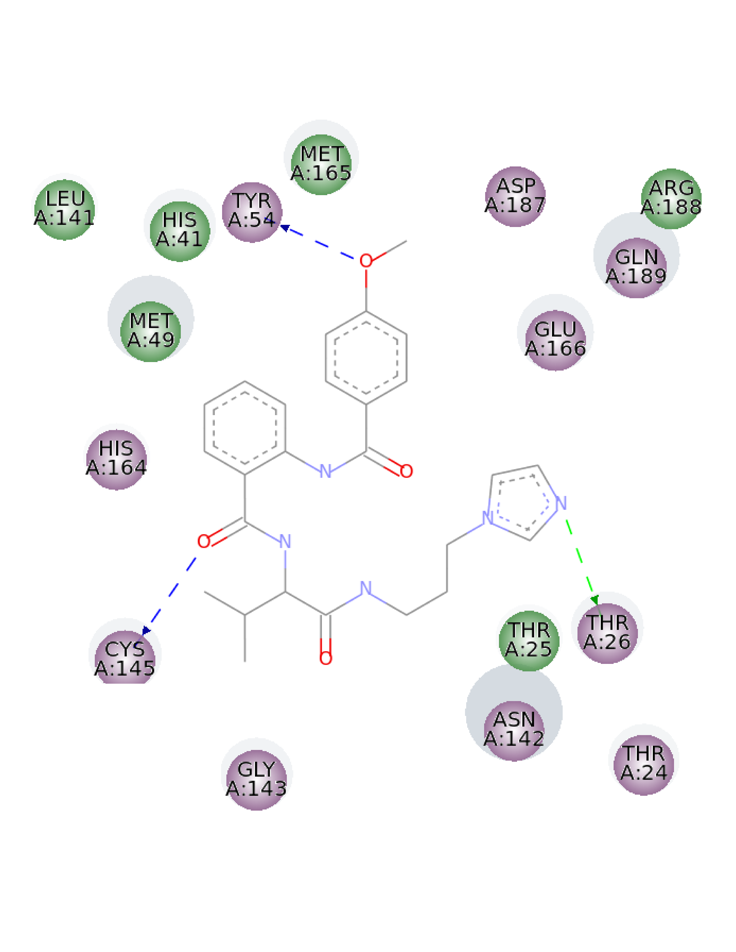 |
| 52 | SN00101312 | 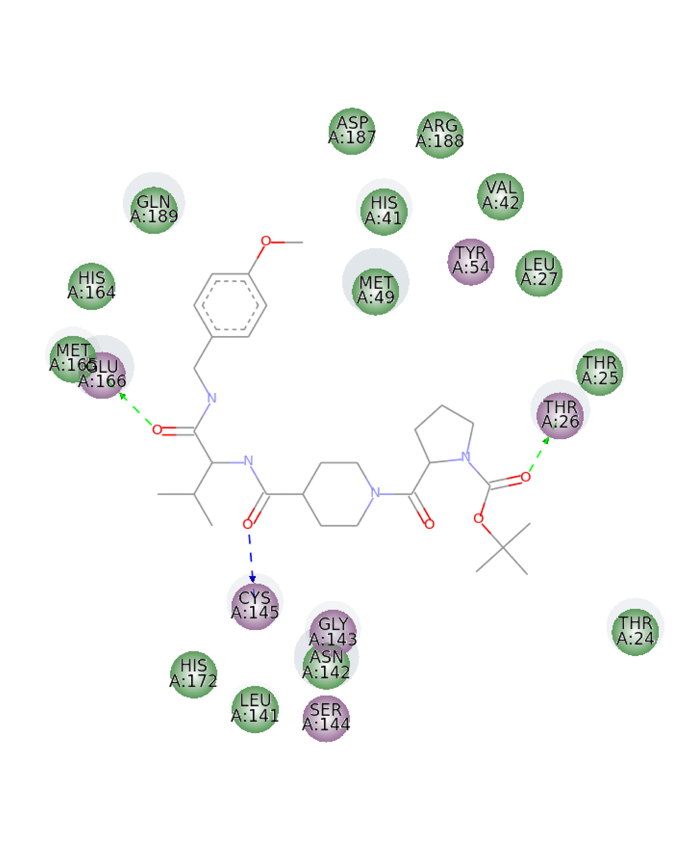 |
| 53 | SN00107903 | 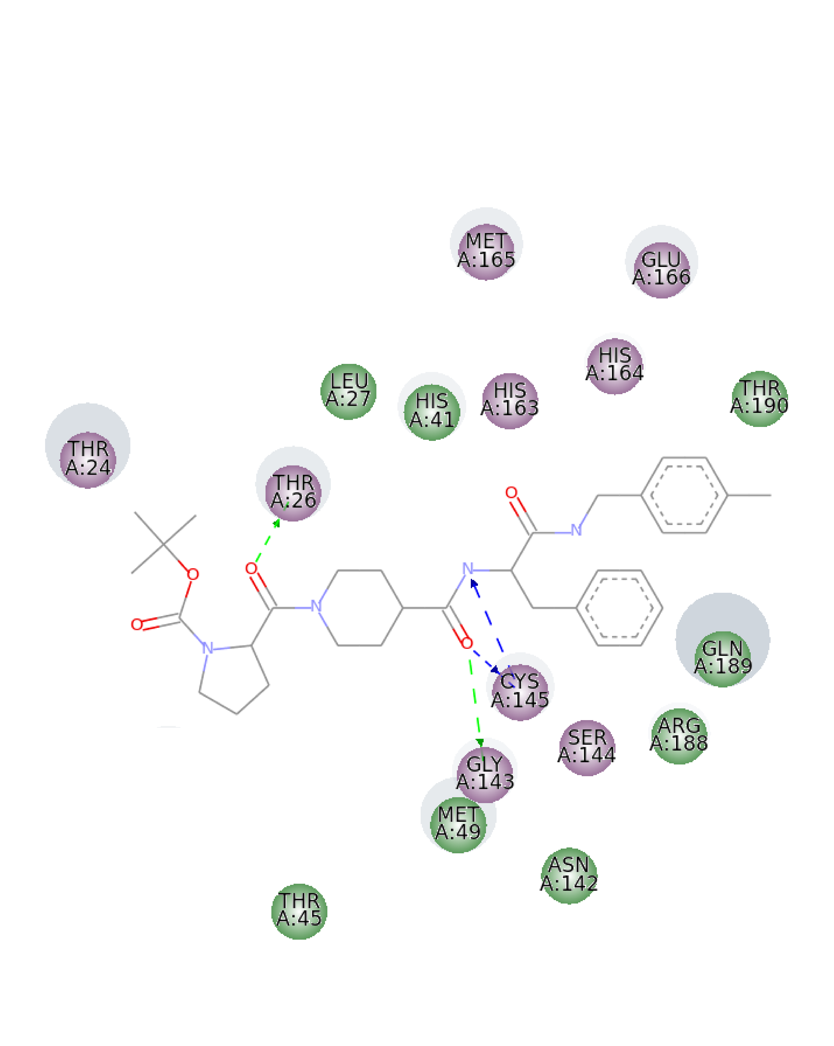 |
| 54 | SN00213824 | 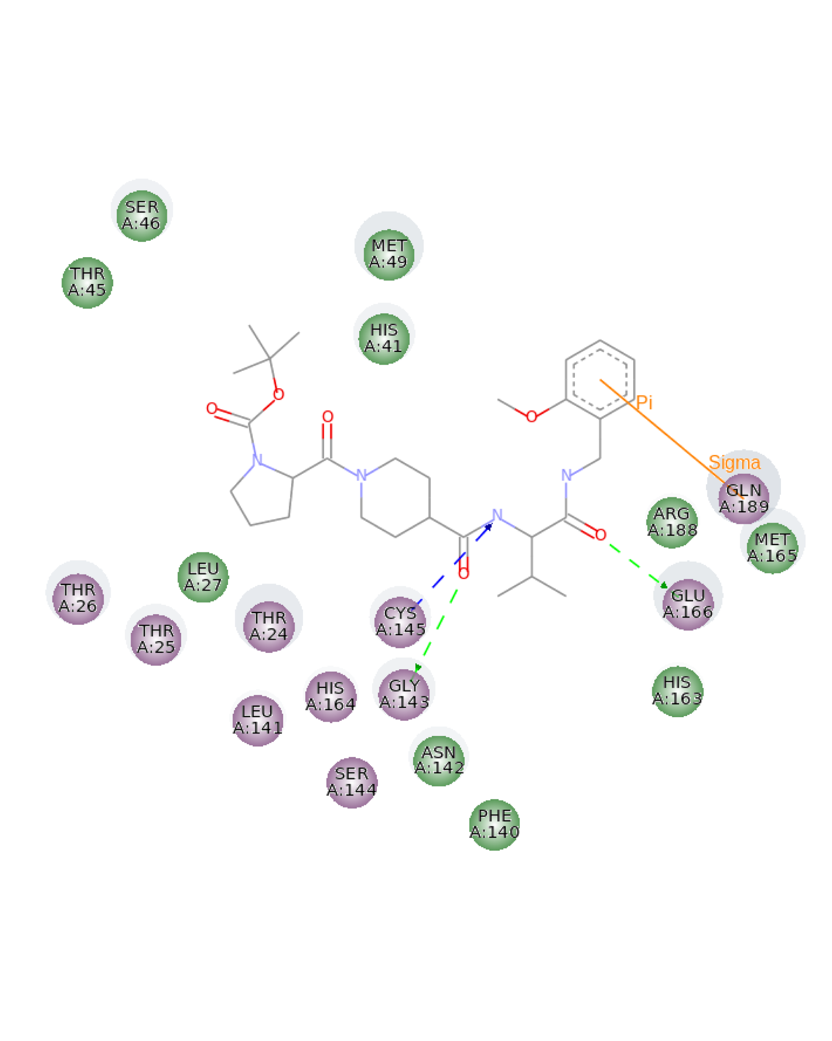 |
| 55 | SN00012917 | 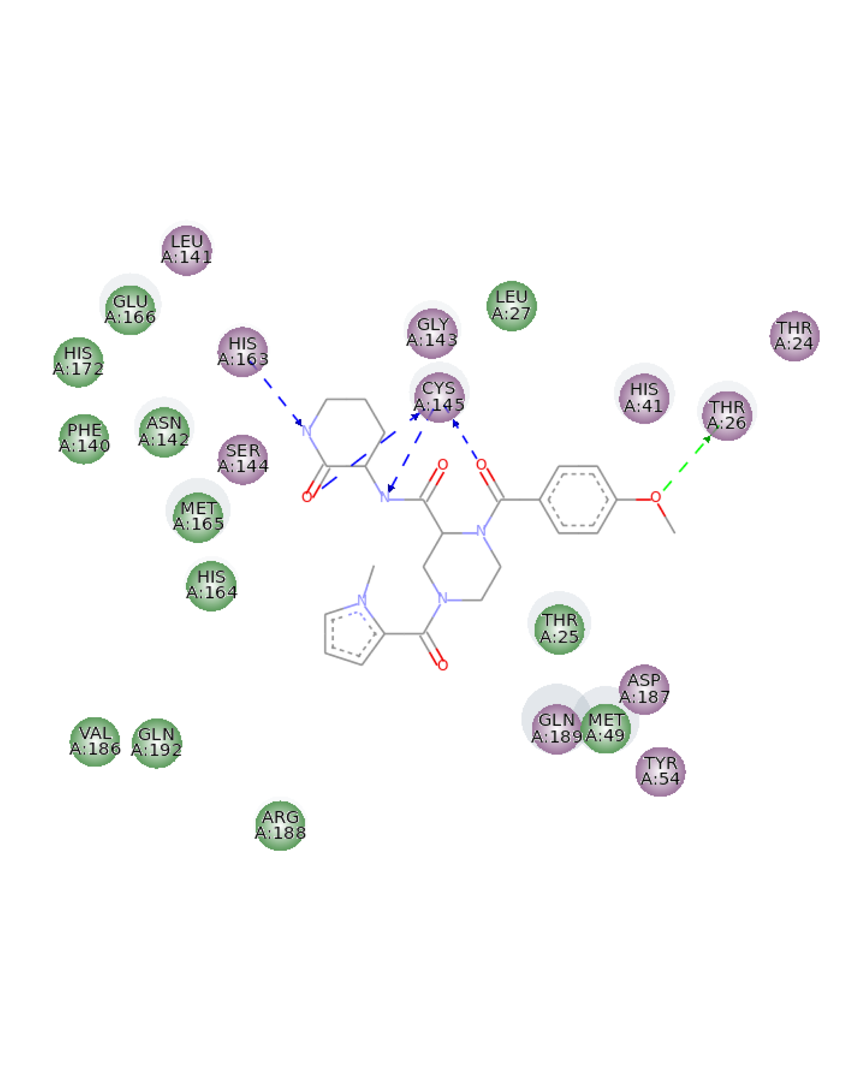 |
| 56 | SN00101324 | 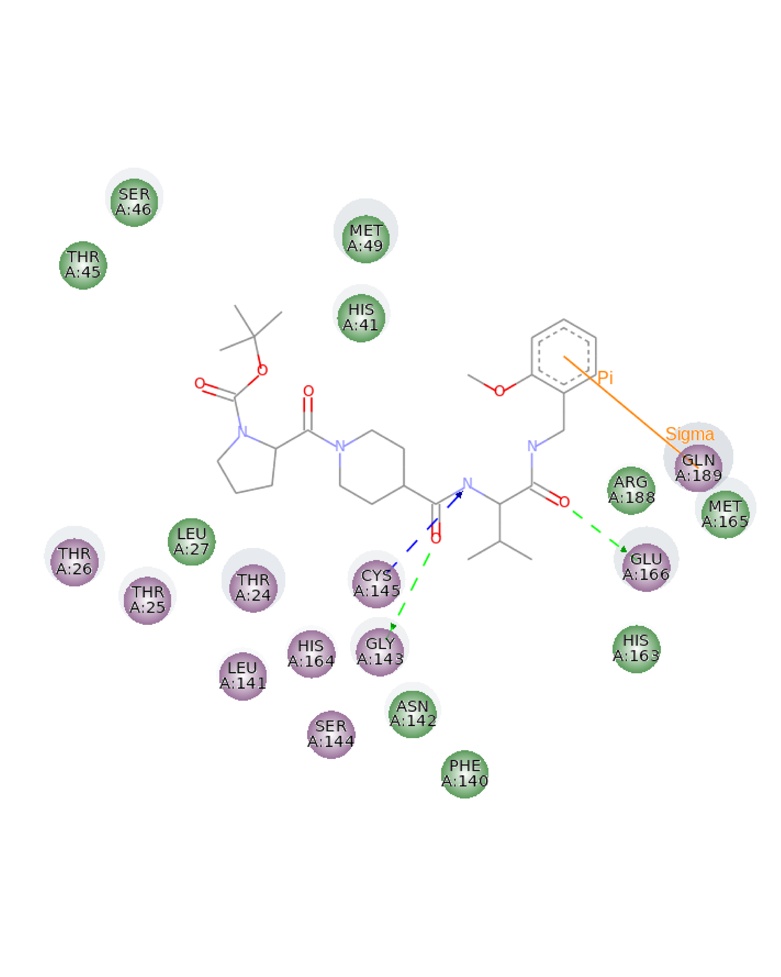 |
|  |  |  |

**Table 10 – GOLD docking scores (GoldScore fitness) for 12 best compounds and re-docked 3CLpro ligands (N3 and O6K)**

| Compound | GoldScore Fitness |
| --- | --- |
| Naldemedine | 73.66 |
| Eledoisin | 83.07 |
| Angiotensin II | 65.02 |
| Saralasin | 94.95 |
| Saquinavir | 64.48 |
| Aliskiren | 96.95 |
| Pseudostellarin C | 111.92 |
| Notoamide R | 62.01 |
| Dianthin E | 52.40 |
| SN00017653 | 55.76 |
| SN00019468 | 63.16 |
| SN00303378 | 68.70 |
| N3 ligand | 72.60 |
| 6OK ligand | 63.02 |

**Figure 1 -** protein-ligand contacts diagram for compounds from Super Natural II database

**
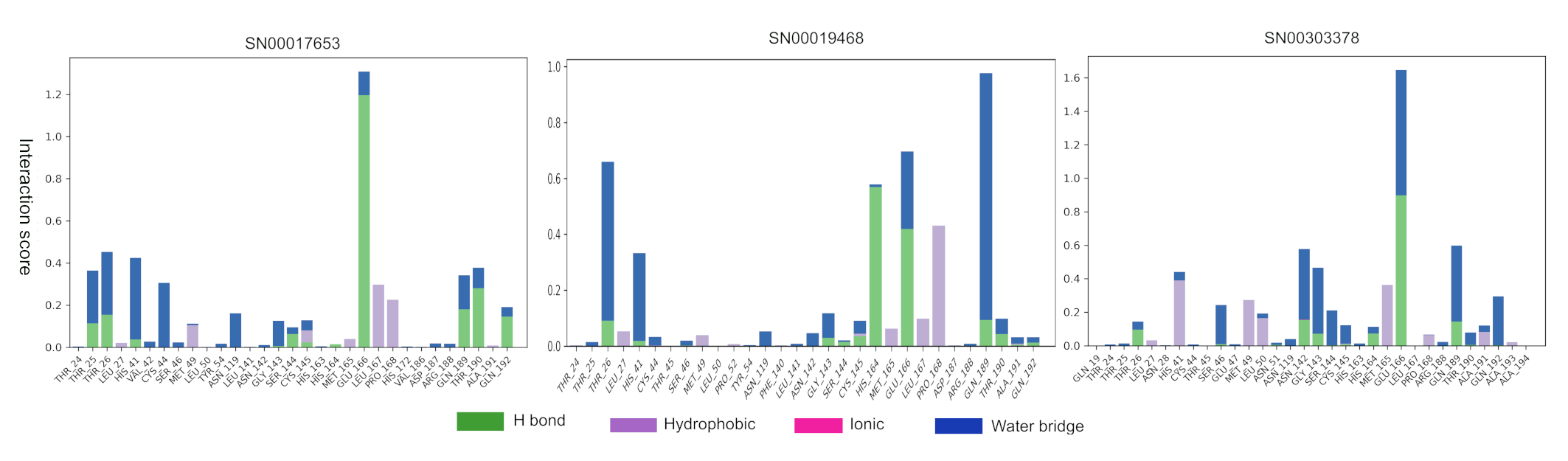
**

**Figure 2 -** protein-ligand contacts diagram for compounds from Traditional Chinese Medicine database (SuperTCM)


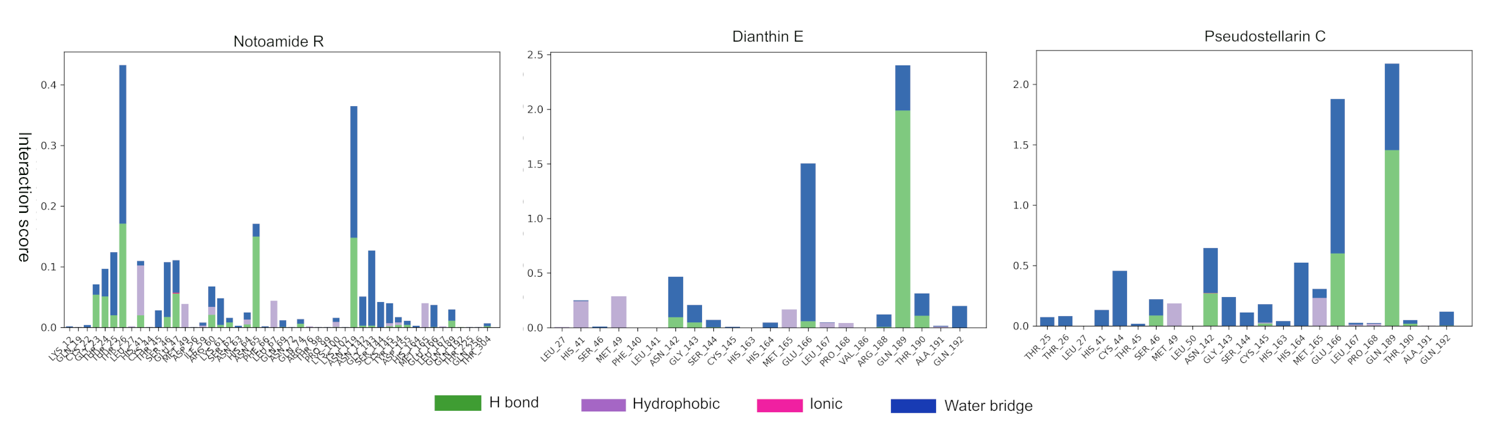


**Figure 3** protein-ligand contacts diagram for compounds from SuperDrug2 database


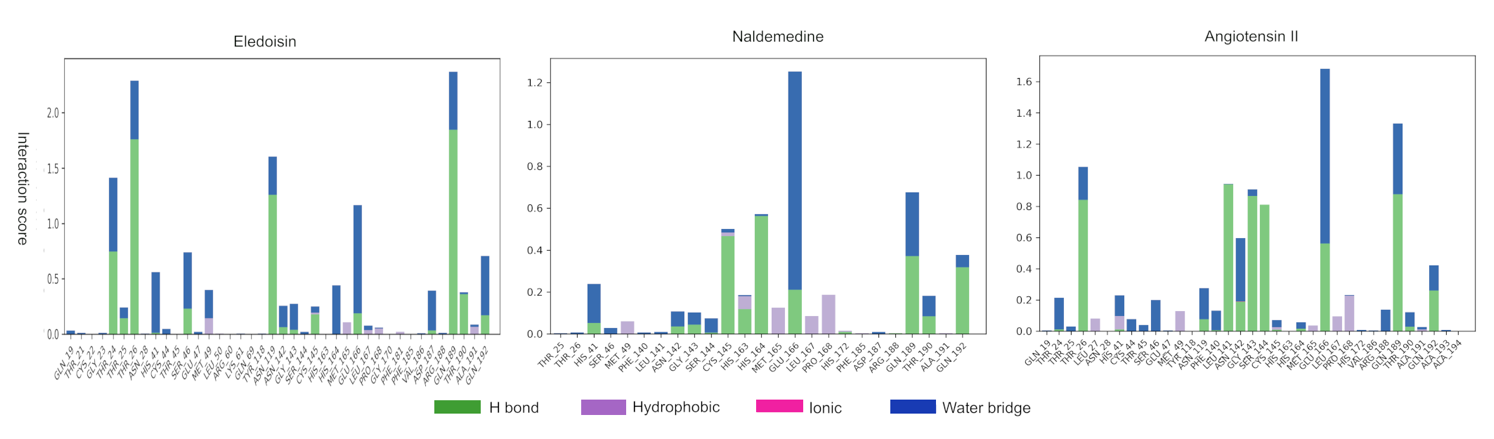


**Figure 4 -** protein-ligand contacts diagram for compounds from WITHDRAWN database


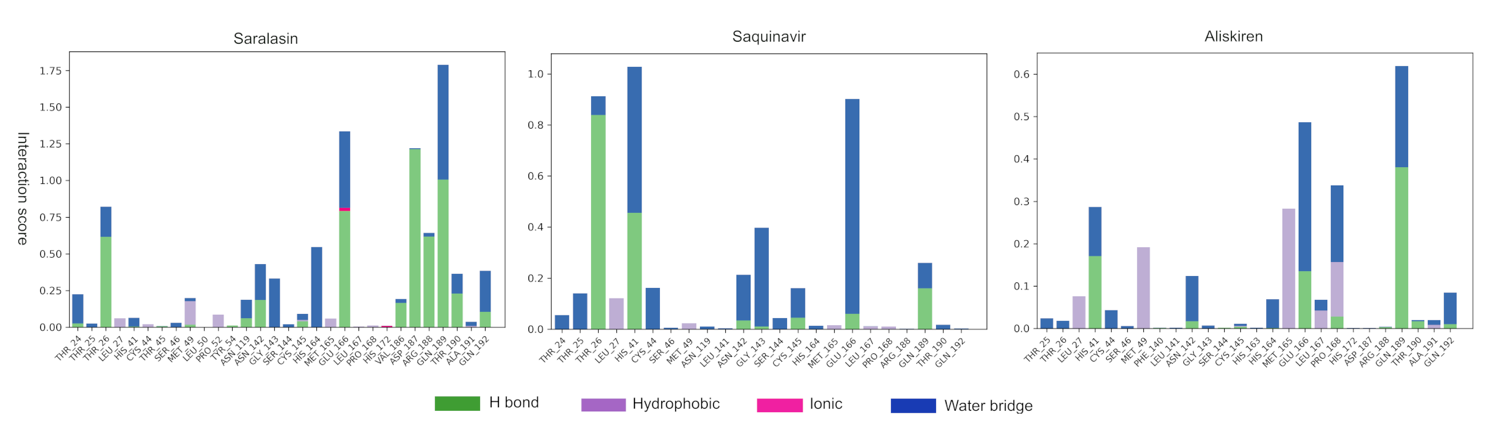


**Figure 5 –** protein-ligand contacts diagram for N3 ligand


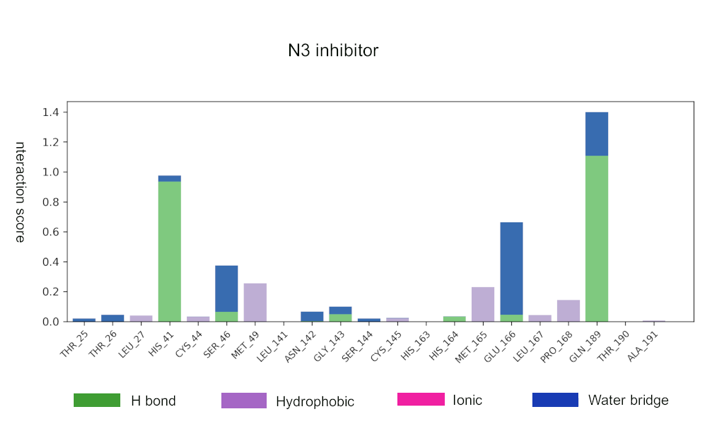


**Figure 6 -** MD simulation studies of the SARS-CoV-2 main protease with the N3 ligand.


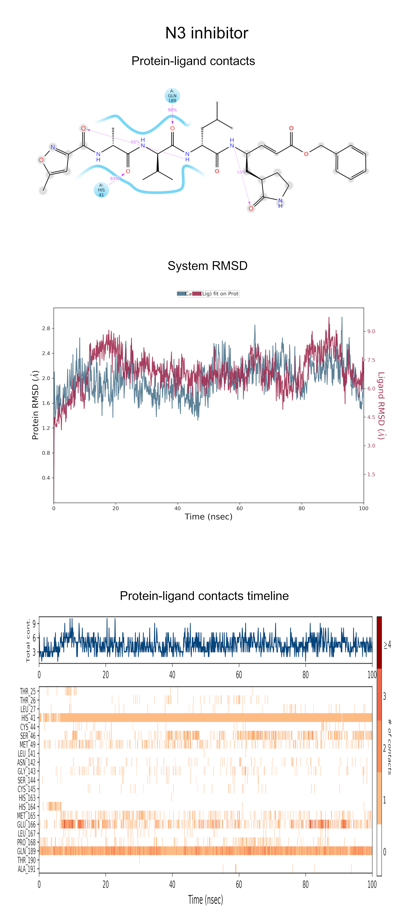

Supplement: Supplementary file 1 [file Data_Sheet_1.docx]
