# Supplementary figures and images for: Computational Prediction of Potential Inhibitors of the Main Protease of SARS-CoV-2
Source: Front Chem. 2020 Dec 23;8:590263. doi: 10.3389/fchem.2020.590263 (PMC7786237; doi:10.3389/fchem.2020.590263)

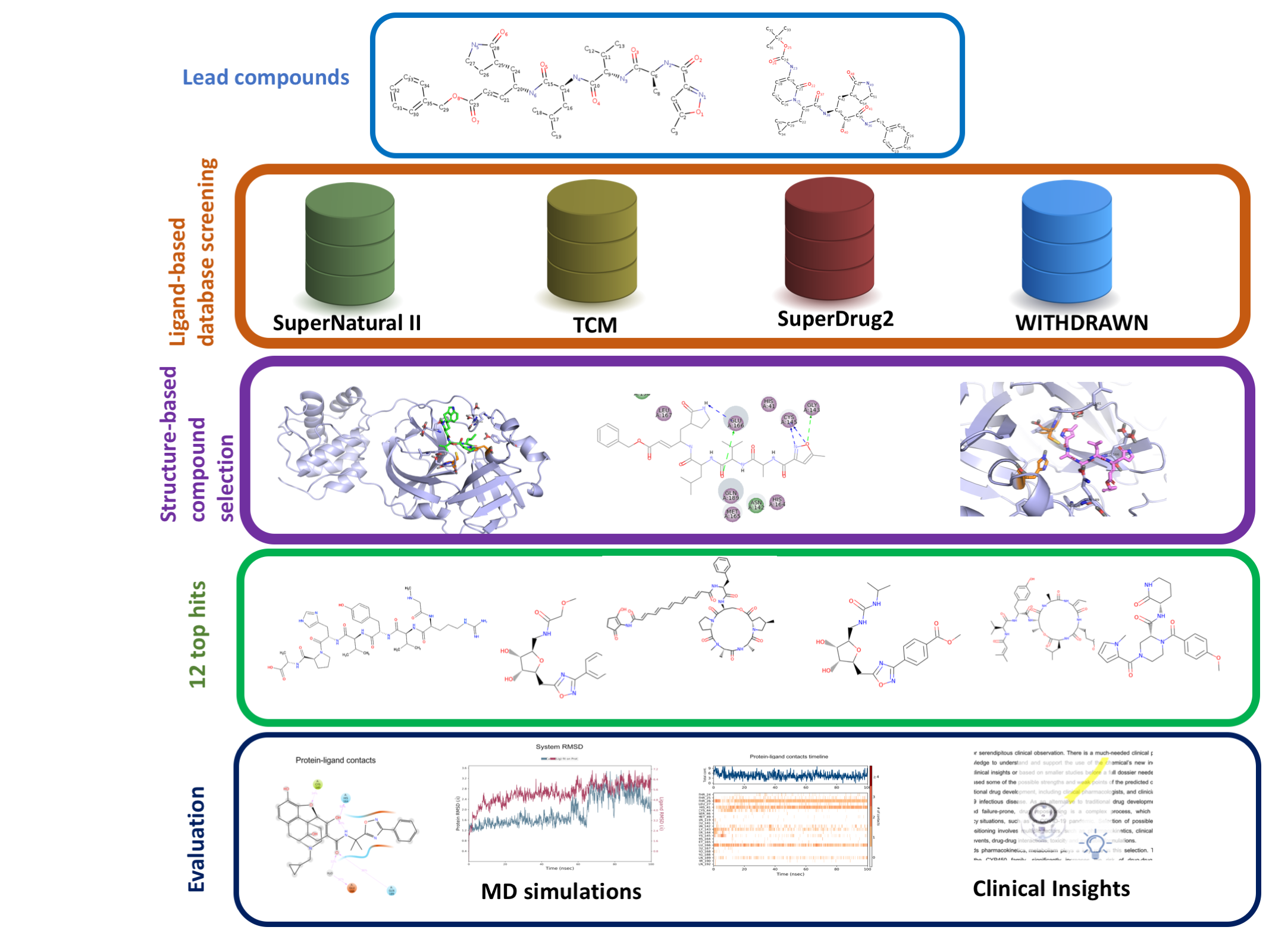

Supplement: Supplementary file 2 [file Image_1.TIFF]
